# The 9th European Conference on Marine Natural Products

**DOI:** 10.3390/md13127059

**Published:** 2015-12-03

**Authors:** RuAngelie Edrada-Ebel, Marcel Jaspars

**Affiliations:** 1 Strathclyde Institute of Pharmacy and Biomedical Sciences, University of Strathclyde, 161 Cathedral Street, Glasgow G4 0RE, Scotland, UK; 2 Marine Biodiscovery Centre, Department of Chemistry, University of Aberdeen, Old Aberdeen, AB24 3UE, Scotland, UK; m.jaspars@abdn.ac.uk

## Preface

The 9th European Conference on Marine Natural Products (ECMNP) in Glasgow follows its predecessors in La Toja (2013), Tjärnö (2011), Porto (2009), Ischia (2007), Paris (2005), Elmau (2002), Santiago de Compostela (1999), and Athens (1997). The first event in Athens in 1997 was supported by the 5th European Framework Program and the Training and Mobility of Researchers funding more than 50 fellowships which continued to the 4th Conference. From the 4th Conference in Paris to the 8th Conference in La Toja, the meetings were self-financed but equally successful with the preceding events. The 9th ECMNP in Glasgow held on the 30th of August to the 3rd of September 2015 was partly supported by three EU-FP7 consortia: SeaBioTech, BlueGenics, and PharmaSea.

The first European Conference on Marine Natural Products (ECMNP) was initiated in 1997 from Athens and organised by Professor Vassilious Roussis. After realising that European young scientists were not able to attend its major international counterpart which was mostly graced by more senior scientists, the organisers of the ECMNP have focused their attentions on stimulating the younger generation of scientists in the areas of marine chemistry to promote active interaction and future collaboration around the globe. In the European conference series, young scientists presented their work to get the experience of an international scientific atmosphere by sharing the stage with outstanding senior experts in the field who delivered the plenary lectures. It is worth mentioning that in this year’s conference, we had the participation of 94 PhD student researchers which is 50% of the entire attendance, 37 postdoc scientists; 36 senior researchers; as well as the participation of 21 industry experts from around Europe and the US which included PharmaMar SA (Spain), Ingenza Ltd (UK), Bio-Prodict BV (The Netherlands), Marine Biopolymers Limited (UK), Matís ohf (Iceland), Biosortia Pharmaceuticals (USA), Axxam SpA (Italy), Apivita SA (Greece), Pharmaq AS (Norway), NanotecMarin GmbH (Germany), and SeaLife Pharma GmbH (Austria).

A one-day free pre-conference workshop was offered for the first time to 40 early career researchers. The workshop was hosted by the Strathclyde Institute of Pharmacy and Biomedical Sciences, organised and led by SeaBioTech with support from BIOCOM AG, Berlin and PharmaSea. The aim of the workshop is to integrate and disseminate the project objectives and methodologies of five marine biotechnology FP7 consortia (MicroB3, MaCumBa, BlueGenics, PharmaSea and Seabiotech) to younger scientists involved in marine research. This was in line with the objectives of the ECMNP in promoting Marine Natural Products Research to a younger generation of scientists.

After 18 years and eight events, it is most inspiring and uplifting to perceive that many of the young scientists in the initial events are now established and leading active groups in the European marine research community. The European conference is organized every two years alternating with the Gordon Conferences on Marine Natural Products while the international MaNaPro Symposia, which remains the most important appointment for all scientists interested in Marine Chemistry, are held every three years. The conference in Glasgow welcomed 188 participants from around Europe but was also very international with scientists from US, Brazil, Chile, Costa Rica, Australia, New Zealand, Kenya, South Africa, Egypt, Saudi Arabia, Indonesia, Malaysia, Thailand, Myanmar, South Korea, and China. Under the theme: “The sea as sustainable source of new medicine and renewable energy”, our target audience included scientists both from academia and industry working not only in the field of marine natural product chemistry but also pharmacology, microbiology, biotechnology, and ecology. The conference highlighted the evolution of marine natural products chemistry to a multi-disciplinary field of research. Together with the three EU-FP7 consortia, this conference in Glasgow was led by the University of Strathclyde and jointly organized with three Scottish Universities: the University of Aberdeen, Heriot-Watt University and University of Highlands and Islands; along with the Phytochemical Society of Europe. The three industry-focused EU-FP7 consortia being under the same marine biotechnology umbrella aimed to increase the involvement and participation of SME in this conference through oral and poster presentations. There were five plenary lectures, 11 invited speakers, 43 competitively chosen oral presentations, and 123 posters. We had plenary and invited speakers from both the Industry and Academia. There were ten sessions chaired by leading scientists in their respective fields: Professor Peter Proksch on Chemical Ecology for session 1; Professor William Fenical on Deep Sea and Polar Research for session 2; Dr. Efstathia Ioannou on Marine Toxins and Bioassays for session 3; Professor Ronald Quinn on Dereplication Metabolomics, and Rational Approaches to Bioprospecting for session 4; Professor Anake Kijjoa on Isolation and Structure Elucidation for session 5; Professor Linda Harvey on Industrial Biotechnology, Polymers and Biomolecules for session 6; Dr. Carmen Cuevas on Organic Synthesis for session 7; Professor Gabriel Koenig on Biosynthesis of Marine Natural Products in Microbes for session 8; Professor Hartmut Laatsch on Marine Microbes/Fungi on session 9; and Professor Marcel Jaspars on Marine Policy for session 10. The session on Marine Policy was put together and was delivered by experts to provide greater awareness on international laws of bioprospecting covered by UNCLOS (United Nations Convention on the Law of the Sea) and the Nagoya Protocol. Each day of the conference was opened by a plenary lecture while every session started with an invited lecture from an established scientist and progressed on with five 10-min oral presentations from early career researchers.

Professor Dr. Joern Piel from ETH Zurich gave the opening plenary which appropriately provided the marine natural products research community with insights into the function of bacteria in various sponges. He presented examples of biosynthetic studies on selected sponge natural products and their practical implications for the generation of sustainable production systems. The key word “sustainability” led to the ecology topics for the first day of the conference. Dr. Joanne Porter from Heriot-Watt University steered the marine ecology session pointing out the marine ecological perspective on future exploitation of marine natural products. Dr. Porter focused on the under-investigated phylum Bryozoa with the discovery and development of bryostatin from uncultured *Endobugula sertula* bacterium associated with larvae of *Bugula neritina*. The ecological interaction ultimately leads to the identification of a wider range of novel compounds by investigating the organisms’ growth strategies. Close working relationships between chemists and marine ecologists is likely to lead to a deeper understanding of the functional role of compounds in the marine environment. Topics for short oral presentations followed the trend for discussion on the role of secondary metabolites in defence and evolution, chemical interactions between toxic microalgae and Mediterranean benthic diatoms, as well as dual induction of microbial secondary metabolites by fungal/bacterial co-cultivation. Professor Conxita Avila from the University of Barcelona expanded the topic on chemical ecology to the Antarctic region by demonstrating the high biological and chemical diversity found in the extreme and unique marine environment. This was complemented by the oral presentations on the isolation and bioactivity screening of unique compounds and extracts from both deep sea and Polar Regions. Chemical diversity can be bio-prospected through innovative bioassays like using zebrafish as a biomedically relevant model for functional genomics and *in vivo* drug discovery as introduced by Dr. Camilla Esguerra from the University of Oslo. Dr. Esguerra described the *in vivo*, microgram-scale, high-throughput bioassays based on zebrafish embryos and larvae for a rapid, systematic identification, and pharmacological characterization of bioactive natural products. Short oral presentations on the session covered mechanism of action of potential novel anti-cancer agents and microalgal toxins. The first day ended with a plenary lecture by Professor Heinz Schröder from the Johannes Gutenberg University of Mainz. Professor Schröder introduced their pioneering work with Professor Werner Mueller on the utilisation of bio-silica and the enzyme silicatein in the synthesis of polymeric silica for the development of novel bioinspired materials for diverse applications in nano-optics and nano-biotechnology.

The second day of the conference focused on the chemistry of new bioactive natural products and the rational approach of bioprospecting them. It commenced with the plenary lecture by Dr. Guy Carter from Biosortia Pharmaceuticals which presented the industry’s view on drug discovery from marine natural products (MNP). Screening of MNP for pharmaceutical discovery became the focus of many academic groups, but only a few Pharma companies have taken the plunge. Dr. Carter highlighted remarkable developments and success stories of MNP going into the pharmaceutical industrial pipeline. Professor Kristian Fog Nielsen from the Technical University of Denmark opened the session on dereplication and metabolomics with the application of high resolution mass spectrometry and mass fragmentation libraries to assist in the discovery of novel compounds which is vital for strain prioritization for further isolation work. Target-directed dereplication approaches that efficiently accelerate the isolation of bioactive compounds were introduced during the session. This included metabolomics tools utilising both NMR spectroscopy and mass spectrometry that could be further visualised through molecular networking of the mass fragmentation data. Genome sequencing techniques was also shown to evaluate the potential of isolates to produce bioactive compounds by screening the presence of genes encoding for non-ribosomal peptide synthetases (NRPSs) and polyketide synthases (PKSs). Further on to the isolation and structure elucidation of bioactive secondary metabolites, Dr. Teodor Parella from the Autonomous University of Barcelona presented exciting novel 2D NMR techniques that increased signal resolution to elucidate complex structures by collapsing typical proton multiplet resonances. NMR, mass spectrometry, and circular dichroism techniques to solve stereochemistry and absolute configuration of small molecules were further delved into through the short oral presentations. The subsequent sessions dealt with scale-up production of bioactive molecules through industrial biotechnology and organic synthesis. 

Marine natural products do not only consist of small molecules but as well as larger polymers (e.g., polysaccharides) and biomolecules which were themes under industrial biotechnology. The session was preceded by the lecture of Professor Guðmundur Óli Hreggviðsson from the University of Iceland on the application of novel enzymes from thermophilic microorganism in macroalgal biorefineries. The session was dominated by collaborative work with industry. The session covered scale-up production of small molecules in microbes and protein engineering of novel enzymes through sophisticated software platforms to develop faster biosynthetic routes for bio- and small molecules. Parallel to bioprocessing technology, organic synthesis is to date still widely used to increase the yield of bioactive metabolites as well as foremost to optimise the bioactivity of a molecule. Dr. Gordon Florence from the University of St Andrews commenced the session on organic synthesis by presenting his work on the synthesis of oxazole-containing natural products and the development of natural product inspired inhibitors of *T. brucei*. The session exemplified the organic synthesis of some bioactive analogues for structure-activity-relationship (SAR) studies as well as the further application of metabolomics in targeting drug lead cryptic analogues. One oral contribution presented the use of synthetic standards to carry out *J*-based configurational analysis which plays an important role in SAR analysis.

The theme for the third day of the conference was on natural products from marine microbes. The final day of the conference was opened with a plenary lecture by Professor William Gerwick from University of California San Diego, highlighting his work on cyanobacteria. Professor Gerwick introduced an innovative approach to discover new drug leads which involves a combination of technologies *i.e.*, genome-based information, mass spectrometry-based Molecular Networks, and synthetic medicinal chemistry. He has described this as a “Top Down *versus *Bottom Up” approach. For the session on biosynthesis, Professor Angelo Fontana from the National Research Council Italy described his work on the biosynthesis of secondary metabolites in marine diatoms and dinoflagellates, use of phytoplankton for development of new drugs as well as the elucidation of biochemical pathways related to the production of renewable energy which was very appropriate to the theme of the conference. The session continued with the introduction of metagenomics approach to bioprospect microorganisms possessing large number of genes from metabolic pathways relevant for biotechnology, while further techniques were put together to identify novel biosynthetic pathways in uncultivated sponge symbionts. The last scientific session explored on bioactive bacterial and fungal secondary metabolites from extreme marine environments. Professor Alan Dobson from University of Cork led the session with his short lecture on mining novel bioactive compounds from sponge-associated microorganisms for biopharmaceutical applications by utilising genomic, metagenomic, and culture-based approaches. The short oral presentations looked into the chemical diversity of bioactive natural products from marine microbes isolated from various marine sources.

The final session of the conference highlighted the importance of recent changes in marine policy and the potential impact on carrying out natural products discovery and exploitation. The session was structured into three invited presentations followed by an extended open discussion. Dr. Thomas Vanagt from eCoast Belgium introduced the topic of the Nagoya Protocol and explained the obligations on scientists to ensure they have legal certainty over the materials collected from within national jurisdictions. This was complemented with a presentation by Professor Jaspars from the University of Aberdeen on the recent decision by the UN to develop a new implementing agreement to the UN Convention on Laws of the Sea covering the fair and equitable use of biodiversity from areas beyond natural jurisdiction. He gave three different scenarios where such an agreement might be employed and a scientific rationale justifying the preferred option presented. Dr. Olga Genilloud from Fundacion Medina Spain presented an industrial view of access and benefit sharing gained from a number of years working in the pharmaceutical industry. This session ended with a vigorous discussion in which some points were clarified while others raised further discussion with the relevant policy advisory committees.

The conference culminated with a plenary lecture by the Phytochemical Society of Europe’s Bruker award prize winner, Professor Roussis of the University of Athens. He gave a fabulous presentation on the search for algal metabolites with biomedical-biotechnological potential from the eastern Mediterranean. The presentation perfectly encapsulated many of the themes addressed by the conference: how an understanding of the taxonomy and chemical ecology of an organism may assist in the directed search for new products and processes; how sophisticated analytical and bioinformatic techniques can speed the process of discovery and finally how such materials may find applications in a range of fields from pharmaceuticals through antifoulants to biomedically important materials.

The 9th ECMNP in Glasgow was successful and met its objective to promote marine natural products research to younger generation of scientists who will lead the future research in marine chemistry and its adjunct fields in ecology, molecular biology, pharmacology, microbiology, and biotechnology. In this conference, we have witnessed early career researchers developing innovative approaches in the discovery of new marine drugs and these new generation of scientists will lead marine natural products research in the future.

### ***Acknowledgments*** 

This work was supported by grants from the European Commission within its FP7 Programme, under the thematic area KBBE.2012.3.2-01 with Grant Number Nos. 311932 “SeaBioTech”, 311848 “BlueGenics”, and 312184 PharmaSea.

## Session 1: Plenary Speaker Abstracts

### ***Natural Product Pathways from Sponge Symbionts*** 

**Jörn Piel**

Institute of Microbiology, ETH Zurich, Switzerland

Sponges are among the richest sources of bioactive natural products from marine habitats. Since many sponges harbor diverse bacterial communities, it has long been suspected that many sponge-derived compounds are of microbial origin. For a diverse range of complex polyketides [1], ribosomally modified peptides, and nonribosomal peptides from the sponge *Theonella swinhoei*, “Entotheonella” bacteria were identified as source using metagenomic and single-bacterial analysis. “Entotheonella” belong to a new, uncultivated candidate phylum termed “Tectomicrobia” and exhibit a rich and variable specialized metabolism involving unusual biosynthetic steps. Functional characterization of enzymes suggests that “Entotheonella” spp. offer interesting biotechnological opportunities in addition to their high drug discovery potential. The talk presents methods to study “Entotheonella” and provides insights into the function of these bacteria in various sponges. In addition, biosynthetic studies on selected sponge natural products are discussed, as well as practical implications for the generation of sustainable production systems [2,3].


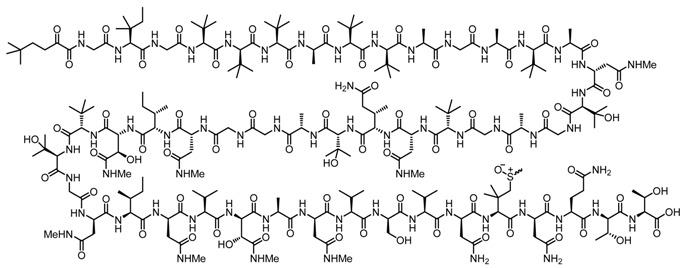


Polytheonamides from “Entotheonella factor”, a symbiont of the sponge *Theonella swinhoei*

#### **References** 

1. Ueoka, R.; Uria, A.R.; Reiter, S.; Mori, T.; Karbaum, P.; Peters, E.E.; Helfrich, E.J.N.; Morinaka, B.I.; Gugger, M.; Takeyama, H.; *et al*. Metabolic and evolutionary origin of actin-binding polyketides from diverse organisms. *Nat. Chem. Biol.*
**2015**, *11*, 705–712.
2. Wilson, M.C.; Mori, T.; Rückert, C.; Uria, A.R.; Helf, M.J.; Takada, K.; Gernert, C.; Steffens, U.A.E.; Heycke, N.; Schmitt, S.; *et al*. An environmental bacterial taxon with a large and distinct metabolic repertoire. *Nature*
**2014**, *506*, 58–62.
3. Freeman, M.F.; Gurgui, C.; Helf, M.J.; Morinaka, B.I.; Uria, A.R.; Oldham, N.J.; Sahl, H.-G.; Matsunaga, S.; Piel, J. Metagenome Mining Reveals Polytheonamides as Posttranslationally Modified Ribosomal Peptides. *Science*
**2012**, *338*, 387–390.

### ***The Promise and Perils of Marine Natural Products as Leads for Drug Discovery*** 

**Guy Carter**

Biosortia Pharmaceuticals, Dublin, OH, USA

For natural product research scientists, the discovery of elaborate and often exotic metabolites provides sufficient intellectual challenge to drive the exploration of the biosphere for novel products. For several decades marine natural products (MNP) have served as an important new source of chemical diversity. In the early days of MNP research the promise was that the oceans would be the next great source of new medicines—replacing much explored terrestrial plants and microorganisms. The screening of MNP for pharmaceutical discovery became the focus of many academic groups, however only a few Pharma companies took the plunge. The rate of discovery of new MNP continues to increase on an annual basis yielding over 1000 new compounds per year, most of which have been reported as biologically active [1]. Now nearly 50 years since the first Drugs from the Sea conference [2], the expected cornucopia of new marine drugs is scarcely populated [3]. Where is the disconnect? In this presentation I will take a critical look at the reasons why the Promise has not yet been fulfilled as well as highlight notable developments and success stories.

#### **References** 

1. Blunt, J.W.; Copp, B.R.; Keyzers, R.A.; Munro, M.H.G.; Prinsep, M.R. Marine natural products. *Nat. Prod. Rep.*
**2014**, *31*, 160–258.
2. Freudenthal, H.D. (Ed.) *Proceedings of “Drugs from the Sea” Conference*; Marine Technology Society of the United States: Kingston, RI, USA, 1967.
3. Newman, D.J.; Cragg, G.M. Marine-sourced anti-cancer and cancer pain control agents in clinical and late pre-clinical development. *Mar. Drugs*
**2014**, *12*, 255–278.

### ***Top Down *versus* Bottom Up: Orthogonal Approaches for the Discovery of New Bioactive Natural Products from Marine Cyanobacteria*** 

**Alban Pereira ^1^, Jehad Almaliti ^1^, Karin Kleigrewe ^1^, Robin Kinnel ^1,2^, Anton Korobeynikov ^3^, Vincenzo Di Marzo ^4^, Lena Gerwick ^1^ and William Gerwick ^1^**

^1^ Scripps Institution of Oceanography and Skaggs School of Pharmacy and Pharmaceutical Sciences, University of California San Diego, 9500 Gilman Drive MC 0212, La Jolla, CA 92093, USA

^2^ Hamilton College, Clinton, NY, USA

^3^ Center for Algorithmic Biotechnology, Saint Petersburg State University and Algorithmic Biology Laboratory, Saint Petersburg Academic University, Russia

^4^ Institute of Biomolecular Chemistry, National Research Council, Pozzuoli, Italy

The unique organisms living in the world’s oceans are an inspiring source of new pharmaceutical leads. To date, some 13 drugs of marine derivation or inspiration have reached the clinic in the US, Europe or Asia, and many more are on the horizon. Our laboratory has focused on the bioactive metabolites available from marine cyanobacteria and algae, both of which are extraordinarily rich in structurally diverse natural products. Moreover, we are exploring the integrated use of several different technologies, such as genome-based information, mass spectrometry-based Molecular Networks, and synthetic medicinal chemistry, to innovatively discover and develop new drug leads from these marine organisms. For example, a Curaçao collection of a tuft-forming cyanobacterium, *Symploca* sp., possessed an extract that was highly cytotoxic to several different cancer cell lines. A combination of a bioassay- and NMR-guided isolation process yielded two compounds, named carmaphycin A and B, which were responsible for the potent activity. We are exploring their utility as potential anticancer agents through a variety of orthogonal approaches which include synthetic organic chemistry, genome sequencing, and metabolomics. Another recent project involves a new approach that integrates genomic and metabolomic information to identify structurally novel and potentially bioactive metabolites. This latter work identified a series of novel chlorinated acyl amides that have potent binding properties to cannabinoid receptors. By integrating several different and contemporary approaches, new vistas in the natural products sciences are being revealed.

### ***Sustainable Oceans—Our Treasure in the Past and in the Future: Case Study of Sponges*** 

**Werner E.G. Müller and Xiaohong Wang**

ERC Advanced Investigator Grant Research Group, Institute for Physiological Chemistry, University Medical Center of the Johannes Gutenberg University Mainz, Duesbergweg 6, D-55128 Mainz, Germany 

In the last decade the phylogenetically oldest metazoan phylum, the Porifera (sponges) gained special interest. Mainly due to the introduction of molecular biological techniques solid evidence was elaborated which indicated that this phylum provides a cornucopia of new information which allows a grasping for the understanding of the dynamics of evolutionary processes occurring during the Earth period of Ediacara until today. Furthermore, the species of this phylum are rich and valuable sources for bioprospecting, the translation of life-science discoveries into practical products or processes for the benefit of the society. Bioprospecting: The field of bioprospecting of Porifera may be of tremendous potential benefit for humans from the applied point of view. Taking into account that the chemical diversity of the natural bioactive compounds obtained from the marine biota is much higher than the one of those compounds, synthesized in standard combinatorial chemistry approaches, and also that natural compounds display an impressively high selectivity, the high value of the secondary metabolites from natural resources in general and from sponges in particular can only be roughly imagined. Until now, a bioactive compound from sponges has been applied in clinics only in one case: arabinofuranosyladenine (ara-A) as antiviral drug; ara-A is a derivative of a lead structure isolated from a sponge. The future—evochemistry: Thanks to the progress initiated by the pressure of the society for a sustainable use of natural resources for human benefit, the exploitation of natural biodiversity became possible through the application of the techniques of molecular biology and modern cell biology [1–3]. Novel directions: Biomaterials. There is an increasing need for novel materials to be used as scaffolds in biomaterials in general and in tissue engineering (bone and cartilage) in particular. Siliceous sponges are unique in their ability to synthesize their silica skeleton enzymatically. The responsible enzymes, the silicateins which have been isolated from demosponges, polymerize alkoxide substrates to silica. Silica is an important component of materials such as bioactive glasses and composites based on glasses, ceramics and (organic) polymers. New strategies for the structure-directed synthesis of amorphous silica (biosilica) can now be envisaged [4–6]. Conclusion: It is fortunate that, according to the fossil records, the phylogenetic oldest metazoan phylum, the Porifera did not become extinct during the last 800 million years. Considerable impact in biotechnology can be expected from studies on the recombinant preparation of bioactive, low-molecular weight compounds and of the development of new biomaterials [biosilica] from marine sources.


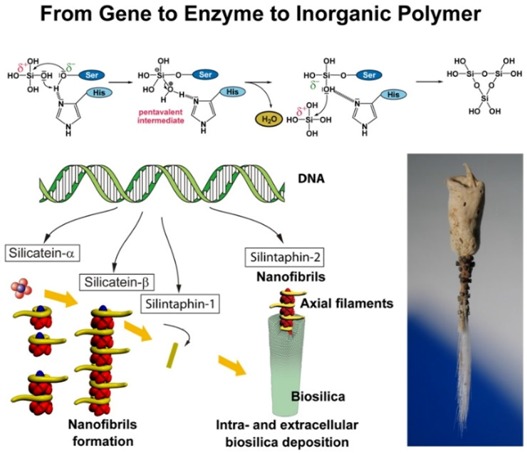


#### **Acknowledgments** 

W.E.G. M. is a holder of an ERC Advanced Investigator Grant (no. 268476 BIOSILICA). This work was supported by grants from the European Commission (Grant no. 311848 “BlueGenics” and Grant no. 286059 “CoreShell”), as well as the European Commission/EUREKA (EUROSTARS Grant no. 4289 “SILIBACTS”).

#### **References** 

1. Müller, W.E.G.; Wang, X.H.; Proksch, P.; Perry, C.C.; Osinga, R.; Gardères, J.; Schröder, H.C. Principles of biofouling protection in marine sponges: A model for the design of novel biomimetic and bio-inspired coatings in the marine environment? *Mar. Biotechnol.*
**2013**, *15*, 375–398.
2. Natalio, F.; Corrales, T.P.; Panthöfer, M.; Schollmeyer, D.; Müller, W.E.G.; Kappl, M.; Butt, H.J.; Tremel, W. Flexible minerals: Self-assembled calcite spicules with extreme bending strength. *Science*
**2013**, *339*, 1298–1302.
3. Wang, X.H.; Schröder, H.C.; Müller, W.E.G. Enzyme-based biosilica and biocalcite: Biomaterials for the future in regenerative medicine. *Trends Biotechnol.*
**2014**, *32*, 441–447.
4. Wang, X.H.; Schröder. H.C.; Wang, K.; Kaandorp, J.A.; Müller, W.E.G. Genetic, biological and structural hierarchies during sponge spicule formation: From soft sol-gels to solid 3D silica composite structures. *Soft Matter*
**2012**, *8*, 9501–9518.
5. Schröder, H.C.; Wang, X.H.; Tremel, W.; Ushijima, H.; Müller, W.E.G. Biofabrication of biosilica-glass by living organisms. *Nat. Prod. Rep.*
**2008**, *25*, 455–474.
6. Müller, W.E.G.; Schröder, H.C.; Schlossmacher, U.; Grebenjuk, V.A.; Ushijima, H.; Wang, X.H. Induction of carbonic anhydrase in SaOS-2 cells, exposed to bicarbonate and consequences for calcium phosphate crystal formation. *Biomaterials*
**2013**, *34*, 8671–8680.

### ***In Search of Algal Metabolites with Biomedical-Biotechnological Potential from the East Mediterranean*** 

**Vassilios Roussis**

Department of Pharmacognosy and Chemistry of Natural Products, Faculty of Pharmacy, University of Athens, Greece

The biodiversity of the Mediterranean ecosystem hosts an immense number of indigenous species, as well as organisms that have migrated from the Atlantic Ocean, the Red and the Black Seas. Although the Mediterranean basin occupies only 0.8% of the world’s ocean area, it accounts for 7.5% of all described marine species. Many of these organisms have been proven a prolific source of interesting metabolites with a broad spectrum of bioactivities. As part of our studies on the chemical composition and biological activity of marine organisms, our group has investigated a significant number of algal species found along the Greek coastline. In search of a fast and reliable screening tool for the chemical profiling of *Laurencia* algal extracts and the detection of new secondary metabolites, we have developed a high throughput fingerprinting methodology based on the complementary application of LC-HRMS-DAD and 2D NMR. The preliminary results of this study point out the potential for the direct screening of crude algal extracts in order to detect new compounds, as well as to trace biomarkers and/or monitor the presence of targeted metabolites. Brominated diterpenes isolated from the red alga *Sphaerococcus coronopifolius*, as well as a panel of synthetic analogues have been evaluated for their settlement inhibitory potential on the cyprids of *Balanus amphitrite* resulting in the detection of compounds that exhibit high levels of activity without toxic effects on non-target organisms. The active compounds were loaded in copper oxide nanospheres and were incorporated in CNT-enriched self-healing/self-polishing anticorrosive/antifouling marine paints. Novel fibrous biocomposites comprising ulvan, a sulfated polysaccharide extracted from the green seaweed *Ulva rigida*, and a number of copolymers were successfully prepared using the electrospinning technique. Such nanofibrous matrices represent potentially useful materials in the biomedical sector as tissue engineering scaffolds, wound dressings, or drug delivery systems.

## Session 2. Invited Speakers Abstracts

### **Chemical Ecology** 

#### **Future Exploitation of Marine Natural Products: A Marine Ecological Perspective** 

**Joanne S Porter**

School of Life Sciences, Heriot Watt University, Edinburgh, UK

The downsizing of natural product chemistry by big Pharma, and the associated emphasis of combinatorial chemistry approaches to develop new molecules have not borne fruit to novel pharmaceuticals to the extent originally envisaged. In recent years, interest in natural products has increased particularly in the marine environment as many of the terrestrial niches have been exhausted or rendered inefficient by the rediscovery problem. In order to maximize the chances of developing novel bioactives for a variety of applications in the future, it is critical that innovative approaches are considered. For many of the marine phyla that have been investigated there is some understanding of the roles that natural products play. However, the potential functions of small molecules in other phyla are less well understood. A relatively under-investigated phylum is the Bryozoa; studies on the more abundant, particularly foliose species, have shown that bryozoans have good potential as a source of novel bioactive compounds. However, a large number of bryozoans are surface-encrusting species and are difficult to study. In these cases, where there is little biomass available to analyze, an alternative approach is to investigate the ecological relationships between the bryozoan and other organisms. Specialist predators (for example, sea slugs) that live in close association with marine invertebrates may concentrate or modify bryozoan-derived molecules. Microbial symbionts are also a potential source of novel compounds in bryozoa, the best example being the production of bryostatin by the uncultured *Endobugula sertula* bacterium associated with larvae of *Bugula neritina*. Isolation of the primary producers and cultivation of these microbes would be expected to overcome some of the issues of availability and scaling typically encountered in biotechnology development pipelines. In the future the development of close working relationships between chemists and marine ecologists to develop innovative approaches is likely to lead to a deeper understanding of the functional role of compounds in the marine environment. A more complete knowledge of ecological relationships will guide investigation of related organisms that mirror growth strategies or share habitats, ultimately leading to the identification of a wider range of novel molecules.

### **Deep Sea and Polar** 

#### **Research Living in the Cold: Chemical Ecology in Antarctica** 

**Conxita Avila**

Universitat de Barcelona, Facultat de Biologia, Department Animal Biology (Invertebrates), Catalonia, Spain

During many years it was believed that very few animals would live in higher latitudes (*i.e.*, low biodiversity) and that they would have low levels of chemical defenses (*i.e.*, low chemical diversity). However, this has been proven not to be true, with many evidences accumulating on the knowledge of both polar (mostly Antarctic) biodiversity and chemical ecology. The extreme and unique marine environments surrounding Antarctica, along with their evolutionary history and the abundant unusual interactions taking place in its benthic communities are a fantastic natural laboratory for finding new natural products. In general, many bioactive compounds have been described from the oceans of our planet, being probably essential for species’ survival. Surprisingly, only a small fraction of these molecules has been tested for ecological significance, and even less if we look at compounds from polar organisms. Some Antarctic animals are now known to be prolific producers of chemicals, with wide potential applications. But in Antarctica, as in other areas of the planet that are difficult to reach (*i.e.*, deep sea), a huge number of organisms remain to be studied yet, both at biological and at chemical levels. I will review here some of the known compounds found in Antarctic animals as well as their role in the communities where the animals live.

### **Marine Toxins and Bioassays** 

#### **Fishing for Neuroactive Small Molecules: Zebrafish as a Platform for Marine Biodiscovery** 

**Camilla Esguerra**

Chemical Neuroscience, Biotechnology Centre of Oslo, University of Oslo, Oslo, Norway

Emerging challenges within the current drug discovery paradigm are prompting renewed interest in secondary metabolites as an attractive source of novel, structurally diverse small molecules that have been evolutionarily “pre-selected” for bioactivity. With the recent validation of zebrafish as a biomedically relevant model for functional genomics and *in vivo *drug discovery, the zebrafish bioassay-guided identification of natural products is an attractive strategy to generate new lead compounds in a number of indication areas. We have recently developed a number of *in vivo*, microgram-scale, high-throughput bioassays based on zebrafish embryos and larvae for the systematic identification and pharmacological characterization of bioactive natural products. Zebrafish offer the ability to rapidly evaluate—at a very early stage in the drug discovery process—not only the therapeutic potential of natural products, but also their potential hepato-, cardio-, and neurotoxicities. Due to the requirement for only microgram quantities of compounds to be tested, *in vivo* assays based on zebrafish are useful not only for bioassay-guided isolation, but also for the subsequent derivatization of bioactive natural products prioritized for further development as drug discovery leads. Within the EU marine biodiscovery project PharmaSea (www.pharma-sea.eu), for example, zebrafish-based CNS assays have been used to screen thousands of crude extracts of marine microorganisms, and are being used for the bioactivity-guided isolation of neuroactive secondary metabolites from these extracts. These findings underscore the potential utility of zebrafish disease and toxicity models for marine biodiscovery.

### **Dereplication Metabolomics, and Rational Approaches to Bioprospecting** 

#### **Dereplication and Metabolomics in Microbial Bioprospecting** 

**Kristian Fog Nielsen**

Technical University of Denmark, Department for Systems Biology, Lyngby, Denmark

For discovery of novel compounds, fast dereplication of known compounds is vital for prioritization of which strains and extracts to explore further. Usually UHPLC with high-resolution mass spectrometry (HRMS) is used as the number of candidate returned from a database search is reduced 5–20 fold compared to nominal mass MS. For exploring the major peaks in an UHPLC-HRMS data-file from a relative clean extract, a simple peak picking algorithm followed by adduct pattern interpretation is fast. However not all natural products ionize well and if combined with many strongly ionizing compounds, media components, contaminants from filters plastic-ware *etc.* the simple task get extremely complicated and time-consuming. For this 3 approaches are used:

(i) A metabolomics approach where 4-6 replicates per condition, along with 4-6 replicates of media blanks are analyzed in a single sequence. However a single data-file may contain 10000-20000 features (ion clusters) including many random false features to be discarded using the replicates.
(ii) A targeted approach using lists (up to many thousands) of elemental compositions of possible candidates (aggressive dereplication), where isotopic patterns, known adducts, retention time windows can be exploited along with reference standards and known blanks. These can then be combined with a peak picking approach for novel compounds.
(iii) MS/HRMS library approach where known and tentatively identified compounds are identified by auto-MS/MS (data dependent MS/MS), and if combined ion-trap MS^n^ can be used for identification of known substructures in larger unknown structures.

For dereplication fungal extracts we have found a combination of II and III to suit our applications as we can employee taxonomic knowledge, 25 years of HPLC-DAD data, and bioinformatics. Upcoming technologies in the field include 2D LC and ion-mobility.

### **Advances in Isolation and Structure Elucidation** 

#### **Modern NMR Methods for Structure Elucidation of Natural Products** 

**Teodar Parella**

Servei de Ressonància Magnètica Nuclear, Universitat Autònoma de Barcelona, Catalonia, Spain

High-resolution NMR spectroscopy is a fundamental tool for characterizing the structure and dynamics of natural products. Here, a general overview of novel NMR techniques will be presented and exemplified on several compounds. New versions of the HSQMBC experiment (LR-HSQMBC, HSQMBC-TOCSY, edited-HSQMBC, *etc.*) will be shown as complementary tools of the classical experiments [1–3]. On the other hand, the advantages to collapse typical proton multiplet resonances to singlet lines will be highlighted, allowing an unprecedented increase of signal resolution and an easy chemical shift determination and differentiation, even in the analysis of highly overlapped regions and complex mixtures [4]. Application of these pure-shift techniques to resolve typical chemical problems and to determine NMR parameters will be discussed. As an example, an improved homodecoupled variant of the ADEQUATE experiment has been useful to resolve long-standing structural questions associated with the structure of cryptospirolepine, a complex cryptolepis alkaloid [5].

##### ***References*** 

1. Williamson, R.T.; Buevich, A.; Martin G.E.; Parella, T. LR-HSQMBC: A sensitivity NMR technique to probe very long-range heteronuclear coupling pathways. *J. Org. Chem.*
**2014**, *79*, 3887–3894.
2. Saurí, J.; Marcó, N.; Williamson, R.T.; Martin G.E.; Parella, T. Extending long-range heteronuclear NMR connectivities by HSQMBC-TOCSY and HSQMBC-COSY experiments. *J. Magn. Reson.*
**2015**, *258*, 25–32.
3. Saurí, J.; Sistaré, E.; Williamson, R.T.; Martin, G.E.; Parella, T. Implementing multiplicity editing in selective HSQMBC experiments. *J. Magn. Reson. *
**2015**, *252*, 170–175.
4. Castañar, L.; Parella, T. Broadband ^1^H homodecoupled NMR experiments: Recent developments, methods and applications.* Magn. Reson. Chem.*
**2015**, *53*, doi:10.1002/mrc.4238.
5. Saurí, J.; Bermel, W.; Buevich, A.V.; Sherer, E.C.; Joyce, L.A.; Sharaf, M.H.M.; Schiff, P.L.; Parella, T.; Williamson, R.T.; Martin, G.E. Homodecoupled 1,1- and 1,*n*-ADEQUATE: Pivotal NMR Experiments for the structure revision of Cryptospirolepine. *Angew. Chem. Int. Ed.*
**2015**, *54*, 10160–10164.

### **Industrial Biotechnology, Polymers and Biomolecules** 

#### **Novel Enzymes and Organisms for Processing Polysaccharides from Brown Algae** 

**Gudmundur Oli Hreggvidsson**

Matis ohf. Vinlandsleid & Faculty of Life and Environmental Studies, University of Iceland, Iceland

Brown algae are abundant in coastal areas of the North Atlantic. Their growth rates and productivities far exceed those of terrestrial plants and they accumulate high levels of carbohydrates (up to 60%). The abundance and the high carbohydrate content make macroalgae a potential highly valuable feedstock for bioconversion to platform-, specialty-chemicals, and advanced energy carriers. The possible product range from macroalgae far surpasses other biomass of comparable bulk and ease of cultivation. Brown algae are an under-utilized resource, but of special significance for Northern Europe with abundant growth along long shorelines and the access to vast sea areas for potential off-shore cultivation. The polysaccharide components of macroalgae are of potential high biotechnological value, but because of structural complexity and “unconventional” and heterogeneous sugar composition, sulfatation and other modifications they are also challenging as a biorefinery feedstock. A number of processing problems need to be resolved in order to make a marine biorefinery an economically feasible option. Matis is developing enzymes and organisms for use in emerging macroalgal biorefineries with a special focus on bioconversion of Brown algae polysaccharides. This includes enzymes for degrading the polysaccharides, alginate and laminarin, to fermentable mono sugars, enzymes for use as aids in fractionation of macroalgal biomass and enzymes for modification or conversion of polysaccharides to added value derivatives. Matis is, furthermore, involved in developing robust thermophilic microorganism for use as cell factories in macroalgal biorefineries. In this work Matis looks somewhat beyond current markets towards sustainable bulk production of platform chemicals, energy carries and higher value products for specialized markets. Some examples will be given in the talk on our recent research in this field.

### **Organic Synthesis** 

#### **Bioactive Natural Product Synthesis: From Structure Elucidation to Drug Discovery Platforms** 

**Gordon Florence**

School of Chemistry and Biomedical Science Research Complex, University of St Andrews, North Haugh, St Andrews, UK

Nature provides an armada of structurally diverse secondary metabolites with unique and often unexplored biological modes of action. Combined with their molecular architectures natural products continue to provide the inspiration to develop practical synthetic routes and methods not only to provide confirmation of their complete structure but to serve as blueprints for drug discovery This talk will discuss our recent endeavours encompassing approaches to oxazole containing natural products and the development of natural product inspired inhibitors of T. brucei the parasite responsible for African sleeping sickness.

### **Biosynthesis of Marine Natural Products in Microbes** 

#### **Sailing the Uncharted Seas of Marine Phytoplankton** 

**Angelo Fontana, Carmen Gallo, Adele Cutignano, Genoveffa Nuzzo, Angela Sardo, Emiliano Manzo and Giuliana d’Ippolito**

CNR—Institute of Biomolecular Chemistry, Via Campi Flegrei 34, Pozzuoli, 80078 Naples, Italy

Phytoplankton is an essential biological component of marine environment. The group is composed of photosynthetic microorganisms that are central to ecological and biogeochemical functions. As primary producers, these single-celled organisms provide nourishment to many marine species and are also major drivers in the cycling of elements. Carbon uptake by phytoplankton, and its export as organic matter to the ocean interior (a mechanism known as the “biological pump”) facilitates the diffusive drawdown of atmospheric CO_2_. There are two main types of the larger phytoplankton species: Diatoms and Dinoflagellates. Diatoms are the most productive phytoplankton group in the world oceans accounting for about 40 percent of the marine primary production. These unicellular photosynthetic organisms form the basis of food webs in coastal and upwelling systems, support important fisheries and have a major role in silica cycling. On the other hand, in addition to be important marine primary producers and grazers, dinoflagellates are the major causative agents of harmful algal blooms since many species can produce various toxins that pose a health danger to human populations and negatively affect economic activities. While the impact of these organisms is very clear, the most fundamental questions about their physiology and biochemistry remain to be solved. Thus, despite the central role in marine habitats and the fascinating chemistry of their secondary metabolites, our knowledge of the biosynthetic process in these protists is still at an early stage, providing unique opportunities to make new and exciting discoveries. Starting from the study of the chemo-ecological interactions between diatoms and their main grazers, the copepods, our scientific interest for phytoplankton has over time expanded to embrace general aspects concerning potential application and metabolism of these organisms, including regulation of physiological and ecological mediators. This contribution will discuss these topics and recent results on biosynthesis of secondary metabolites in marine diatoms and dinoflagellates.

### **Marine Microbes** 

#### **Genomic and Metagenomic Approaches to Mine Marine Sponge Associated Microorganisms for Novel Bioactive Compounds with Potential Biopharmaceutical Applications** 

**Stephen Jackson ^1^, Stefano Romano ^1,2^, Lisa Crossman ^3^, Claire Adams ^2^, Fergal O’Gara ^1,2,4^ and Alan Dobson ^1^**

^1^ Marine Biotechnology Centre, School of Microbiology, University College Cork, Cork, Ireland

^2^ BIOMERIT Research Centre, School of Microbiology, University College Cork–National University of Ireland, Cork, Ireland

^3^ School of Biological Sciences, University of East Anglia, Norwich, UK

^4^ School of Biomedical Sciences, Curtin University, Perth, WA 6845, Australia

Marine sponges are well established as a source of bioactive natural products, many of which originate from the resident microorganisms rather than from the sponge itself. Diverse communities of sponge microorganisms, many of which appear to be symbionts can comprise up to 40% of the sponge biomass. Metagenomic based approaches have been employed to study the microbial ecology of both shallow water sponges such as Haliclona simulans and Axinella dissimilis and deep sea sponges such as Inflatella pellicula and *Stelletta normanii* sampled from depths ranging from ~750 m to 2900 m to determine whether the sponge associated microbiota contain bacterial genera that are likely producers of bioactive compounds. Using culture based approaches and screening for bioactivity against clinical pathogens such as *Salmonella enterica* serotype Typhimurium, methicillin-resistant *Staphylococcus aureus* (MRSA), and *Clostridium difficile*; three major groups of microorganisms with antibacterial activity, spore forming *Bacillus*, *Streptomyces* and *Pseudovibrio* species have been identified. Our strategy has involved the sequencing and annotation of the genomes of these bioactive strains, and the subsequent analyses for gene clusters encoding potential novel bioactive compounds using the web server AntiSMASH. Using this approach we have identified Subtilomycin a novel lantibiotic produced by *Bacillus* strain MMA7, while genome mining of 12 *Pseudovibrio* species has resulted in the identification of tropodithietic acid (TDA) and other potentially novel small molecules. Genome sequencing of 15 *Streptomyces* strains, a number of which are from deep sea sediments; indicate the presence of multiple secondary metabolism gene clusters which appear to be involved in the synthesis of polyketides, nonribosomal petides, lantipeptides, terpenes and bacteriocins.

### **Marine Policy** 

#### **Regulation of Access to Genetic Resources and Related Benefit-Sharing at the International and European Level—Learning the ABC of ABS** 

**Thomas Greiber**

Ocean Governance, Institute for Advanced Sustainability Studies e.V. (IASS), Potsdam, Germany

Innovation based on genetic resources relies to some extent on having physical access to genetic material. Such access, as well as related benefit-sharing is regulated by a number of international, regional and national legal instruments, some of which will be introduced to the audience in this presentation. The presentation will provide a quick introduction to the concept of access to genetic resources and fair and equitable benefit-sharing (ABS) developed under the Convention on Biological Diversity (CBD). In short, according to the ABS concept, States hosting/providing genetic resources shall facilitate access to these resources while users shall share in a fair and equitable manner the benefits arising from the access to and use of the resources. The presentation will then look in more detail at the Nagoya Protocol on Access to Genetic Resources and the Fair and Equitable Sharing of Benefits Arising from their Utilization. Being a supplementary agreement to the CBD the objective of the Nagoya Protocol is to set an international, legally binding framework to promote the implementation of the ABS concept in the future. The Protocol aims at establishing rules determining

- how researchers and companies who utilize genetic resources and/or traditional knowledge associated with genetic resources will obtain access to them (**A**ccess),
- how benefits arising from such utilization will be shared (**B**enefit-sharing), and
- which measures will need to be taken to ensure that users respect the ABS measures of the country providing the genetic resources and associated traditional knowledge (**C**ompliance).

Furthermore, the presentation will give an overview of the new EU ABS Regulation implementing the Nagoya Protocol for the European Union. This Regulation ((EU) No 511/2014) applies from the date the Nagoya Protocol itself entered into force for the Union, *i.e.*, on 12 October 2014 (with some provisions of the Regulation only becoming applicable one year after that).

#### **Access and Exploitation of Biological Biodiversity: New Challenges and Their Impact on Natural Products Research** 

**Olga Genilloud**

Fundación MEDINA, Health Sciences Technoogy Park, Granada, Spain

The implementation of CBD in response to the need of a sustainable use of biological diversity, with fair and equitable sharing of future exploitation benefits, defined a new paradigm in the way industry addressed international collaboration models with third countries. After 20 years of efforts to clearly manage the access and benefit sharing to these genetic resources, and the transfer of relevant technology, we have seen that wrong expectations about the potential economic returns, emerging protective legislation in some countries, and the absence of clear authorities to grant access, determined the introduction of voluntary guidelines to assist the efficient management of exploitation of ABS that were reinforced more recently with the Nagoya protocol. Whereas this protocol aims at providing a legal framework for providers and users of genetic resources, including a proposed infrastructure to track materials and agreements, its conceptual and practical implementation is becoming really challenging in many countries and has set up the alarm for users both in the academic and industry communities. The implications of this new policy and legislation on natural product research will be discussed from the perspective of both communities. These uncertainties derived from the poor developed models for the country-to-country definitive implementation represent more than ever clear barriers to the exploitation of these genetic resources. These problems should be addressed jointly by the administration and all stackeholders in the field to develop a long term sustainable model fostering research in natural products.

## **Session 3. Abstracts Selected for Oral Presentations** 

### **Chemical Ecology** 

#### **Secondary Metabolites of *Phyllodesmium longicirrum* and Their Role in Defense and Evolution** 

**Alexander Bogdanov ^1^, Cora Hertzer ^1,2^, Stefan Kehraus ^1^, Samuel Nietzer ^3^, Sven Rohde ^3^, Peter J. Schupp ^3^, Heike Wägele ^2^ and Gabriele M. König ^3^**

^1^ University of Bonn, Pharmaceutical Biology, Bonn, Germany

^2^ Zoologisches Forschungsmuseum Alexander König, ZFMK, Bonn, Germany

^3^ University of Oldenburg, ICBM Terramare, Wilhelmshaven, Germany

The aim of the proposed project is to shed light on the evolution of the defensive system of an opisthobranch group, the Cladobranchia. Within Cladobranchia, the taxon Aeolidoidea is known to incorporate cnidocysts from their prey for its own defense. This has been suggested as a major driving force for speciation and an explanation for the success of the Aeolidoidea. Another defensive strategy in shell-less opisthobranchs is the incorporation of secondary metabolites from prey organisms. Our project focuses on natural products in species belonging to the genus *Phyllodesmium* (Cladobranchia, Aeolidoidea) in correlation with their food. Members of the genus *Phyllodesmium* feed exclusively on different alcyonacean corals, which are a rich source of terpenoid secondary metabolites. *P. longicirrum* yielded several new secondary metabolites (**1**–**5)**, besides already known ones (e.g., sarcophytonin B, isosarcophytoxide and polyhydroxylated steroids). The new compounds belong to the class of cembrane diterpenes (**1**), secogorgosterols (**2**), more unusual tetracyclic and pentacyclic diterpenes (**3**–**5**). The defensive role of the isolated compounds could be demonstrated by feeding deterrence assays using the tropical pufferfish *Canthigaster solandri* as predator. VLC fractions of the crude extract were deterrent at levels below natural concentrations. Compound **3** exhibited significant deterrence at 0.5% of dry mass and thus is suggested to play an important role in chemical defense against predation. Our findings will finally be mapped onto a phylogenetic reconstruction of the Cladobranchia to understand evolution of different pathways of defensive strategies.

##### ***Acknowledgments*** 

DFG Wa 618/10-1 and KO 902/8-1.

#### **Natural Inducers for Larval Metamorphosis in Scleractinian Corals** 

**Peter J. Schupp, Mareen Möller, Samuel Nietzer and Makoto Kitamura**

Institute of Chemistry and Biology of the marine Environment, Oldenburg, Germany

Many benthic marine invertebrates, including corals, disperse as plankton before settlement and metamorphosis. Finding a suitable habitat is crucial for sessile marine invertebrates. The ability of larvae to detect habitat-specific cues has been recognized in a range of phyla, but until recently, only a few studies identified the chemical structure of compounds involved in larval settlement and metamorphosis. Biofilms on Crustose Coralline Algae are a known inducer for settlement in scleractinian corals but only little is known about specific bacteria and chemical compounds involved in metamorphosis and settlement of coral larvae. Here we present insights into the role of bacteria during the coral larvae settlement, using larvae of the brooding coral *Leptastrea purpurea* as a model. Experiments testing the involvement of CCA biofilms, as well as biofilms from inert surfaces (e.g., tiles, bleached CCA), revealed that biofilms on *Hydrolithion* and biofilms on certain inert surfaces more than three weeks old repeatedly induced settlement in *L. purpurea* and *Acropora* larvae. Continued studies lead also to the isolation of two settlement-inducing bacteria (both *Pseudoalteromonas *sp.). Using bioguided fractionation of crude extracts from inducing bacteria we could show that certain bacteria produce chemical cues, which induce the settlement process. Two settlement and metamorphosis inducing compounds have so far been isolated. Further assays also identified the presence of a toxic compound in the bacterial extracts. Their structure elucidation is currently under way.

#### **Chemical Interactions between the Toxic Microalgae *Ostreopsis cf. ovata* and Mediterranean Benthic Diatoms** 

**Eva Ternon ^1^, Sophie Marro ^2^, Rodolphe Lemée ^2^ and Olivier Thomas ^1^**

^1^ Nice Institute of Chemistry—PCRE UMR7272 CNRS, University of Nice Sophia Antipolis, Nice, France

^2^ Sorbonne Universités, Université Pierre et Marie-Curie Paris 6, CNRS, Laboratoire d’Océanographie de Villefranche, Villefranche-sur-mer, France

Several species of microalgae own a well-developed specialized metabolome yielding to the production of toxic compounds. When highly concentrated and quickly multiplying, these toxic microalgae are likely to induce negative environmental or toxicological effects, by forming Harmful Algal Blooms (HABs). During the past decade, a toxic benthic dinoflagellate belonging to the genus *Ostreopsis* has bloomed repetitively along the Mediterranean coastline, leading to toxic outbreaks on humans. Their efflorescence has become a real concern in regard to public health and several toxicological studies on the major toxins (ovatoxins) have been carried out. On the other hand, their effects on the Mediterranean benthic community have not been assessed yet. In this study we propose to investigate the chemical mediation involved between Ostreopsis cf. ovata and a Mediterranean benthic diatoms. Co-cultures without contact were set up using dialyze bags, and the chemical content of both cells and culture media was analyzed. Since the specialized metabolism of *O. *cf. *ovata* remains scarcely known, an un-targeted metabolomics approach was used to compare the chemical signals present in cultures thanks to a high performance UHPLC-QTof. Chemical cues found to be involved in the interactions were submitted to a MS-MS approach and compared to natural products databases in order to propose a potential identity.

#### **Irish *Osmundea* spp.: Food or Shelter for *Aplysia* sp.?** 

**Sylvia Soldatou ^1^, Ryan Young ^1,2^, Candice Bromley ^1^, Svenja Heesch ^3^ and Bill Baker ^1,2^**

^1^ National University of Ireland, Galway, Galway, Ireland

^2^ Department of Chemistry and Center for Drug Discovery and Innovation, University of South Florida, Tampa, FL, USA 

^3^ Irish Seaweed Research Group, Ryan Institute for Environmental, Marine and Energy Research, National University of Ireland, Galway, Galway, Ireland

The Irish coastline is approximately 7500 km long representing one of the most biodiverse and rich-species habitats in Europe. With only few studies conducted in the North East Atlantic region, Irish waters can be a great source of new and unexplored chemical diversity. Four different *Osmundea* sp., red alagae commonly found in intertidal zone, have been described from Irish waters. *Aplysia* sp., is a sea hare which has been found to be associated with *Osmundea* algae. This project is focusing on the isolation and characterisation of secondary metabolites from *Osmundea* spp. and* Aplysia* sp. samples collected from the shore of Western Ireland in county Galway. The ultimate aim is to compare the chemistry produced by these two marine organisms and determine whether the sea hares are sequestering the compounds from the algae or they are using the *Osmundea* spp. as a shelter from predators and strong water currents. Thus half the *Aplysia* sp. collected were allowed to fast prior to analysis affording an opportunity to sample the chemistry contained within the sea hares rather than that contained within the digestive tract. The algal and animal samples were extracted separately in organic solvents followed by purification and isolation of secondary metabolites by means of Medium Pressure Liquid Chromatography (MPLC) and High Performance Liquid Chromatography (HPLC). Comparisons between the algal and sea hare extracts were carried out through metabolomics analysis using Liquid Chromatography-Mass Spectrometry (LC-MS) and Gas Chromatography-Mass Spectroscopy (GC-MS). Moreover, the structures of pure metabolites were elucidated by means of 1D and 2D Nuclear Magnetic Resonance (NMR) spectroscopy.

#### **Dual Induction of Microbial Secondary Metabolites by Fungal/Bacterial Co-Cultivation** 

**Mostafa Rateb ^1,2^, Jennifer Wakefield ^1^, Rainer Ebel ^1^ and Marcel Jaspars ^1^**

^1^ Marine Biodiscovery Centre, Department of Chemistry, University of Aberdeen, Aberdeen, UK

^2^ Pharmacognosy Department, Faculty of Pharmacy, Beni-Suef University, Beni-Suef, Egypt

Marine-derived microorganisms are promising sources of new bioactive metabolites. However, the frequent re-discovery of known compounds is a major problem. Many biosynthetic genes are not expressed *in vitro* thus limiting the chemical diversity of microbial compounds that can be obtained through fermentation. On the other hand, the co-cultivation (also called mixed fermentation) of two or more different microorganisms helps to mimic complex microbial natural communities. The competition during co-cultivation in most cases lead to an enhanced production of constitutively present compounds, or to an induction of cryptic compounds that are not detected in axenic cultures of the producing strain. Herein, we report the induction of newly detected bacterial and fungal metabolites by the mixed fermentation of the marine-derived fungal isolate MR2012 and hyper-arid desert bacterial isolates.

### **Deep Sea and Polar Research** 

#### **Isolation and Characterization of Bioactive Deep-Sea Marine Fungi** 

**Stephen A Jackson ^1^, Erik Borchert ^1^, Jonathan Kennedy ^1^, Fergal O’Gara^1,2^ and Alan D.W. Dobson ^1,2^**

^1^ School of Microbiology, University College Cork, Cork, Ireland

^2^ BIOMERIT Research Centre, University College Cork, Cork, Ireland

In the search for novel marine natural products with antimicrobial activities, the deep-sea remains an as yet largely under-sampled environment. As terrestrial fungi are well known producers of antimicrobial agents, marine fungi may be a promising source of novel compounds with potential to combat the increasing threat of antimicrobial resistances amongst human pathogenic bacteria. We have isolated fungi from deep-sea sediment sampled from a depth of 1168 m and from a marine sponge (*Stelletta normani*) sampled from a depth of 751 m. Samples were obtained from the Atlantic Ocean off the west coast of Ireland. Nineteen isolates (14 from the sponge and 5 from sediment) were taxonomically characterized by PCR amplification targeting 18S rRNA gene fragments using primers EukA and EukB [1] followed by sequencing, BLAST analyses and phylogenetic tree-building. All sediment isolates recruited to the genus *Cladosporium* and are closely related to sequences derived from deep-sea or hypersaline environments. Sponge derived isolates are closely related to the genera Ascomycete, Cadophora and Geomyces. Phylogenetic analyses suggest these isolates may be true marine fungi. All fungal isolates were tested for antibacterial activities against clinically relevant Gram positive and Gram negative test strains. Although little if any antibacterial activity was observed from the sediment isolates, 7 sponge isolates showed activity against one or more test strains. Three isolates in particular (TS3, TS12 & TS13) inhibited all test strains in the agar overlay assay. The secondary metabolite potential of these strains was investigated by PCR through targeting of potential Polyketide synthase (PKS) and Non Ribosomal peptide synthase (NRPS) genes in these fungal genomes.

##### ***Reference*** 

1. Medlin, L.; Elwood, H.J.; Stickel, S.; Sogin, M.L. The characterization of enzymatically amplified eukaryotic 16S-like rRNA-coding regions. *Gene* **1988**, *71*, 491–499.

#### **Investigation into the Bioactive Metabolites of Deep Sea Sponge Associated Fungi** 

**Candice Bromley ^1^, Ryan Young ^1,2^, Stephen Jackson ^3^, Thomas Sutton ^3^, Alan Dobson ^3^ and Bill Baker ^1,2^**

^1^ National University of Ireland Galway, Galway, Ireland

^2^ Center for Drug Discovery and Inovation, University of South Florida, Tampa, FL, USA

^3^ Marine Biotechnology Centre, School of Microbiology, University College Cork, National University of Ireland Cork, Cork, Ireland

Marine associated fungi, especially those isolated from extreme environments, have been found to produce diverse bioactive secondary metabolites. The ability to manipulate the fungal genome to amplify the production of particular metabolites adds to their potential in the discovery of novel bioactive compounds. In this study the ROV Holland I was used to collect samples of the sponge *Stelletta normani* off the west coast of Ireland from a depth of 751 m. Eleven deep sea sponge associated fungi were isolated and cultured. Initial testing for bioactivities was performed using a deferred antagonism assay against gram negative bacteria such as *Escherichia coli *and *Pseudomonas aeruginosa*, as well as gram positive bacteria such as *Staphylococcus aureus* and *Bacillus subtilis*. In an attempt to increase the production of bioactive secondary metabolites epigenetic modifiers, capable of activating silenced or attenuated gene clusters in the fungi, were employed. Each of the fungal strains were exposed to two such epigenetic modifiers, namely sodium butyrate and 5-azacytidine, inhibiting histone deacetylase (HDAC) and DNA methyltransferase (DNMT) respectively. To establish the effects of epigenetic modifications on the propensity for the fungi to produce bioactive secondary metabolites the activities of the organic extracts were retested, this time covering a wider range of pathogens. In addition, comparative investigations of the fungal metabolomic profiles using LC-MS as well as NMR spectroscopy were conducted to establish any potential bioactive natural products.

#### **Antimicrobial Compounds from Antarctic Bacteria** 

**Donatella de Pascale ^1^, Pietro Tedesco ^1^, Fortunato Palma Esposito ^1^, Antonio Mondini ^1^, Glen Brodie ^2^, Renato Fani ^3^ and Marcel Jaspers ^2^**

^1^ Institute of Protein Biochemistry, CNR, Naples, Italy

^2^ University College of Aberdeen, The School of Natural and Computing Sciences, Aberdeen, UK

^3^ Department of Biology, University of Florence, Florence, Italy

The increasing alarm of multidrug resistant bacteria in the last 20 years, led scientific community to the discovery of novel source of antimicrobials compounds. The bioprospecting from marine and extreme environments has yielded a noteworthy number of novel molecules from a wide range of organisms. Antarctica is the one of the most extraordinary places on Earth and exhibits many distinctive features. It is Earth’s southernmost continent and it is the coldest, driest, and windiest place on the planet. Thus, Antarctica hides organisms, which have evolved unique characteristics to face these harsh environmental conditions. In particular, Antarctic microorganisms are known to produce novel secondary metabolites that are valuable in a range of applications. Herein, we report on the development of a six-step biodiscovery pipeline starting with the collection of environmental samples and isolation of novel bacteria, to the chemical identification of the bio-assay guided purification of compounds with antimicrobial and antibiofilm activities. Antarctic sub-sea sediments were used to isolate more 1000 bacteria. The novel isolates were subjected to primary screening to determine their bioactivity against a selected panel of human pathogens (*Staphylococcus aureus*, *Pseudomonas aeruginosa*, *Klebsiella pneumonia*, *Burkholderia cenocepacia*). Isolates, positive to the first screening, were used to produce crude extracts from microbial exhausted culture broths. A bioassay-driven purification was performed using crude extracts of the most promising isolates. LC-MS and NMR then structurally resolved the purified bioactive compounds.

#### **Marine Bacteria Isolated from Deep-Sea Hydrothermal Vents Are Valuable Sources of Glycosaminoglycan-Like Polysaccharides and Anti-Microbials** 

**Christine Delbarre-Ladrat, Laetitia Kolypczuk, Delphine Passerini, Jacqueline Ratiskol, Corinne Sinquin, Agata Zykwinska, Sylvia Colliec-Jouault and Françoise Leroi**

Ifremer, Nantes, France

The study of microbial life adapted to deep-sea hydrothermal vents conditions is a promising way of discovering new biomolecules with innovative properties and potential applications in human health. On one hand, our collection of bacteria isolated from deep-sea hydrothermal vents is screened for anti-microbial activities to fight the growing threat of broad spectrum antibiotic resistant infections. A range of Gram-positive and Gram-negative bacteria pathogens encountered in human health field, aquaculture and food spoilage constitutes the selected target bacteria. On the other hand, marine prokaryotes offer a source of safe, biocompatible, biodegradable and valuable renewable products especially carbohydrate polymers. Deep-sea marine bacteria have been shown to produce exopolysaccharides (EPS) with unusual structure and having glycosaminoglycan (GAG)-like biological activities; in particular, some of these EPS are naturally sulfated; this is very rare within the prokaryote domain. Data on three EPS-producing bacteria isolated from deep-sea habitats will be presented. These would include EPS production in bioreactors, molecular mechanisms of the polysaccharide biosynthesis, chemical and enzymatic modifications, carbohydrate active enzymes as well as some biological properties which give them high value for the biomedical field. These studies provide a better basic knowledge on the biosynthesis of bioactive polysaccharides and would provide means to engineer the molecule for improving its function.

#### **Apoptosis Mediated Anticancer Activity of *Streptomyces* sp. MCCB 248 Isolated from an Arctic Fjord, Kongsfjorden, Svalbard, on NCI-H460 Human Lung Cancer Cell Line** 

**Dhaneesha M. ^1^, Sajeevan T. P. ^1^, Krishnan K. P. ^2^ and Bright Singh I. S. ^1^**

^1^ National Centre for Aquatic Animal Health, Cochin University of Science and Technology, Kochi, Kerala, India

^2^ National Centre for Antarctic and Ocean Research, Headland Sada, Goa, India

A total of 22 actinomycetes were isolates from the sediments collected from the Kongsfjorden, Arctic fjord. On screening for the anticancer activity of the metabolites of these isolates, one strain MCCB248 showed promising activity on NCI-H 460 cell lines. 16S rRNA gene sequence of the isolate revealed that the isolate MCCB 248 was a member of the genus *Streptomyces*. The ethyl acetate extract of *Streptomyces* sp. MCCB 248 was tested for the apoptotic induction. Apoptosis mediated anticancer activity of the extract was evaluated and confirmed through cell based assays. Hoechst 33342 staining assay revealed that treated cells showed shrinkage of cell nucleus, fragmentation and chromatin condensation. TUNEL assay also demonstrated that *Streptomyces* sp. MCCB 248 triggered DNA damage as evident from the condensed TUNEL positive chromatin with in cell nuclei. Confirmation of apoptosis by staining with Annexin V-FITC/propidium iodide (PI) showed that the cells treated with extract rapidly undergo apoptosis as obvious from the more percentage of Annexin positive cells in the early hours of treatment. Our result showed that *Streptomyces* sp. MCCB 248 isolated from Arctic environment is a promising candidate for a potent anticancer agent that is under current investigation.

### **Marine Toxins and Bioassays** 

#### **Honaucin A, Mechanism of Action and Potential Role as a Cancer Prevention Agent** 

**Lena** **Gerwick, Samantha Mascuch, Gabrial Navarro, Paul Boudreau, Tristan Carland, Terry Gaasterland and William Gerwick**

University of California San Diego, La Jolla, CA, USA

Three related natural products, the honaucins A–C, were isolated from a cyanobacterium overgrowing a coral reef in Hawaii. Subsequent biological investigations revealed that these molecules inhibit both prokaryotic quorum sensing and eukaryotic inflammation. The honaucins were originally identified as molecules of interest in an *in vitro* assay that quantified its ability to attenuate nitric oxide production in LPS-stimulated macrophages. Continued experiments using honaucin A displayed a transcriptional down-regulation of IL-6, TNFα, IL-1β, and iNOS in these cells. Additionally, *in vivo* anti-inflammatory activity in a murine model of ear edema was demonstrated. To uncover the mechanism of action of honaucin, RNA deep sequencing was performed using total RNA from honaucin A-treated macrophages. Analysis of differentially regulated transcripts strongly suggested that honaucin A is an activator of a pathway which results in the transcription of cytoprotective genes. This signaling pathway has recently drawn interest for its potential application to the treatment of neurodegenerative and autoimmune diseases, as well as cancer. Experiments involving reporter assays and protein pull down using a biotinylated probe to validate the proposed target will be discussed.

#### **Palytoxins from Marine Coastal Environments and Home Aquaria. What Role Do They Play in Human Inhalatory Poisonings?** 

**Carmela Dell’Aversano ^1^, Luciana Tartaglione ^1^, Martino Forino ^1^, Patrizia Ciminiello ^1^, Andre Wieringa ^2^ and Aurelia Tubaro ^3^**

^1^ Department of Pharmacy, University of Napoli Federico II, Napoli, Italy

^2^ Department of Clinical Pharmacy, Isala, Zwolle, The Netherlands

^3^ Department of Life Science, University of Trieste, Trieste, Italy

Since the late 1990s’, hundreds of cases of respiratory illness and/or dermatitis have been repeatedly recorded in people concomitantly with massive proliferations of the benthic dinoflagellate *Ostreopsis* cf. *ovata* in the Mediterranean area. Thanks to development of a liquid chromatography-high resolution mass spectrometry (LC-HRMS) method, we have characterized *O. cf.* *ovata* as a producer of congeners of palytoxin, a highly potent toxin whose inhalation hazard is however unknown. On the basis of the concomitance of *Ostreopsis* blooms, respiratory illness in humans, and detection of palytoxin congeners in algal samples, a cause and effect relationship between the cases of malaise and the algal toxins has been postulated but never substantiated. Further cases of respiratory illness tentatively attributed to palytoxins have been reported for aquarium hobbyists from incidental inhalation of steams generated during cleaning operations of home aquaria containing soft corals belonging to *Palythoa* genus. The only common feature between *Palythoa* spp. and *Ostreopsis* spp. is that both, although phylogenetically distinct, may produce palytoxins. The study reported herein serves the double purpose of demonstrating the presence of palytoxins in home marine aquaria involved in several inhalatory poisonings and of correlating the symptoms shown by patients while handling* Palythoa* spp. in home aquaria with those reported for *Ostreopsis*-related poisonings. From the chemical and symptomatological data it is reasonable to hold palytoxins responsible for respiratory disorders following inhalation. Although the exact mechanism through which palytoxin congeners from *Palythoa* spp. and *Ostreopsis* spp. exert toxicity by inhalation is still unknown, this represents a step toward inhalatory risk assessment of palytoxin congeners in domestic and open-air environments.

#### **Discovery of Fish Killing Toxins from the Microalgae *Prymnesium parvum*** 

**Thomas Ostenfeld Larsen ^1^, Silas Anselm Rasmussen ^1^, Kristian Fog Nielsen ^1^, Sebastian Meier ^2^, Jens Ølgaard Duus ^2^, Hannah Blossom ^3^, Nikolaj Gedsted Andersen ^3^ and Per Juel Hansen ^3^**

^1^ Department of Systems Biology, Technical University of Denmark, Kgs. Lyngby, Denmark

^2^ Department of Chemistry, Technical University of Denmark, Kgs. Lyngby, Denmark

^3^ Marine Biology Section, University of Copenhagen, Helsingør, Denmark

Marine fish farming has the potential to become an economically important industry worldwide. However, blooms of ichthyotoxic (fishkilling) harmful microalgae are a recurring phenomenon in coastal marine waters with some times huge impacts on wild fish stocks as well as caged fish. This has detrimental consequences for the implicated fish farmers, in addition to recreational and commercial fishing. This paper will describe results obtained in the collaborative Danish Strategic Research project: “HABFISH—Harmful algae and fish kills”. Our initial efforts have been directed towards analysis of the chemistry and bioactivities related to *Prymnesium parvum*, an important microalgae in coastal waters. We found huge differences in ichtyotoxicity among 5 different *P. parvum* strains. Chemical analysis based on LC-DAD-MS analysis showed that all strains produced GAT toxins and oleamides [1]. However, we found no evidence for ichthyotoxicity with ecological relevant concentrations, thus excluding that these compounds can be the true ichtyotoxins as recently reported. Furthermore, ^13^C feeding studies showed that oleamides are not true *P. parvum* metabolites, but instead contaminants derived from plastics during sample purification [1]. Excitingly, we found that different strains of *P. parvum* produce at least to types of prymnesin-like molecules. One strain produce prymnesin 1 & 2, whereas several Scandinavian strains produce a novel type of prymnesins. Cultivation of >100 liters of algal medium has allowed us to isolate enough material of a novel prymnesin-like molecule, including a ^13^C enriched version. This paper will report our efforts towards structural elucidation of this large and complex polyketide derived polyether, based on various 2- and 3-D NMR experiments.

##### ***Reference*** 

1. Blossom, H.E.; Rasmussen, S.A.; Andersen, N.G.; Larsen, T.O.; Nielsen, K.F.; Hansen, P.J. *Prymnesium parvum* revisited: Relationship between allelopathy, ichtyotoxicity, and chemical profiles in 5 strains. *Aquat. Toxicol.* **2014**, *157*, 159–166.

#### **On the Mechanisms of Cancer Cell Death Induced by Marine Sponge Depsipeptides: A Comparison of Different *in Vitro* Methodologies** 

**Marisa Rangel ^1,2^, Graziella A. Joanitti ^3^, Ricardo B. Azevedo ^3^, Wagner Fontes ^4^ and Mariana S. Castro ^2,4^**

^1^ Lab. of Immunopathology, Butantan Institute, São Paulo/SP, Brazil

^2^ Lab. of Toxinology, Dept. of Physiological Sciences/IB, University of Brasilia Brasilia/DF, Brazil

^3^ Lab. of Nanobiotechnology, University of Brasilia, Brasilia/DF, Brazil

^4^ Lab. of Biochemistry and Protein Chemistry, Dept. of Cell Biology/IB, University of Brasilia, Brasilia/DF, Brazil

Geodiamolides are depsipeptides from marine sponges that disrupt microfilaments of sea urchin eggs and human breast cancer cell lines. Geodiamolide-H was further tested in breast cancer cells cultures in three-dimentional environment, showing stronger effect on the aggressive Hs578T cells, inhibiting migration and invasion, and reversing its malignant phenotype to polarized spheroid-like structures. The cell death pathways induced by the geodiamolides in the cancer cells, however, have not yet been elucidated. The goal of this work is to study the effects of the geodiamolides-A and H in two different cancer cell lines (4T1, mouse breast tumor; and A431, human nonmelanoma epithelial carcinoma) using different methods, in order to elucidate the mechanisms of cell death. First, using MTT, the geodiamolide-H did not reduce cell viability of both lineages, even at 1 µM. Geodiamolide-A, however, reduced the viability of A431 cells, with IC_50_ of 368 nM. On a second assay, we used a kit to measure three different parameters. The cell viability and cytotoxicity was measured by the activity of two proteases, while apoptosis was measured by the caspase-3/7 activities (Promega ApoTox-Glo^™^ Triplex Assay). In this assay, the geodiamolides did not reduce the viability in the 4T1 cells, or increased membrane permeability, but activated the caspase-3/7. In A431 cells, only the geodiamolide-A induced a decrease the viability, confirming the MTT results. Additionally, geodiamolide-A activated the caspase-3/7. These results indicate that the geodiamolides-A and H may have different effects in these cell lineages, but activated the apoptotic pathway in both. The human cancer cells lineage was more susceptible to the treatment with geodiamolide-A than the murine cells, what was also observed in previous studies, reinforcing the pharmaceutical potential of these molecules. Further experiments will be performed in order to unravel the mechanism of action triggered by these promising antitumoral marine sponge depsipeptides.

**Funding:** CNPq

#### ***In Vitro* Efficacy of Nannochloropsis Gaditana Extract on Primary Human Dermal Fibroblasts as Cosmeceutical Bioactive Ingredient** 

**Sophia Letsiou ^1^, Konstantinos Gardikis ^1^, Lalia Lalia Mantecón ^2^, Carlos Unamunzaga ^2^ and Emmanouil Flemetakis ^3^**

^1^ APIVITA S.A., Scientific Affairs, Industrial Park of Markopoulo Mesogaias, 19003 Markopoulo Attiki, Athens, Greece

^2^ Fitoplanton Marino, S.L. Dársena Comercial s/n (Muelle Pesquero), 11500 El Puerto de Santa María (Cádiz), Spain

^3^ Laboratory of Molecular Biology, Department of Biotechnology, School of Food, Biotechnology and Development, Agricultural University of Athens, Athens, Greece

Nowadays, there is a huge interest on natural products obtained from marine organisms that can promote the state of health and well-being for humans. Microalgae represent a primary source of bioactive compounds and could be used as functional ingredients in cosmetic formulations. The aim of the present study is to evaluate *in vitro* effects of Nannochloropsis gaditana (NannoG) extract in cytoprotection against oxidative stress using H_2_O_2_ as stressor in primary normal human dermal fibroblasts (NHDF), so as to investigate the potential applications of NannoG in cosmetics. In order to gain an insight into the molecular mechanisms of NannoG bioactivity, we employed a newly developed RT-qPCR platform for studying transcript accumulation for an array of genes (more than 100) involved in many skin-related processes, including anti-aging, hydration, oxidative stress response *etc.* NHDF cells were purchased from Lonza Clonetics™. For the oxidative stress evaluation, H_2_O_2_ was used as stressor. Cells were incubated for 48 h with NannoG extract. After incubation and prior to H_2_O_2_ induction the cells were washed two times with PBS and then H_2_O_2_ was added to the cells for 3 h at varying concentrations. Cytotoxicity was assessed by determining the ATP levels. Fibroblasts incubated with NannoG extract and stressed with H_2_O_2_ showed a significant increase in cell viability. Antioxidant, antiaging protection of NannoG extract on NHDF was confirmed by the regulation of several related transcripts (including GPX1, SIRT3) involved in the relative pathways. In addition, an increase in the expression of genes related to hydration process (acquaporins) was observed, under oxidative stress, upon treatment with NannoG. These findings indicate that *N. gaditana* extracts possess strong antioxidant properties and provide new insights into the beneficial role of microalgae bioactive compounds in cosmetic formulations protecting skin from oxidative stress and aging.

### **Dereplication Metabolomics, and Rational Approaches to Bioprospecting** 

#### **Metabolomic Tools to Target and Accelerate the Isolation of Bioactive Compounds from Marine Microbial Symbionts** 

**L. MacIntyre ^1^, M.L. Fazenda ^1^, C. Viegelmann ^1^, T. Zhang ^1^, C. Dowdells ^1^, L. Young ^1^, C. Clements ^1^, G. Abbott 1, K.R. Duncan ^2^, D. Green ^2^, L.M. Harvey ^1^, B. McNeil 1 and R. Edrada-Ebel ^1^**

^1^ University of Strathclyde, Glasgow, Scotland, UK

^2^ Scottish Association for Marine Science, Oban, Scotland, UK

Marine microbial symbionts produce a plethora of novel secondary metabolites which may be structurally unique with interesting pharmacological properties. Some of these compounds can be produced on a biotechnological scale using fermentation processes and are therefore an economically viable and sustainable source of commercial quantities of metabolites of interest. Mass spectrometry (MS) and nuclear magnetic resonance (NMR) spectroscopy-based metabolomics are readily applicable to microbial drug discovery efforts, offering the ability to comprehensively analyse the chemical composition of complex mixtures, e.g., broth extracts, under a given set of conditions in a highly efficient manner. Metabolomic methods were integrated with chemoinformatic approaches and bioassay screening results to monitor metabolite production by several strains of marine bacteria under various culture conditions. Metabolomic tools were then used to complement bioassay-guided fractionation by rapidly targeting novel bioactive secondary metabolites, accelerating the isolation and purification of new natural products. The application of this methodology within the SeaBioTech* project shall be presented.

##### ***Acknowledgments*** 

This work was supported by the SeaBioTech project that is funded by the European Commission within its FP7 Programme, under the thematic area KBBE.2012.3.2-01 with Grant Number 311932.

#### **An Integrated Approach for Bioprospecting Novel Bioactive Compounds from Marine Actinomycetes** 

**Paula Jimenez ^1,2^, Larissa Guimarães ^2^, Danilo Rocha ^2^, Elthon Ferreira ^2^, Maria da Conceição Torres ^2^, Otília Pessoa ^2^, Eric Lau ^3^, Damian Mason ^3^, Eli Chapman ^3^, James La Clair ^4^ and Leticia Costa-Lotufo ^5^**

^1^ Universidade Federal de São Paulo, Santos, SP, Brazil

^2^ Universidade Federal do Ceará, Fortaleza, CE, Brazil

^3^ University of Arizona, Tucson, AZ, USA

^4^ Xenobe Research Institute, San Diego, CA, USA

^5^ Universidade de São Paulo, São Paulo, SP, Brazil

Conventional search strategies of new drugs from natural products comprise the bioactivity-guided chemical fractionation of an extract leading to isolation of the active principle. An innovative approach involves that guided by a specific cellular protein or pathway as a biological target. Herein, we describe the bioprospection of compounds from an extract obtained from a strain of *Actinomadura* sp. recovered from marine sediments applying both, bioactivity and target-directed strategies. For bioactivity-guided fractionation, MTT assay was used to evaluate the cytotoxicity of the crude extract, derived fractions and pure molecules against the HCT-116 tumor cell line. For the target-directed approach, a functional chromatography protocol, a process of reverse affinity chromatography, was applied to collect compounds that bound to survivin, an inhibitor of apoptosis protein. Briefly, recombinantly expressed human survivin was covalently linked to a resin, which, in turn, was incubated with the extract. Unbound compounds were washed off and survivin was denatured with ethanol to recover the retained material. The ethanolic extract was then analyzed by LC-HRMS and µ-scale NMR to identify signals of molecules contained therein. Theses signals were compared to molecular masses in the AntiMarin database to search for matches. Bioactivity-guided fractionation yielded nonilprodigiosin and cyclononilprodigiosin, two members of the prodigiosins family, which are known for their cytotoxic activity. These molecules were major components of this extract. For the target-directed methodology, LC-MS analysis of the whole, pre-chromatography extract returned masses for 36 compounds, while the material eluted from resin contained 2 masses, 522.3038 and 536.3257, which were unmatched in the database, suggesting they could be new compounds. The next steps involve isolation of compounds and validation of their modulatory effect using biological models. However, these preliminary results highlight the efficiency of this approach in fishing out molecules from a complex crude extract.

#### **Comparative Metabolomics of Secondary Metabolites from Bacteria Using GNPS Molecular Networking** 

**Katherine Duncan ^1^, Lynsey MacIntyre ^2^, David Green ^1^, RuAngelie Edrada-Ebel ^2^ and Michele Stanley ^1^**

^1^ Scottish Association for Marine Science, Oban, UK

^2^ Strathclyde Institute of Pharmacy and Biomedical Sciences, University of Strathclyde, Glasgow, UK

Genome sequencing has revealed many more microbial biosynthetic pathways then would be predicted based on the numbers of secondary metabolites they produce. These “silent” gene clusters may in fact be expressed but the products missed using traditional natural product discovery tools. The generation of molecular networks based on MS-MS data provides a rapid and highly sensitive approach to visualize secondary metabolite profiles in extracts of cultured bacteria. This technique facilitates strain comparison and can be used to prioritize strains for more detailed discovery efforts. Here we have generated molecular networks for several biotechnologically valuable strains (including *Micromonospora*, *Streptomyces*,* Vibrio splendidus* and *Rhodococcus *sp.) isolated from understudied marine environments (Antarctic, Scotland, Mediterranean sponges) some of which have draft genome sequences available. The networks were populated with standards that aid in chemical identification. This has allowed identification of previously isolated secondary metabolites (avoiding redundant chemical purification) and the discovery of potentially novel secondary metabolites. Select nodes have the potential to be linked to genome sequencing data in an effort to identify the associated biosynthetic pathways and facilitate structure predictions. Combining new fermentation approaches with molecular networking provides opportunities to improve the efficiency with which natural products are discovered.

#### **Bioprospecting Portuguese Atlantic Coast Cyanobacteria for Bioactive Secondary Metabolites Reveals Untapped Chemodiversity** 

**Ângela Brito ^1,2^, Joana Gaifem ^1^, Vitor Ramos ^3^, Evgenia Glukhov ^4^, Pieter C. Dorrestein ^5,6^, William H. Gerwick ^4,6^, Vitor M. Vasconcelos ^2,3^, Marta V. Mendes ^1^ and Paula Tamagnini ^1,2^**

^1^ i3S—Instituto de Investigação e Inovação em Saúde & IBMC—Instituto de Biologia Molecular e Celular, Universidade do Porto, Porto, Portugal

^2^ Faculdade de Ciências, Departamento de Biologia, Universidade do Porto, Porto, Portugal

^3^ CIIMAR/CIMAR—Interdisciplinary Centre of Marine and Environmental Research, University of Porto, Porto, Portugal

^4^ Center for Marine Biotechnology and Biomedicine, Scripps Institution of Oceanography, University of California San Diego, La Jolla, USA

^5^ Department of Chemistry and Biochemistry, University of California San Diego, La Jolla, USA

^6^ Skaggs School of Pharmacy and Pharmaceutical Sciences, University of California San Diego, La Jolla, USA

Cyanobacteria produce a large array of bioactive compounds, some of which are toxic to different organisms, including animals and humans, while others possess promising therapeutic applications such as anticancer, antibiotic and anti-inflammatory activities. Marine cyanobacteria, in particular, are increasingly being recognized as important source of structurally diverse secondary metabolites. In a previous study, several cyanobacterial strains were isolated from the Portuguese coast and characterized using a polyphasic approach [1]. To evaluate the potential of our isolates to produce bioactive compounds, we performed a PCR screening for the presence of genes encoding non-ribosomal peptide synthetases (NRPSs) and polyketide synthases (PKSs), targeting the adenylation (A) and ketosynthase (KS) domains, respectively. DNA fragments were obtained for more than 80% of the strains tested and the sequences obtained were used for an *in silico* prediction of the PKS and NRPS systems. Moreover, in several selected strains RT-PCR analyses revealed that these genes are transcribed under routine laboratory conditions. Furthermore, LC-MS analysis coupled with molecular networking, a mass spectrometric tool that clusters metabolites with similar MS/MS fragmentation patterns [2], was used to search for novel or otherwise interesting metabolites in crude extracts of our isolates. Our results revealed an untapped chemodiversity in marine cyanobacterial isolates from the temperate region of Portugal and validate the LC-MS analyses coupled with molecular networking as a powerful tool for their discovery [3].

##### ***References*** 

1. Brito, A.; Ramos, V.; Seabra, R.; Santos, A.; Santos, C.L.; Lopo, M.; Ferreira, S.; Martins, A.; Mota, R.; Frazão, B*.*; *et al.* Culture-dependent characterization of cyanobacterial diversity in the intertidal zones of the Portuguese coast: A polyphasic study. *Syst. Appl. Microbiol.*
**2012**, *35*, 110–119.
2. Winnikoff, J.R.; Glukhov, E.; Watrous, J.; Dorrestein, P.C.; Gerwick, W.H. Quantitative molecular networking to profile marine cyanobacterial metabolomes. *J. Antibiot.*
**2014**, *67*, 105–112.
3. Britoa, A.; Gaifema, J.; Ramosd, V.; Glukhove, E.; Dorresteinf, P.C.; Gerwicke, W.H.; Vasconcelosc, V.M.; Mendesa, M.V. Paula Tamagnini Bioprospecting Portuguese Atlantic coast cyanobacteria for bioactive secondary metabolites reveals untapped chemodiversity. *Algal Res.*
**2015**, *9*, 218–226.

#### **Natural Product Discovery Guided by Genome Sequence of Mangrove-12 ptDerived *Streptomyces qinglanensis* 172205** 

**Kui Hong, Dongbo Xu, Yi Yu and Zixin Deng**

Key Laboratory of Combinatorial Biosynthesis and Drug Discovery, School of Pharmaceutical Sciences, Wuhan University, Wuhan, Hubei, China

With the rapid development of genome sequencing and the availability of genome information in NCBI database, natural product discovery from microorganisms is undergoing a transformation from traditional “forward” approach based on bioactivity, color or chemical property tracking to genome-driven “reverse” strategy including compounds structure and properties prediction, OSMAC (one strain many compounds), promoter strengthening, regulatory gene manipulation, heterologous expression, *etc.* In this report, we present natural products discovery from a marine actinomycete using the “reverse” strategy together with the “forward” approach, which resulted at least 5 kinds of compounds. *Streptomyces qinglanensis *172,205 is a novel species isolated from a mangrove soil sample in Hainan, China. Its genome size is about 7.2 M. Twenty one gene clusters for secondary metabolites biosynthesis were predicted by antiSMASH and functional genes were annotated. Employing the “reverse” strategy, guided by the gene cluster prediction, three compounds of enterocin, ectoine and lysolipin were detected by LC-MS after OSMAC. Enterocin which was the dominant compound of strain 172,205 was confirmed by compound isolation and NMR characterization. Deletion of the gene cluster responsible for biosynthesis of enterocin improved the detection and isolation of the other low content compounds. With the “forward” approach based on bioactivity screening and high-resolution MS database searching, two other compounds PreQ_0_ base and coproporphyrin III were identified by LC-MS or NMR. Furthermore, the isolated compounds enterocin and PreQ_0_ were subjected to bioactivity screening such as antimicrobial, antitumor, α-glucosidase and β-amyloid protein (Aβ_1–42_) fibrillation inhibitory activities, and new bioactivities of these compounds were uncovered.

### **Advances in Isolation and Structure Elucidation** 

#### **Isolation and Quantification of Biosynthetic Cyclic Peptides** 

**Rosemary Adaba ^1^, Wael Houssen ^1^, Andrew McEwan ^1^, Jioji Tabudravu ^1^, Andrea Raab ^1^, Louis Thomas ^1^, Nathalie Pieiller ^1^, Andrew Bent ^2^, Marcel Jaspars ^1^ and Jim Naismith ^2^**

^1^ University of Aberdeen, Aberdeen, UK,

^2^ University of St Andrews, St Andrews, UK

Cyclic peptides hold a great promise as potential therapeutics for a range of diseases [1]. One class of peptides, the cyanobactins, originating from marine cyanobacteria are characterized by the incorporation of heterocycles, d-stereocentres and possibly *O*-prenylated residues [2–8]. One potential therapeutic application is the inhibition of the drug efflux pump in cancer cells [9]. Generating analogues of these compounds may improve their therapeutic utility. This range of compounds cannot presently be made by chemical synthesis, but biosynthetically through genetic engineering, milligram quantities of these modified peptides have being successfully produced [10]. Optimal purification and accurate quantification of these novel compounds is a pre-requisite for biological evaluation. The use of analytical methods such as UV detection, mass spectrometry or radioisotope labeling cannot be use to accurately quantify such compound considering the individual limitations of these methods. These peptides contain sulphur in form of cysteines, methionines and other forms of sulphur containing amino acids which can be used for species unspecific quantification by LC-ICP-MS. We have subjected these cyclic peptides of varied amino acid sequences to various purification steps followed by LC-ICP-MS quantification, using ESMS for identification purposes only, since no pure compounds are available for use as standards. The purpose was to confirm that the choice of purification techniques for the synthesized mixtures are adequate and accurate quantification of these pure compounds using the amount of sulphur in each peptide sequence will become a critical part of improving compound recovery.

##### ***References*** 

1. Giordanetto, F.; Kihlberg J. Macrocyclic drugs and clinical candidates: What can medicinal chemists learn from their properties? *J. Med. Chem.*
**2014**, *57*, 278–295.
2. Arnison, P.G.; Bibb, M.J.; Bierbaum, G.; Bower, A.A.; Bugni, T.S.; Bulaj G. Camarero, J.A.; Campopiano, D.J.; Challis, G.L.; Clardy, J.; *et al.* Ribosomally synthesized and post-translationally modified peptide natural products: Overview and recommendations for a universal nomenclature. *Nat. Prod. Rep.*
**2013**, *30*, 108–160.
3. Houssen, W.E.; Jaspars, M. Azole-based cyclic peptides from the sea squirt *Lissoclinum patella*: Old scaffolds, new avenues. *ChemBioChem*
**2010**, *11*, 1803–1815.
4. Schmidt, E.W.; Nelson, J.T.; Rasko, D.A.; Sudek, S.; Eisen, J.A.; Haygood, M.G.; Ravel J. Patellamide A and C biosynthesis by a microcin-like pathway in *Prochloron didemni*, the cyanobacterial symbiont of *Lissoclinum patella. Proc. Natl. Acad. Sci. USA*
**2005**, *102*, 7315–7320.
5. Long, P.F.; Dunlap, W.C.; Battershill, C.N.; Jaspars, M. Shotgun cloning and heterologous expression of the patellamide gene cluster as a strategy to achieving sustained metabolite production. *ChemBioChem*
**2005**, *6*, 1760–1765.
6. Donia, M.S.; Ravel, J.; Schmidt, E.W. A global assembly line to cyanobactins. *Nat. Chem. Biol.*
**2008**, *4*, 341–343.
7. Koehnke, J.; Bent, A.F.; Zollman, D.; Smith, K.; Houssen, W.E.; Zhu, X.; Mann, G.; Lebl, T.; Scharff, R.; Shirran, S.; *et al.* The cyanobactin heterocyclase enzyme: A processive adenylase that operates with a defined order of reaction. *Angew. Chem. Int. Ed. *
**2013**, *52*, 13991–13996
8. McIntosh, J.A.; Donia, M.S.; Schmidt, E.W. Insights into heterocyclization from two highly similar enzymes. *J. Am. Chem. Soc.*
**2010**, *132*, 4089–4091.
9. Aller, S.G.; Yu, J.; Ward, A.; Weng, Y.; Chittabonia, S.; Zhuo, R.; Harrell, P.M.; Trinh, Y.T.; Zhang, Q.; Urbatsch, I.L.; Chang, G. Structure of P-glycoprotein reveals a molecular basis for poly-specific drug binding. *Science*
**2009**, *323*, 1718–1722.
10. Houssen, W.E.; Bent, A.F.; McEwan, A.R.; Pieiller, N.; Tabudravu, J.; Koehnke, J.; Mann, G.; Adaba, R.I.; Thomas, L.; Hawas, U.W.; *et al*. An efficient method for the *in vitro* production of azol(in)e-based cyclic peptides. *Angew Chem. Int. Ed.*
**2014**, *53*, 14171–14174.

#### **Oxygenated Polyketides from a Chinese Plakortis Sponge. A Treasure Trove of Structurally Diversity and Biological Activity** 

**Giuseppina Chianese ^1^, Fan Yang ^2^, Hue-Wen Lin ^2^, Shabana Khan ^3^ and Orazio Taglialatela-Scafati ^1^**

^1^ Università degli Studi di Napoli Federico II, Naples, Italy

^2^ Shanghai Jiao Tong University, Shanghai, China

^3^ University of Mississippi, University, MS, USA

Chemical analysis of the organic extract obtained from the sponge Plakortis simplex collected in the South China Sea afforded a series of oxygenated polyketides, belonging to different structural classes. Several of these compounds were new and some of them proved to be characterized by an unprecedented carbon skeleton. Their detailed stereostructural characterization was based on extensive spectroscopic and computational analyses, including GIAO 13C-NMR and ECD calculations. Among the isolated compounds, endoperoxide derivatives exhibited *in vitro* antimalarial activity against chloroquine-resistant Plasmodium falciparum strains, while non-endoperoxide polyketides were tested on PPAR-α and PPAR-γ, revealing an interesting profile of activity. The isolation of this complex pattern of related compounds inspired an investigation aimed at clarifying the origin of the many polyketides found in sponges of this genus. Thus, the major polyketide endoperoxide isolated from this organism, haterumadioxin A (**1**), was allowed to react in reducing, acidic and basic media, and the extensive rearrangements experienced by the natural product yielded to the formation of several compounds, some of which previously described as natural products. In this lecture, details on structure elucidation, biological activities and possible biogenetic origin of the isolated compounds will be discussed.

#### **Residual Dipolar Couplings in the NMR-Based Configurational Analysis of NEW Cystochromanes from the Phaeophyta *Cystoseira baccata*** 

**Julie Muñoz ^1^, Alexis Krupp ^2^, Stefan Immel ^2^, Michael Reggelin ^2^, Gerald Culioli ^3^ and Matthias Köck ^1^**

^1^ Alfred-Wegener-Institut, Helmholtz-Zentrum für Polar- und Meeresforschung, Am Handelshafen 12, 27570 Bremerhaven, Germany

^2^ Clemens-Schöpf-Institut für Organische Chemie und Biochemie, Technische Universität Darmstadt, Alarich-Weiss-Straße 4, 64287 Darmstadt, Germany

^3^ Université de Toulon, MAPIEM, EA 4323, 83957 La Garde cedex, France

The systematic investigation of the phaeophyta *Cystoseira* *baccata* actually revealed seven new meroditerpenes, the cystochromanes A–G. A detailed study of the relative and absolute configurations of the new molecules as well as a model compound (the structurally simplest member of the family) was carried out. For the determination of the relative configuration, NOE-derived interproton distances were used as input for floating chirality DG/DDD simulations. Since the number of NOEs was not sufficient for an unambiguous assignment of the relative configuration, residual dipolar couplings (RDCs, measured in lyotropic liquid crystalline phases of chiral polyarylacetylenes) were used to refine the structures. The absolute configurations of the new compounds were assessed by comparison of their circular dichroism (CD) spectra to the calculated ones. *Cystoseira* is one of the most studied genus of the Sargassaceae family and *Cystoseira* spp. are known to produce a wide array of terpenes, such as linear diterpenes or meroditerpenes. Even though these compounds have been studied for more than 40 years, it was recently demonstrated that their structures could still raise interesting issues, especially concerning their stereochemistry. The isolation and structure elucidation of the meroditerpenes from *Cystoseira* *baccata* led to a strong indication for a revision of the bicyclo[4.3.0]nonane system characteristic for compounds of this family.

#### **Chlorinated Peptide/Polyketide Hybrids from the Caribbean Sponge *Smenospongia aurea*: A Comprehensive Approach** 

**Roberta Teta ^1^, Gerardo Della Sala ^1^, Germana Esposito ^1^, Alessia Caso ^1^, Roberta Miceli ^2^, Luca S. Ceccarelli ^2^, Rosa Camerlingo ^2^, Elena Irollo ^2^, Giuseppe Pirozzi ^2^, Valeria Costantino ^1^ and Alfonso Mangoni ^1^**

^1^ The NeaNat Group, Dipartimento di Farmacia, Università degli Studi di Napoli Federico II, Napoli, Italy

^2^ Department of Experimental Oncology, Istituto Nazionale Tumori Fondazione “G. Pascale”, Napoli, Italy

The Caribbean sponge *Smenospongia aurea *was recently shown to contain a series of chlorinated mixed-biogenesis NRPS/PKS products with interesting cytotoxic activities, which make them promising leads for antitumor drug design. In addition to the published smenamide A (**1**) and B (**2**) [1] and smenothiazole A (**3**) and B (**4**) [2], *S. aurea* contains several additional compounds belonging to this class. Overall, the structures of these peculiar peptide/polyketide hybrids suggest that they may be products of the cyanobacterial metabolism. The most recent results of a comprehensive approach to the study of this peculiar class of metabolites will be discussed, including structural elucidation, synthesis, and biosynthetic origin.

##### ***References*** 

1. Teta, R.; Irollo, E.; della Sala, G.; Pirozzi, G.; Mangoni, A.; Costantino, V. Smenamides A and B, Chlorinated Peptide/Polyketide Hybrids Containing a Dolapyrrolidinone Unit from the Caribbean Sponge *Smenospongia aurea*. Evaluation of Their Role as Leads in Antitumor Drug Research. *Mar. Drugs*
**2013**, *11*, 4451–4463.
2. Esposito, G.; Teta1, R.; Miceli, R.; Ceccarelli, L.S.; della Sala, G.; Camerlingo, R.; Irollo, E.; Mangoni, A.; Pirozzi, G.; Costantino, V. Isolation and Assessment of the *in Vitro* Anti-Tumor Activity of Smenothiazole A and B, Chlorinated Thiazole-Containing Peptide/Polyketides from the Caribbean Sponge, *Smenospongia aurea*. *Mar. Drugs*
**2015**, *13*, 444–445.

### **Industrial Biotechnology, Polymers and Biomolecules** 

#### **Development of Synthetic Biology Tools to More Predictably Clone, Express and Select Biocatalytic Activities for Metabolic Pathway Optimization and High Yield Biomolecule Production** 

**David McElroy, Ian Fotheringham and Alison Arnold**

Ingenza, Ltd., Roslin, Edinburgh, UK

Ingenza’s has developed a number of proprietary synthetic biology tools to enable more predictably engineer biological systems for the production of commercially relevant examples. These tools include protein engineering to address poor response during the control of gene expression, the development of synthetic landing pads to optimize the genomic operating environment around delivered genes, the use of genome editing and RNA trafficking systems to control gene expression, the application of transciptomics and metabolomics to enhance cell system performance, the development of synthetic gene expression regulatory elements to better control gene expression and the deployment of our proprietary inABLE combinatorial genetics platform for large scale gene/pathway assembly and optimization. Together these tools have been used to rapidly clone, express, select and optimize target activities for many separate enzymatic reactions, from thousands of independent genes derived from metagenomic and phylogenetic discovery approaches. Obvious synergy exists between this approach and versatile, solid phase screening and selection methods using growth-based, cross-feeding or colourimetric methods to identify engineered cells of interest. This is illustrated through the rapid identification of critical pathway enzymes, optimal gene coding sequences and enzyme variants from inABLE^®^-derived high quality variant libraries for industrial applications in bio-based polymers, chemicals and personal care products with our commercial customers. We will describe the success of modeling approaches to gene design that enhance the predictability of heterologous gene expression in diverse hosts. In developing this suite of technologies we aims to bring increasing predictability and overcome persistent limitations associated with today’s iterative and empirical processes for microbial strain improvement resulting in faster routes to high yielding biomolecule production. We will exemplify this approach with reference to commercially relevant examples in the field of biopolymer and therapeutic protein production.

#### **Protein Superfamily Data Integration for Protein Function Elucidation and Optimisation** 

**Tom van den Bergh ^1,2^ and Henk-Jan Joosten ^1^**

^1^ Bio-Prodict, Nijmegen, The Netherlands

^2^ Wageningen University, Wageningen, The Netherlands

Nature offers a wide variety of enzymes that can be utilized in many different processes. Metagenomics studies allow us to find many new enzymes from marine sources, however the exact function of new enzymes if not always clear. Here we present 3DM, a protein superfamily analysis platform that integrates many different data types (such as sequences, structures, function data, literature, reaction data *etc.*) for complete protein superfamilies. This platform can be used to assign function to proteins and their amino acids. New enzymes often need to be optimized by introducing mutations to meet the requirements of a (biotechnological) process. In many cases multiple mutations are needed to reach these goals, but finding the right combination of mutations still is problematic. More and more protein-engineers use a strategy referred to as “smart library design”. Smart mutant libraries contain only a small number of mutants and are designed such that they contain a high number of active clones with mutations at positions (called hotspots) that are likely to show the desired effect. Using 3DM different enzymes features, such as enantioselectivity, activity and thermostability have been optimized. Comparisons with random designed libraries show that using 3DM when designing a smart library results in high quality libraries reducing not only the number of clones that need to be screened but it also increases the chance of finding an enzyme with the desired properties.

#### **Bioprocessing of Marine Microbes for Industrial Exploitation** 

**Christina Viegelmann, Mariana Fazenda, Lynsey MacIntyre, RuAngelie Edrada-Ebel, Brian McNeil and Linda Harvey**

Strathclyde Institute of Pharmacy and Biomedical Sciences, University of Strathclyde, Glasgow, UK

Marine microorganisms are a relatively untapped resource in the search for biopolymers and bioactive metabolites. This was initially due to limitations in sampling capabilities but now is primarily due to the difficulties in the cultivation of organisms that have evolved to live in unique environments. SeaBioTech, an EU-FP7 project, has isolated microorganisms from extreme marine environments, such as geothermal intertidal biotopes in Iceland, hydrothermal vents in the Eastern Mediterranean Sea and areas of the Scottish coasts. These strains can yield distinctive chemistries which produce metabolites of interest to medicinal and industrial sectors as they have the potential to be used in a wide range of applications. The challenge lies in providing the optimum conditions for the production of these metabolites, as marine microbes do not naturally experience the typical conditions found within bioreactors used in industrial-scale fermentations. Physico-chemical changes in the culture conditions during scale-up can result in subsequent metabolomic variations. The target is therefore to improve our knowledge of process physiology, thus optimising the potential for the production of the metabolites of interest. Our aim is to develop protocols for scaling up the organisms producing these target compounds using laboratory and pilot scale systems. Thus, we will develop systems which will allow exploitation on an industrial scale in the future. In order to achieve this we will use multi-fermenter systems and cross-scale process modelling, combined with metabolomic and chemometric analysis, to deliver new products from marine sources with built-in enhanced manufacturability.

#### **Bio-Silica Glass Formation by Silicatein—Functional Dissection of a Sponge Enzyme with Multiple Applications** 

**Heinz C. Schröder, Xiaohong Wang and Werner E.G. Müller**

University Medical Center of the Johannes Gutenberg University, Mainz, Germany

Bio-silica the material that forms the inorganic skeleton of the siliceous sponges is characterized by exceptional mechanical and optical properties. We have been the first to demonstrate that silicatein is a genuine enzyme which synthesizes polymeric silica from ortho-silicate at concentrations around its Michaelis constant of 100 μM, most likely via reactive cyclic silicic acid species. Self-cleavage of the immature pro-silicatein at the autocatalytic cleavage site glutamine [Q]/aspartic acid [D] into the *N*-terminal pro-peptide and the mature silicatein triggers the molecule not only to become enzymatically active (structure-forming activity) but also to acquire structure-guiding properties (providing the structural platform for the biosilica product via self-assembly of the mature silicatein to dimers, tetramers, pentamers, and finally long insoluble filaments). In order to dissect the biocatalytic, structure-forming activity of silicatein from its structure-guiding function, two differently mutated genes were constructed from the silicatein-α gene of the demosponge *Suberites domuncula*. (i) A gene encoding for a non-processed silicatein that was mutated, by replacing Q/D by Q/Q, at the cleavage site of the primary translation product; (ii) A gene encoding for a mature enzymatically-active silicatein in which the serine [S] stretch (implicated in the binding to silica) was replaced by a Q-stretch. The expressed recombinant proteins were applied for micro-contact printing. The experiments revealed that enzymatically active, structure-forming silicatein, coated around the printed non-enzymatically acting structure-guiding silicatein has the property to synthesize biosilica that can act as a light waveguide. Our results show that the enzymes/proteins involved in bio-silica formation can be applied for the development of novel bioinspired materials for diverse applications in nano-optics and nano-biotechnology. (Supported by EU FP7 grants “BlueGenics” no. 311848, “Bio-Scaffolds” no. 604036, and WEGM: ERC Advanced Grant “BIOSILICA” no. 268476).

#### **Evaluation of Microbial Production of Exopolysaccharide by *Rhodothermus marinus* Strains: Potential for Industrial Biotechnology** 

**Roya R. R. Sardari, Evelina Kulcinskaja, Emanuel Ron and Eva Nordberg-Karlsson**

Lund University, Lund, Sweden

The formation of extracellular polysaccharides (EPS) by *Rhodothermus marinus *DSM 4252 and *Rhodothermus marinus* 493, two wild type species of thermophilic bacteria has been screened in different culture media. Marine broth containing either 1 or 10 g/L of glucose, sucrose, lactose, and maltose, separately was used. The results showed that these two strains have the ability to produce and release the EPS. Marine broth containing 10 g/L lactose showed the highest production of EPS in both strains. Besides, EPS production was mainly during the stationary phase. The monosaccharide composition of the produced exopolysaccharides was analyzed and quantified. The results suggested a heteropolymer structure for produced EPS of both strains. The most abundant monomers were xylose, arabinose, and mannose in all media in both strains. Also, the presence of glucose, galactose was varied depending on the type of media used for production of EPS. The EPS of R. marinus DSM 4252 included high quantity of xylose, while in *R. marinus* 493 arabinose had the highest amount compared to the other produced monosaccharides. The molecular mass of produced EPS by these two strains was determined by size exclusion chromatography technique using Sephacryl S-200 and Sephacryl S-500 columns. These results lead us for further studies aimed at increasing the interest in the application of produced EPS of Rhodothermus marinus in food, pharmaceutical, and wastewater treatment industries by optimizing the condition for high production of EPS and characterizing its physicochemical properties.

### **Organic Synthesis** 

#### **Synthesis of Siderophores from Fish Pathogenic Bacteria** 

**Carlos Jiménez ^1^, Yuri Segade ^1^, Katherine Valderrama ^1^, Rosa M. Nieto ^1^, Jaime Rodríguez ^1^, Miguel Balado ^2^ and Manuel L. Lemos ^2^**

^1^ Universidade da Coruña, A Coruña, Spain

^2^ Universidad de Santiago de Compostela, Santiago de Compostela, Spain

The fish pathogenic bacteria *Aeromonas salmonicida* subsp. *salmonicida* (*Ass*) and *Photobacterium damselae* subsp. *piscicida* (*Phdp*) are the aetiological agents of fish furunculosis and pasteurellosis, respectively, diseases that cause large economic losses in marine aquaculture worldwide [1,2]. We were able to isolate and identify some of the siderophores involved in their iron uptake systems such as acinetobactin from *Ass* and piscibactin from *Phdp* [3]. In order to perform several studies on the iron uptake mechanisms of these bacteria, the synthesis of those siderophores and their analogues were addressed. Acinetobactin (**1**) Piscibactin (**2**): In this communication we will focus on our results on the synthesis of compounds **1** and **2**. The synthesis of ent-acinetobactin and other simplified analogues were already finished and the evaluation of their siderophore activity allowed us to deduce several structure-activity relationships. On the other hand, the advances on the total synthesis of piscibactin will be also presented.

##### ***Acknowledgments*** 

This work was supported by Grant AGL2012-39274-C02-02 (Ministerio de Economia y Competitividad, Spain).

##### ***References*** 

1. Osorio, C.R.; Juiz-Río, S.; Lemos, M.L. A siderophore biosynthesis gene cluster from the fish pathogen *Photobacterium damselae* subsp. *piscicida* is structurally and functionally related to the Yersinia high-pathogenicity island. *Microbiology*
**2006**, *152*, 3327–3341.
2. Najimi, M.; Lemos, M.L.; Osorio, C.R. Identification of siderophore biosynthesis genes essential for growth of *Aeromonas salmonicida* under iron limitation conditions. *Appl. Environ. Microbiol.*
**2008**, *74*, 2341–2348.
3. Souto, A.; Montaos, M.A.; Rivas, A.J.; Balado, M.; Osorio, C.R.; Rodríguez, J.; Lemos, M.L.; Jimenez, C. Structure and Biosynthetic Assembly of Piscibactin, a Siderophore from *Photobacterium damselae *subsp. *piscicida*, Predicted from Genome Analysis. *Eur. J. Org. Chem.*
**2012**, *2012*, 5693–5700.

#### **Synthesis and Antitumour Activity of PM050489 and PM060184 Analogues** 

**Alberto Rodríguez, Isabel Digon, Cristina Mateo, María Garranzo, Carmen Murcia, Andrés Francesch, Simon Munt and Carmen Cuevas**

Pharmamar, Madrid, Colmenar Viejo, Spain

PM050489 and PM060184 belong to a new family of compounds first isolated from the Madagascan sponge *Lithoplocamia lithistoides* [1]. Both are known to be tubulin-binding agents showing antimitotic properties in human tumour cell lines at subnanomolar concentrations and displaying a distinct inhibition mechanism on microtubules [2,3]. A synthetic route for PM060184 and PM050489 has already been developed [1] and PM060184 is obtained on a multi-gram scale under GMP conditions for use in phase I clinical trials. Herein, we describe the structure-activity relationship studies carried out in our department for this new family. Particularly, using variants of the previously reported synthetic route to PM050489 and PM060184, we have prepared and tested the *in vitro* cytotoxicity of many different analogues of this new family.

##### ***References*** 

1. Martín, M.J.; Coello, L.; Fernández, R.; Reyes, F.; Rodríguez, A.; Murcia, C.; Garranzo, M.; Mateo, C.; Sánchez-Sancho, F.; Bueno, S.; *et al*. Isolation and First Total Synthesis of PM050489 and PM060184, Two New Marine Anticancer Compounds. *J*. *Am*. *Chem*. *Soc*. **2013**, *135*, 10164–10171.
2. Pera, B.; Barasoain, I.; Pantazopoulou, A.; Canales, A.; Matesanz, R.; Rodriguez-Salarichs, J.; García-Fernandez, L.F.; Moneo, V.; Jiménez-Barbero, J.; Galmarini, C.M.; *et al*. New Interfacial Microtubule Inhibitors of Marine Origen, PM050489/PM060184, with Potent Antitumor Activity and a distinct Mechanism. *ACS Chem. Biol*. **2013**, *8*, 2084–2094.
3. PM060184, a new tubulin binding agent with potent antitumor activity including P-glycoprotein over-expressing tumors. *Biochem. Pharmacol.*
**2014**, *88*, 291–302.

#### **3D Natural Product Scaffolds: A Starting Point in Drug Discovery** 

**Fatemeh Mazraati Tajabadi, Rebecca Pouwer, Marc Campitelli and Ronald J Quinn**

Eskitis Institute for Drug Discovery, Griffith University, Brisbane, Australia

The vast majority of known synthetic scaffolds, and by extension screening libraries, are planar (Fsp3 values < 0.45), whereas natural product scaffolds tend to be less planar (Fsp3 values > 0.45) [1]. A number of non-flat scaffolds embedded in NP have been identified through the use of cheminformatics. A promising scaffold (cedrane) was subjected to a series of molecular modeling studies. The overlaying of all natural products that contain the cedrane scaffold demonstrates the ability of the scaffold to direct the pendant groups in 3D space. Based on this result, a six-step synthetic plan was utilized to make the scaffold with three orthogonally reactive chemical handles.

##### ***Reference*** 

1. Tajabadi, F.M.; Campitelli, M.; Quinn, R.J. Scaffold Flatness: Reversing the Trend. *Springer Sci. Rev*. **2013**, *1*, 141–151.

#### **Combining Dereplication, Metabolomics and Semi-Synthesis for the Discovery of Drug Lead Cryptic Analogs in Marine-Sourced Fungi** 

**Olivier Grovel ^1^, Elodie Blanchet ^2,3^, Marieke Vansteelandt ^4,5^, Yann Guitton ^6,7^, Catherine Roullier ^1^, Ronan Le Bot ^8^ and Yves François Pouchus ^1^**

^1^ Université de Nantes, Faculté de Pharmacie, MMS, F-44035 Nantes, France

^2^ LBBM, Station marine de Banyuls sur mer, USR3579, UPMC-CNRS, F-66650 Banyuls sur mer, France

^3^ MCAM, Museum National d’Histoire Naturelle, UMR7245, MNHN-CNRS, F-75006 Paris, France

^4^ Université de Toulouse, UPS, UMR 152 Pharma-DEV, Université Toulouse 3, Faculté des Sciences Pharmaceutiques, F- 31062 Toulouse Cedex 09, France

^5^ IRD, UMR 152 Pharma-DEV, F-31062 Toulouse Cedex 09, France

^6^ Université de Rennes 1, CNRS, IRISA UMR 6074, F-35000 Rennes, France

^7^ Université de Nantes, LINA UMR 6241, F-44000 Nantes, France

^8^ ATLANTHERA, F-44000 Nantes, France

The rich biodiversity of marine-sourced microorganisms and the complexity of their enzymatic equipment make them a promising source of structurally diverse and biologically active compounds. Among them, fungi of the genus *Penicillium* produce a wide range of bioactive metabolites and it can be supposed that many more unknown molecules are still to be discovered as new druggable compounds or as new models for medicinal chemistry. Indeed, fungal biosynthetical routes usually lead to many more derivatives than the major compounds which have been isolated and described. However, targeting chemistry on these “side-products” is a challenging task as they are often transient or trace compounds, and their isolation is often bound to fail. To avoid this pitfall, metabolomics can allow a rapid, accurate and highly informative investigation of this cryptic chemical diversity by the hyphenated use of UHPLC, mass spectrometers and bio-informatics tools. The analysis of HRMS/MS fragmentations can also allow the automated and fast detection of derivatives related to a chemical core inside a metabolic cluster. Here, we will show how this strategy can be applied, with the example of 4 strains belonging to a new marine-derived *Penicillium* species selected for their antiproloferative activity against osteosarcoma cell lines. Investigations of their metabolome led to the isolation of several compounds among which a highly active and selective new metabolite. The use of specially developed LC-HRMS metabolomics tools allowed the detection in crude fungal extracts of some trace analogs of this lead compound for which structural proposals were done using HRMS/MS fragmentations modelisation. Their structures were then confirmed by semisynthesis which allowed the establishment of their structure-activity relationships against osteosarcoma. This exemple illustrates that the combination of dereplication, metabolomics and semi-synthesis is an interesting strategy for the elucidation of marine chemodiversity and the discovery of new cryptic analogs of selected compounds.

#### **Variable Temperature NMR J-Based Configurational Analysis of Flexible Acyclic Systems** 

**Jaime Rodríguez, Miriam Rega, Maria Isabel Nieto, Carlos Jiménez and Yuri Segade**

Departamento de Quimica Fundamental, Facultad de Ciencias, and Centro de Investigaciones Cientificas Avanzadas (CICA), Universidad da Coruña, 15071-A Coruña, Spain

NMR coupling constants and chemical shifts are the most used parameters to deduce both skeleton frameworks and three-dimensional arrangements in a molecule with an unknown relative stereochemistry. This difficult task becomes a challenge, and sometimes this is the bottleneck in the full characterization of a compound containing flexible moieties, such as polysubstituted open chains. Several approaches have becoming very popular in the last 15 years to study the relative stereochemistry in these acyclic compounds since a key publication published in 1999 by Murata and co-workers developed a new methodology for the organic structure analysis. This robust and logical method, known as J-based configurational analysis (JBCA), has been incorporated in the “spectroscopic toolbox” of all research groups involved in the organic-structure-elucidation field [1]. This methodology is based on the general use of the J(HH) and ^2,3^J(CH) and it has been applied in different 1,2- and 1,3-dimethine systems.^1^ However, there are some situations where the analysis becomes very complex because three different factors come in consideration: (a) The three staggered rotamers are present in some extend in the equilibrium, making all the coupling constants be averaged to medium values; (b) NOE measurements cannot discriminate the antiperiplana or the synclinal gauche-conformers equilibria in both erythro or threo configurations; (c) The medium values of the coupling constants are on the edge of the small/medium or medium/large Karplus-type curves, and therefore cannot be classified.Completing our previous studies, in this communication we want to present how all these cases can be solved using a methodology where, the mentioned three factors are diminished [2].

##### ***References*** 

1. Matsumori, N.; Kaneno, D.; Murata, M.; Nakamura, H.; Tachibana. K. Stereochemical Determination of Acyclic Structures Based on Carbon−Proton Spin-Coupling Constants. A Method of Configuration Analysis for Natural Products *J. Org. Chem.*
**1999**, *64*, 866–876.
2. Ardá, A.; Nieto, M.I.; Blanco, M.; Jiménez, C.; Rodríguez, J. Low-Temperature NMR J-Based Configurational Analysis of Flexible Acyclic Systems. *J. Org. Chem.*
**2010**, *75*, 7227–7232.

### **Biosynthesis of Marine Natural Products in Microbes** 

#### **Bioprospecting Microbial Mats for Novel Bioactive Compounds** 

**Silvia Cretoiu, Florianne Parel, Henk Bolhuis and Lucas Stal**

Royal Netherlands Institute for Sea Research, Yerseke, The Netherlands

Microbial mats are benthic communities of microorganisms, usually dominated by cyanobacteria, which develop at the interface of water and sediment, on rocks or on soil where they form microbial crusts. Microbial mats are globally distributed and can be found on coastal, dessert and Polar Regions, hypersaline environments and hot springs. Metagenomic investigation of coastal microbial mats showed that these small-scale ecosystems are repositories of a large number of genes from metabolic pathways relevant for biotechnology. Highly abundant genes involved in antibiotics and carbohydrates active enzymes synthesis indicate that microbial mats may be suitable as habitat model for isolation of novel organisms with biotechnological potential. A significant fraction of these genes were taxonomically affiliated to *Proteobacteria*,* Actinobacteria *and *Cyanobacteria*—bacterial groups well known for their usage in biotechnology. FP-7 EU project MaCuMBA is investigating this great potential and proposing new methodologies of assessing it for biotechnological purposes.

#### **Natural Product Biosynthesis in Uncultivated Sponge Symbionts** 

**Micheal C. Wilson and Jörn Piel**

Institute of Microbiology, Eidgenössische Technische Hochschule (ETH) Zürich, Zurich, Switzerland

Uncultivated filamentous bacteria from the candidate genus “*Entotheonella*” are common symbionts of geographically and taxonomically diverse marine sponges. Recently, we showed that members of the “*Entotheonella*” genus associated with the sponge *Theonella swinhoei *Y from Japan are the source of nearly all major metabolites previously isolated from the sponge. In our continued effort to study these bacteria, we discovered that “*Entotheonella*” associated with geographically and taxonomically distant sponges are also prolific producers of many known and cryptic natural products. Here we report the identification and characterization of natural product pathways from the genomes of “*Entotheonella*” from diverse sponges.

#### **Research on the Kenyan Marine Cyanobacterium Lyngbya Majuscula Opens New Frontiers in Marine Biodiscovery Efforts in Africa** 

**Thomas Dzeha**

University of Nizwa, Nizwa, Oman

**Abstract:** Cancer still remains the undisputed number one challenge in societal health care worldwide. A decade of research on the Kenyan marine cyanobacterium *Lyngbya majuscula* has revealed the geno and phenotypes for producing the anticancer modular cyclodepsipeptides homodolastatin 16 and dolastatin 16; and antanapeptin A in the *L. majuscula* genome. I report here important milestones and contributions of the research on the Kenyan *L. majuscula* for realizing a sustainable supply of these essentially important anticancer compounds for drug discovery. These findings highlight the urgent need for new frontiers in Marine Bio-discovery efforts in Africa.

**Keywords:** cancer; *Lyngbya majuscula*; modular cyclodepsipeptides; homodolastatin 16; dolastatin 16; antanapeptin A; genome

#### **Exploring Ripp Biosynthetic Pathways in a Quest for a Novel Macrocyclase with Improved Catalytic Properties** 

**Cristina-Nicoleta Alexandru-Crivac ^1,2^, Wael Houssen ^1,2^, Laurent Trembleau ^1^ and Marcel Jaspars ^1^**

^1^ Marine Biodiscovery Centre, Department of Chemistry, University of Aberdeen, Aberdeen AB24 3UE, Aberdeen, UK, 

^2^ Institute of Medical Sciences, University of Aberdeen, Aberdeen AB25 2ZD, Aberdeen, UK

Cyclic peptides are very important structures with high therapeutic potential, as they proved capable of interfering with challenging targets such as protein-protein interactions. Compared to their linear counterparts, cyclic peptides are more stable, have increased target-binding affinity, increased resistance to proteases and higher biological membrane permeability. We are exploring biosynthetic clusters from different RiPP classes to identify and engineer a novel macrocyclase with improved catalytic properties and wider substrate tolerance. In our laboratories, we currently use PatGmac macrocyclase from the patellamide pathway1, 2 to catalyse the N-C cyclization of linear peptides. However, PatGmac is a slow enzyme with limited capability to process peptides of 6–11 amino acids in length 3. The enzyme needs AYD signal at the C-terminal of the linear substrate, which should be preceded by a proline or a heterocycle to allow for cyclization. The latter will be incorporated in the final structure of the peptide substrate. We have also overexpressed and purified several homologues of PatGmac and tested their activity against various substrates. The successfully active macrocyclases will be combined with other enzymes from the cyanobactin biosynthetic pathways, with the aim of generating highly diverse macrocyclic scaffolds containing amino acids, enzymatically modified amino acids, non-natural amino acids and non-amino acid building blocks. Furthermore, understanding the structural differences between the various enzymes can elucidate the required structural motifs for improved activity and kinetics.

#### **Biosynthetic Studies of Secondary Metabolites Produced by the Sponge-Derived Fungus *Stachylidium* sp.** 

**Fayrouz El Maddah, Mamona Nazir, Stefan Kehraus and Gabriele M. König**

Institute for Pharmaceutical Biology, University of Bonn, Bonn, Germany

Marine fungi are in the focus of research as a source of structurally novel compounds. The marine-derived fungus *Stachylidium *sp. was isolated from the sponge *Callyspongia *sp. cf. *C*. *flammea*. Culture on a biomalt agar medium supplemented with sea salt yielded an ethylacetate extract which was chemically investigated and led to the isolation of a variety of metabolites including phthalides [1], phthalimidines [2], tyrosine derived compounds [3], and *N*-methylated cyclic peptides and diketopiperazines, with interesting bioactivities. The present study aims at determining the biogenetic origin of the isolated metabolites, employing classical isotope tracer experiments. Interesting structural features, such as the rare and intriguing amino acid *N*-methyl-3-(3-furyl)-alanine in the cyclic peptides, and the phthalide and phthalmidine skeleton of the marilones and marilines, respectively are targeted. Isotopic enrichment and ^13^C-^13^C coupling constants observed for *N*-methyl-3-(3-furyl)-alanine, after feeding of [U-^13^C] glycerol, [1-^13^C] glucose and [1-^13^C]phenylalanine, points to a shikimate-related pathway for its biosynthesis, including condensation of phosphoenolpyruvate and erythrose-4-phosphate to 3-deoxy-d-*arabino*-heptulosonic acid-7-phosphate (DAHP) as an intermediate. However, these experiments excluded the involvement of phenylalanine as a precursor. Feeding studies with [1-^13^C] sodium acetate proved the tetraketide nature of the phthalide and phthalimidine skeleton of the marilones and marilines, respectively. Concerning the starter unit involved in their biosyntheses, our results favour a methylated acetate starter unit over a propionate unit.

##### ***References*** 

1. Almeida, C.; Kehraus, S.; Prudêncio, M.; König, G.M. Marilones A–C, phthalides from the sponge-derived fungus *Stachylidium* sp. *Beilstein J. Org.Chem.*
**2011**, *7*, 1636–1642.
2. Almeida, C.; Hemberger, Y.; Schmitt, S.M.; Bouhired, S.; Natesan, L.; Kehraus, S.; Dimas, K.; Gütschow, M.; Bringmann, G.; König, G.M. Marilines A–C: Novel Phthalimidines from the Sponge-Derived Fungus *Stachylidium* sp. *Chem. Eur. J.*
**2012**, *18*, 8827–8834.
3. Almeida, C.; Part, N.; Bouhired, S.; Kehraus, S.; König, G.M*.* Stachylines A–D from the sponge-derived fungus *Stachylidium* sp. *J. Nat. Prod.*
**2011**, *74*, 21–25.

### **Marine Microbes** 

#### **Novel Members of the Phylum *Bacteroidetes*—A Potent Source of Bioactive Compounds** 

**Edda Olgudóttir ^1^, Sólveig K. Pétursdóttir ^1^, Sólveig K. Ólafsdóttir ^1^, Ólafur H. Friðjónsson ^1^, Beata Wawiernia ^1^, Elísabet E Guðmundsdóttir ^1^, Sigmar K. Stefánsson ^1,2^, Snædís H. Björnsdóttir ^1,2^ and Guðmundur Ó. Hreggviðsson ^1,2^**

^1^ Matís ohf, Reykjavík, Iceland,

^2^ Institute of Life and Environmental Sciences, University of Iceland, Reykjavík, Iceland

Several strains representing a novel taxonomic group were recently isolated from intertidal geothermal areas on both the SW and NW coast of Iceland. The strains derived from the vicinity of algal mats, from hot springs that emerge during low tide but are submerged at high tide. This environment is highly dynamic, where constant periodic fluctuations occur during tidal cycles involving for example steep gradients of temperature, salinity and mineral composition. Temperature gradients are manifested most clearly in the contrast between the hot geothermal water, emitted into the intertidal area from the hot springs, and the cold seawater. The strains were red-colored, grew well in marine broth and optimally at 60 °C. 16S rRNA gene analysis identified *Rhodothermus marinus *as their closest cultured relative based on 90% sequence similarity. This indicates that the strains represent a novel genus within the family *Rhodothermaceae *of the phylum *Bacteroidetes. *One strain, MAT 4553 was subjected to whole-genome shotgun sequencing using the 454 technology. The assembled sequence was of 4.6 Mb and a G + C content of 67%. A total of 3740 genes were identified. Numerous genes were found to encode carbohydrate-degrading enzymes. Several of these are of a potential commercial values and the corresponding genes have been cloned and expressed in *E. coli. *The genes encode enzymes such as pectinases, alginate lyases, poly-galacturonidases, rhamnosidases, galactosidases and a rhamnogalacturonan-esterase. This indicates that the novel strains are able to use algal sugars. In addition, operons were identified encoding genes that are potentially involved in the synthesis of secondary metabolites such as polyketides, bacteriocins and terpenes. The bioactive compounds of strain MAT 4553 are being investigated and the description of the strain as the type strain of a novel genus is currently underway.

#### **Actinomycete Metabolome Induction/Suppression with *N*-Acetylglucosamine** 

**Yousef Dashti ^1^, Tanja Grkovic ^1^, Usama Ramadan Abdelmohsen ^2^, Ute Hentschel ^2^ and Ronald J Quinn ^1^**

^1^ Eskitis Institute for Drug Discovery, Griffith University, Brisbane, QLD, Australia,

^2^ Department of Botany II, Julius-von-Sachs Institute for Biological Sciences, University of Würzburg, Würzburg, Germany

The metabolite profiles of three sponge-associated actinomycetes, namely *Micromonospora* sp. RV43, *Rhodococcus* sp. RV157 and* Actinokineospora* sp. EG49 were investigated after elicitation with *N*-acetylglucosamine. ^1^H NMR fingerprint methodology was utilized to study the differences in metabolic profiles of the bacterial extracts before and after elicitation. Our study found that the addition of *N*-acetylglucosamine modified the secondary metabolite profiles of all three investigated actinomycete isolates. *N*-acetylglucosamine induced the production of 3-formylindole and guaymasol in *Micromonospora* sp. RV43, the siderophore bacillibactin and surfactin antibiotics in *Rhodococcus* sp. RV157, and increased the production of minor metabolites actinosporins E-H in *Actinokineospora* sp. EG49. These results highlight the use of *N*-acetylglucosamine as an elicitor for the induction of silent biosynthetic pathways and for increasing the chemical diversity of microbial natural products.

#### **Diversity and Natural Products Repertoire of Marine Sponge-Associated Actinomycetes** 

**Usama Ramadan Abdelmohsen ^1^, Cheng Cheng ^1^, Lynsey Macintyre ^2^, RuAngelie Edrada-Ebel ^2^ and Ute Hentschel ^1^**

^1^ Julius-von-Sachs-Institute for Biological Sciences, University of Würzburg, Würzburg, Germany

^2^ Strathclyde Institute of Pharmacy and Biomedical Sciences, University of Strathclyde, Glascow, UK

Actinomycetes are known for their unprecedented ability to produce novel lead compounds of clinical and pharmaceutical importance. Our contribution focuses on the diversity, abundance, and methodological approaches targeting marine sponge-associated actinomycetes. Various approaches encompassing co-cultivation, elicitation experiments, bioassay-guided isolation, as well as -omics (genomics, metabolomics) were employed towards this goal. We will report on the following findings: (i) Chemical analysis resulted in the isolation of the novel cyclic lipopeptides, cyclodysidins A–D, from *Streptomyces* sp. RV15 associated with the marine sponge *Dysidea tupha*. From the same strain, one naphthoquinone derivative SF2446A2 was isolated and showed new antichlamydial and antischistosomal activities; (ii) Diazepinomicin, a dibenzodiazepine alkaloid, was isolated from strain *Micromonospora *sp. RV115 derived from the marine sponge *Aplysina aerophoba*. Using chemical as well as cell-based assays, a strong antioxidant potential of diazepinomicin was demonstrated. Moreover, diazepinomicin inhibited the proteases rhodesain and cathepsin L; (iii) A new *O*-glycosylated xanthone derivative, microluside A, was isolated from the broth culture of *Micrococcus* sp. EG45 cultivated from the Red Sea sponge *Spheciospongia vagabunda*. Microluside A exhibited antibacterial potential against *Enterococcus faecalis* JH212 and *Staphylococcus aureus* NCTC 8325; (iv) Using metabolomics to dereplicate the marine sponge-associated Actinokineospora sp. EG49 cultivated from the sponge *Spheciospongia vagabunda*, 20 compounds were identified, many of which are unknown. Bioassay-guided isolation of the same strain led to the isolation of new anti-trypanosomal and antioxidant angucyclines named actinosporins A–D. Interestingly, co-cultivation of the two sponge-derived actinomycetes,* Actinokineospora* sp. EG49 and *Nocardiopsis* sp. RV163, induced biosynthesis of three natural products that were not detected in the single culture of either microorganism. These were *N*-(2-hydroxyphenyl)-acetamide, 1,6-dihydroxyphenazine and 5a,6,11a,12-tetrahydro-5a,11a-dimethyl-1,4-benzoxazino[3,2-*b*][1,4]benzoxazine. The phenazine derivative was active against *Bacillus* sp. P25, Trypanosoma brucei and interestingly, against *Actinokineospora* sp. EG49. Our results highlight marine sponges as prolific resource for taxonomically novel and rare actinomycetes with potential for drug discovery.

#### **Actinobacteria from the South Pacific: A Bioprospection for Natural Bioproducts** 

**Beatriz Camara ^1^, Agustina Undabarrena ^1^, Fernanda Claverias ^1^, Valentina Gonzalez ^1^, Andres Cumsille ^1^, Myriam Gonzalez ^1^, Michael Seeger ^1^, Fabrizio Beltrametti ^2^ and Edward Moore ^3^**

^1^ Universidad Tecnica Federico Santa Maria, Valparaiso, Chile

^2^ Actygea s.r.l., Gerenzano, Italy

^3^ Culture Collection University of Goteborg, Goteborg, Sweden

Marine derived actinobacteria have demonstrated to be a source of a broad variety of secondary metabolites with diverse biological activities, including antibiotics, antifungal, antitumoral and extracellular enzymes, among others. Most of these metabolites are synthesized by complex metabolic pathways that involve polyketide synthases (PKS) and/or non-ribosomal peptide synthetases (NRPS). Recently, bioprospection in underexplored habitats has gained focus, since new taxa of marine actinobacteria can be found, and thereof, possible new metabolites. In this study, actinobacteria from marine sediments and sponges of three locations along different latitudes of the supratidal and subtidal coast of Chile (Chañaral de Aceituno Cove, III Region; Valparaiso Bay, V Region and Cumau Fjord, X Region) were isolated and its biotechnological potential was evaluated. Different culture conditions and selective media that enrich the growth of this phylum were used and approximately 300 bacterial strains were isolated. Comparative analysis of the 16S rRNA gene sequences led to identifying genetic affiliations of 27 known genera, belonging to 19 families. Also, a putative new genus was found, belonging to the Nocardiopsaceae family. The antimicrobial activity of representative isolates was evaluated against laboratory test strains (*Staphylococcus aureus*, *Listeria monocytogenes*, *Salmonella enterica*, *Escherichia coli* and *Pseudomonas aeruginosa*), demonstrating the ability of many isolates to inhibit the growth of gram-positive and/or gram-negative bacterial strains. Also, their ability to produce extracellular lipases and proteases was evaluated, and many isolates showed a significant activity. In addition, the presence of PKS and NRPS biosynthetic genes was evaluated, and most of the isolates were positive for at least one of the genes analyzed. This study shows a remarkable biodiversity of culturable actinobacteria, associated to marine environments along Chile. Our Chilean marine actinobacterial culture collection represents an important resource for the bioprospection of novel marine actinomycetes and its metabolites, which evidences their potential as producers of natural bioproducts.

#### **Metabolomics as a Tool to Search for New Potential Antibiotics from Mangrove Plant Avicennia Lanata and Its Endophytic Fungi** 

**Noor Wini Mazlan ^1,2^, Shan Hui Sim ^1^, Rothwelle Tate ^1^, Carol Clements ^1^ and RuAngelie Edrada-Ebel ^1^**

^1^ Strathclyde Institute of Pharmacy and Biomedical Sciences, University of Strathclyde, Glasgow, UK

^2^ Analytical Chemistry and Environment, School of Marine Science and Environment, Universiti Malaysia Terengganu, Kuala Terengganu, Terengganu, Malaysia

The discovery of new secondary metabolites from plants and endophytes has become more challenging in natural products chemistry. Bacteria and fungi interact within the host plant and stimulate competition for nutrients and spaces which is regarded as a major ecological factor that induces the production of bioactive secondary metabolites. In this study, the isolation of bioactive natural products was done on the crude organic extract of the mangrove plant Avicenna lanata collected from Terengganu, Malaysia using several high-throughput chromatographic techniques which yielded three new derivatives of 2,3-dihydro-1,4-naphthoquinone along with three known congeners and triterpenes. Meanwhile, three pure endophytic fungal strains were also isolated from *A. lanata*: *Aspergillus aculeatus* (leaf); *Lasiodiplodia theobromae* (stem) and *Fusarium *sp. (root). Prior to this study, metabolomics using high resolution mass spectrometry and NMR spectroscopy was applied to identify and optimize the production of bioactive secondary metabolites from the three strains cultivated in both solid rice and liquid culture media at 7, 15 and 30 days. The spectral data was processed utilizing the quantitative expression analysis software MZmine 2.10 coupled with the Antimarin database for dereplication studies. SIMCA P+ 13.0 was used to prove that the optimized models were statistically sound. The 15-day solid culture of *Fusarium *sp. yielded four naphthazarin-related 1,4-naphthoquinones, ergosterol peroxide and β-sitosterol. The 30-day *A. aculeatus* rice culture afforded the new compound, 2-(3,4dihydroxyphenyl)-*N*,*N*-dimethylacetamide, four simple phenolics and secalonic acid B, while the 15-day *L. theobromae* rice culture produced mellein along with its three derivatives. Structure elucidation of the isolated compounds was established using 1D and 2D-NMR and HRESI-MS. Aside from sterol compounds, all isolated compounds from *A. lanata *and its fungal showed significant anti-trypanosomal activity. This study proved that metabolomics is an essential tool to identify biomarkers from mangrove plants and endophytic fungi to target the isolation of potential anti-trypanosomal effective drugs.

## **Poster Session 1. Chemical Ecology** 

### ***Is the Specialized Metabolism Influenced by Environmental Conditions? The Case Study of the Mediterranean Sponge* Crambe crambe** 

**Eva Ternon ^1^, Erica Perino ^2^, Roberto Pronzato ^2^ and Olivier Thomas ^1^**

^1^ Nice Institute of Chemistry—PCRE UMR 7272 CNRS, University of Nice Sophia Antipolis, Nice, France

^2^ Department for the Study of the Territory and its Resources of the University, Genoa, Italy

In the age of marine biotechnology, research focusing on sponge secondary metabolites production for industrial purposes has strongly increased. Indeed, sponges are marine animals that produce a large array of bioactive compounds. However, industrial production requires a large amount of raw material, and sponges are characterized by low-growth, in restricted areas. In order to supply large quantities of secondary metabolites, sponges have been cultivated all around the world and the most accurate environmental conditions for this purpose have been investigated. In the Mediterranean Sea, the encrusting sponge *Crambe crambe* has been showed to produce two bioactive families of metabolites, the crambescins and the crambescidins. In the frame of the European project BAMMBO, farming of *C. crambe* was conducted in two areas of the Northwestern Mediterranean Sea that differ by their environmental conditions. In the meantime, wild sponge specimens were collected close to the farming area. Additionally, *ex situ* cultures of this sponge species were set up in aquaria during 30 days, under a constant flow and under various environmental conditions. The variation in the specialized metabolome in relation with their living conditions was assessed by both targeted and un-targeted metabolomics approaches, using UHPLC-HRMS (QTof). Data showed that both seasons and sampling area are factors affecting the specialized metabolome. On the opposite, no significant correlation was found between the mode of growth (farming, *ex situ* cultures or wild) and any modification of the nature of the specialized metabolome.

### ***Comparison Studies on Fatty Acid Profiles of Four Seaweeds* Sargassum *in China*** 

**Hongbing Liu and Zhen Chen**

Institute of Marine Food and Drugs, School of Medicine and Pharmacy, Ocean University of China, Qingdao, Shandong, China

*Sargassum* is a genus of approximately 250 species in Sargassaceae and is geographically widespread in all tropical and temperate oceans. *S. fusiforme*, *S. pallidum*, *S. honeri*, and *S. thunbergii* are mainly distributed in the coast of the North Pacific Ocean with an abundant biomass in China. As important economic seaweeds, these species have been used as nutritional foods and herbal medicine for treating hyperlipidemia, hypertension, heart disease, inflammatory diseases, and cancer in China for thousands of years. The chemical differences between these medicinal species have not been systematically investigated. In the present study, fatty acid profiles of *S. fusiforme*, *S. pallidum*, *S. honeri*, and *S. thunbergii* were established based on GC-MS, respectively (totally 87 batches). By means of principal component analysis (PCA), these *Sargassum* species could be well differentiated from each other. It was alga species that dominated the chemical diversity other than environmental features such as growing region and water temperature in *Sargassum.* For *S. fusiforme* and *S. pallidum*, which are both listed in the Chinese Pharmacopoeia, a further comparison indicated that six potential key fatty acids (C16:0, C18:4 *n*-3, C20:2 *n*-6, C20:4 *n*-6, C20:5 *n*-3, and C22:1 *n*-9) could be employed to distinguish them. Meanwhile, *S. fusiforme *contained higher unsaturation index (UI) and lower *n*-6/*n*-3 ration than* S. pallidum*, which hinted that the former might be more beneficial for anti-cardiovascular disease.

### ***Screening of Indonesian Macroalgae by Their Total Phenolic Contents and Antioxidant Activities: The Interest of Brown Seaweeds as a Source of Active Phlorotannins*** 

**Nur Azmi Ratna Setyawidati ^1,2^, Klervi Le Lann ^1^, Alizé Bagot ^1^, Ita Widowati ^3^ and Valérie Stiger-Pouvreau ^1^**

^1^ LEMAR UMR 6539 UBO CNRS Ifremer IRD, European Institute of Marine Studies (IUEM), Université de Bretagne Occidentale (UBO), European University of Brittany (UEB), Technopôle Brest-Iroise, Plouzané, France

^2^ Center for Marine and Fisheries Technology, Agency for Marine Affairs and Fisheries Research and Development, Minister of Marine Affairs and Fisheries, Jakarta, Indonesia

^3^ Faculty of Fisheries and Marine Sciences, Diponegoro University, Semarang, Indonesia

Biodiversity of macroalgae are considered as an economic importance in Indonesia. Within the framework of the international project called INDESO, we assessed the potential and the added-value of natural populations of abundant Indonesian seaweeds. We investigated the chemical composition of 17 common red, green and brown seaweeds from two bays, Malasoro and Ekas, sampled during dry and wet seasons. The conventional solid/liquid extraction was conducted using two solvent mixtures: ethanol 75% (EtOH-water 75:25) and 25% (EtOH-water 25:75). All of the crude extracts were tested for their free radical scavenging activity; using the DPPH (2,2-diphenil-1-picrylhydrazyl). From these procedures; the highest TPC and strongest DPPH were showed in the ethanolic extraction (EtOH-water 75:25) of brown algae from Ekas Bay, *Padina australis* and *Sargassum* sp., and the aqueous (EtOH-water 25:75) of two brown algae from Malasoro bay, *Sargassum *sp. and *Turbinaria conoïdes*, collected during the dry season. From the four interesting brown algae, two different fractions were obtained by liquid/liquid purification procedure: (i) an ethyl acetate fraction (AE) and (ii) an aqueous fraction (AQ). We determined the phenolic content from the crude extraction and the two purified fractions, AE and AQ, together with their anti-scavenging activities: DPPH and ferric reducing antioxidant power (FRAP) assay. The results of the screening and the activity of purified fractions were discussed and could be indicated encouraging perspectives for the use of Indonesian brown seaweeds. These seaweeds would be a valuable source of antioxidant natural compounds for Indonesia. This work was financed with the support of the INDESO project (2013–2016), funded by the French Development Agency (AFD), being sponsored and coordinated by the Indonesian Ministry of Marine Affairs and Fisheries (MMAF).

### ***Chemical Characterization, Anti-Microbial Inhibitory and Cytotoxic Activities of Crude Compounds from Mediterranean Ascidian Styela Plicata (Lesur, 1823)*** 

**Satheesh Kumar Palanisamy ^1^, Salvatore Giacobbe ^1^, Ronald J. Quinn ^2^, Donatella Del Bufalo ^3^, Daniela Trisciuoglio ^3^, Angelo Marino ^1^, Salvatore Cuzzocrea ^1^ and Antonello Mai ^4^**

^1^ Department of Biological and Environmental Sciences, University of Messina, Messina 98166, Italy

^2^ Eskitis Institute for Drug Discovery, Griffith University, Brisbane, Queensland 4111, Australia

^3^ Istituto Nationale Tumori Regina Elena, Rome 00144, Italy

^4^ Pasteur Institute, Cenci-Bolognetti Foundation, Department of Drug Chemistry and Technologies, Sapienza University of Rome, Rome 00185, Italy

Marine ascidians are sessile organisms with a great ability to synthesize bioactive substances; they are well-known reservoir for novel Marine Natural Products (MNPs). The primary objective of the present study includes documentation of the taxonomy and distribution of ascidian fauna in Mediterranean Sea-Messina, to identify drug-like molecules from the ascidian Styela plicata and finds their pharmacological properties. The Faro Lake, Messina at the south-end of the Italy has a great number of ascidian fauna. In the present study, we reported 15 ascidian species in 7 family and 11 genera, primarily from coastal lake, Ionian Sea, Messona. Maximum 6 species were reported from the family Styelidae, 2 species from the family Aascidiadae, Ascidiiae and Didminidae and remaining others. The chemical characterization of ascidian crude extract was determined by FTIR, LC-MS and 1H-NMR. The crude extracts of *S. plicata* showed remarkable anti-microbial activity against B831—*Pseudomonas *sp. (16 mm) and B7—*Burkholderia mallei* (12 mm). The crude compounds were showed modest cytotoxicity against various tumour cell lines human epithelial kidney cells (HEK 293 Phoenix cells), HeLa-cervical cancer and HT1080 human fibrosarcoma cells with IC50 values of (93, 139 and >500 µm/mL) repectively. Pyrimidine metabolites, dihydro-5-methylpyrimidine-2,4(1*H*,3*H*)-dione was isolated first time from *S. plicata*. Their structure was determined by 2D-NMR techniques. Nevertheless, further studies are recommended to verify which fraction is responsible for the biological activity of the whole extract. 

**Keywords:** ascidians; cell; crude extract; cytotoxic; FTIR; LC-MS; NMR

### **Marine Toxins and Bioassays** 

#### **Discovery of Peptide Toxins in the Bootlace Worm, the World’s Longest Animal** 

**H.S Andersson ^1^, E. Jacobsson ^2^, C. Eriksson ^2^, K.J. Rosengren ^3^, P. Andrén ^4^, M. Strand ^5,6^ and U. Göransson ^2^**

^1^ Linnaeus University Centre for Biomaterials Chemistry, Department of Chemistry and Biomedical Sciences, Linnaeus University, Kalmar, Sweden

^2^ Division of Pharmacognosy, Department of Medicinal Chemistry, Uppsala University, Uppsala, Sweden

^3^ School of Biomedical Sciences, The University of Queensland, Brisbane, Australia

^4^ Department of Pharmaceutical Biosciences, Uppsala University, Uppsala, Sweden

^5^ Department of Biological and Environmental Sciences, University of Gothenburg, Gothenberg, Sweden

^6^ Swedish Species Information Centre, Swedish University of Agricultural Sciences, Uppsala, Sweden

Nemerteans (ribbon worms) are marine predators, which capture their prey using a proboscis containing a mixture of toxins which brings on rapid paralysis [1]. In addition, the epidermis of nemerteans contains a thick mucus of similar toxic constitution. Although the presence of low-molecular weight toxins such as anabasine and tetrodotoxin have been described previously, the presence of peptide based toxins in nemerteans, with one notable exception [2] have not been reported. We have now found such toxins in the mucus of the bootlace worm (*Lineus longissimus*), a spectacular species that can reach up to 50 meters in length. We have been able to confirm the expression of peptide toxins from this worm via LC-MS and MALDI imaging. Two peptides have been isolated and the peptide sequences have been elucidated; these peptides contain three disulfides and represent a novel family of nemertean toxins. Purified extract of one of these peptides was shown to produce rapid cramping followed by death in shore crabs. In addition, a third peptide, with strong resemblance to the only peptide toxin previously characterized from nemerteans, Neurotoxin B-IV, a 55 aa peptide, has been discovered. In addition, we have sequenced the transcriptome of the bootlace worm and obtained the transcriptomes of a series of other nemerteans, presently under investigation. In this presentation, we intend to discuss these findings and their implications further.

##### ***References*** 

1. Kem, W.R. Structure and activity of Nemertine Toxins. *Integr. Comp. Biol.*
**1985**, *25*, 99–111.
2. Kem, W.R. Purification and characterization of a new family of polypeptide neurotoxins from the heteronemertine *Cerebratulus lacteus* (Leidy). *J. Biol. Chem.*
**1976**, *251*, 4184–4192.

#### **Anti-Tumor and Anti-Angiogenic Activity of the Cyanobacterial Metabolite Coibamide A** 

**Jeffrey Serrill, Xuemei Wan, Adam Alani, Kerry McPhail and Jane Ishmael**

Oregon State University, Corvallis, OR, USA

Coibamide A is an *N*-methyl-stabilized depsipeptide that was isolated from a marine cyanobacterium collected using SCUBA from the Coiba National Park as part of the International Cooperative Biodiversity Groups (ICBG) program based in Panama. Previous testing of coibamide A in the National Cancer Institute *in vitro* 60 cancer cell line panel revealed an unmatched selectivity profile indicative of a unique mechanism of action. Coibamide A displayed sub-nanomolar potency as a growth inhibitory agent and good histological selectivity for several solid tumor cell types including CNS, breast, colon and ovarian cancer. Although the cellular target of this structure is unknown, we have determined that coibamide A induces cell-cycle arrest at the G1 stage and is a potent and effective cytotoxin with the ability to trigger apoptosis or alternate cell death signaling in human cancer cells. Coibamide A also induces a rapid, mTOR-independent autophagy response that is not required for cell death but occurs as a cell stress response to treatment. We report that coibamide A shows promising *in vivo* efficacy in a subcutaneous mouse model of human glioblastoma; treated animals showed significant decreases in tumor growth and increased survival relative to control animals. Coibamide A also displays potent anti-angiogenic activity *in vitro* and effectively inhibits the ability of human umbilical vein endothelial cells (HUVECs) to proliferate, migrate, and form three-dimensional networks. Coibamide A selectively decreases the expression of several candidate membrane and secreted proteins that are particularly important for angiogenesis, such as VEGF-A, VEGF receptor 2 and VCAM-1. Coibamide A remains a lead structure in natural product drug discovery by virtue of its ability to modulate the growth and development of tumors in a multi-faceted manner through its effects on both cancer and endothelial cell types.

#### **Seabiotech: From Sea-Bed to Test-Bed: Harvesting the Potential of Marine Biodiversity for Industrial Biotechnology: An Overview of the Advances in Year 2** 

**Lynsey MacIntyre, Mariana Fazenda, Tong Zhang, Linda Harvey, Carol Clements, Louise Young, Grainne Abbott, Christina Viegelmann, Catherine Dowdells, RuAngelie Edrada-Ebel and Brian McNeil**

University of Strathclyde, Glasgow, Strathclyde, UK

Discover the progress that an interdisciplinary consortium of European Scientists has made in their explorations of marine microbial diversity to discover new products with the potential for development as antibiotics anti-infectives and anti-cancer treatments; and the developments for sustainable manufacture of these products on an industrial scale. The advances made in the second year of the project will be presented: illustrating the biodiversity and number of samples collected; the preliminary results obtained from a comprehensive screening campaign; the use of metabolomics as a tool to monitor secondary metabolite production under different culture conditions; and the scale-up to bio-fermenter level. SeaBioTech is an SME-driven EU-FP7 project designed to create innovative marine biodiscovery pipelines as a means to convert the potential of marine biotechnology into novel industrial products for the pharmaceutical, cosmetic, food and industrial chemistry sectors. The program brings together experts in biology, genomics, natural product chemistry, bioactivity testing, industrial bioprocessing, legal aspects, market analysis and knowledge-exchange. SeaBioTech targets novel marine endosymbiotic bacteria from unique and untapped habitats, including geothermal intertidal biotopes in Iceland, hydrothermal vent fields and deep sea oligotrophic basins of the Easter Mediterranean Sea and Iceland as well as areas of the Scottish coasts that are likely highly productive sources of new bioactive compounds. The sampling process utilises metagenomics to ensure the quality of marine resources for further industrial development. SeaBioTech combines metabolomics with systems biology and functional bioassays to increase the ability to uncover positive hits with a cheaper faster approach: an affordable innovative and efficient method to separate, elucidate the structure and identify bioactive metabolites; and through well controlled metabolic engineering increase their yield at lab and industrial scales. UNCBD Nagoya protocol compliant. The SeaBioTech project is funded by the European Commission within its FP7 Programme, under the thematic area KBBE.2012.3.2-01 Grant Number 311932.

#### **An Integrated Lead Discovery Programme within Seabiotech to Address Major Parasitic Infections in Aquaculture** 

**Christer Wiik-Nielsen ^1^, Louise Young ^2^, Laura Stucchi ^3^, Lynsey MacIntyre ^2^, Grainne Abbott ^2^, Elin Aksnes ^1^, Ruangelie Edrada-Ebel ^2^, Daniele Carettoni ^3^, Michele Stanley ^4^, Cheng Cheng ^5^, Ute Hentschel Humeida ^5^ and John Day ^4^**

^1^ Pharmaq AS, Oslo, Norway

^2^ Strathclyde Institute of Pharmacy and Biomedical Sciences, University of Strathclyde, Glasgow, UK

^3^ Axxam SpA, Bresso (Milan), Italy

^4^ The Scottish Association for Marine Science, Scottish Marine Institute, Oban, UK

^5^ Department of Botany II, Julius-von-Sachs Institute for Biological Sciences, University of Würzburg, Würzburg, Germany

A limiting factor for the identification of novel antiparasitics in aquaculture is the lack of high-throughput assays. Indeed, low-throughput phenotypic assays on living parasites are mainly available, making them incompatible to assist the screening of large sample collections. To enable the drug discovery efforts aimed at counteracting the infesting parasite sea lice, a joint collaborative program within SeaBioTech was implemented between Strathclyde University, the Scottish Association for Marine Science (SAMS), University of Würzburg, Axxam and Pharmaq. The strategy relies on the identification of high-throughput assays as pre-selection tools for phenotypic screening. The pre-selection assays were identified according to two complementary principles. First, functional assays developed on an orthologous sodium channel and on eel acetyl-cholinesterase were made available by Axxam and Strathclyde University, respectively. These assays measures the activity of validated targets, whose function is impaired by antiparasitics used in industrial settings. In the second strategy, an array of cell-based assays was validated by Axxam for their sensitivity to reference compounds used as antiparasitic agents in aquaculture. As a result, four preselection assays were identified in total. To date, over 650 extracts of marine origin assembled along the SeaBioTech program were screened at Axxam and Strathclyde University on the four preselection assays. In total, 135 extracts were identified as candidates for screening on sea lice at Pharmaq. Two extracts obtained by SAMS and by the University of Würzburg and identified by Axxam for their antagonistic activity on two preselection assays were confirmed to display high parasiticidal activity against sea lice. The two extracts displayed a selective activity profile on 26 functional assays used within SeaBioTech, and preliminary dereplication data generated by Strathclyde University indicated a potentially interesting chemistry profile. The SeaBioTech project is funded by the European Commission within its FP7 Programme, under the thematic area KBBE.2012.3.2-01, Grant Number 311932.

#### **Seabiotech: From Sea-Bed to Test-Bed: Harvesting the Potential of Marine Biodiversity for Industrial Biotechnology** 

**RuAngelie Edrada-Ebel1 ^1^, Lynsey MacIntyre ^1^, Mariana Fazenda ^1^, Tong Zhang ^1^, Carol Clements ^2^, Louise Young ^2^, Grainne Abbott ^2^, Linda Harvey ^1^, Alan Harvey ^2^ and Brian McNeil ^1^**

^1^ Strathclyde Institute of Pharmacy and Biomedical Sciences, University of Strathclyde, 161 Cathedral Street, Glasgow, UK

^2^ Strathclyde Innovations in Drug Research (S.I.D.R), University of Strathclyde, 161 Cathedral Street, Glasgow, UK

SeaBioTech is an EU-FP7 project designed and driven by SMEs to create innovative marine biodiscovery pipelines as a means to convert the potential of marine biotechnology into novel industrial products for the pharmaceutical (human and aquaculture), cosmetic, functional food and industrial chemistry sectors. To achieve its goals, SeaBioTech brings together leading experts in biology, genomics, natural product chemistry, bioactivity testing, industrial bioprocessing, legal aspects, market analysis and knowledge-exchange. SeaBioTech targets novel marine endosymbiotic bacteria from unique and previously untapped habitats, including geothermal intertidal biotopes in Iceland, hydrothermal vent fields and deep sea oligotrophic basins of the Eastern Mediterranean Sea and unsampled areas of Scottish coasts that are likely to be highly productive sources of new bioactive compounds. This poster describes the first year’s activity of the SeaBioTech project which resulted in a robust, validated workflow suitable for evaluating unexplored activity of marine samples. UNCBD Nagoya protocol compliant. The SeaBioTech project is funded by the European Commission within its FP7 Programme, under the thematic area KBBE.2012.3.2-01 with Grant Number 311932.

#### **Microalgal Natural Products with Bioactivities Relevant for Human Health** 

**Chiara Lauritano ^1^, Jeanette H. Andersen ^2^, Espen Hansen ^2^, Marte Albrigtsen ^2^, Laura Escalera ^1^, Francesco Esposito ^1^, Kirsti Helland ^2^, Kine Ø. Hanssen ^2^, Giovanna Romano ^1^ and Adrianna Ianora ^1^**

^1^ Stazione Zoologica Anton Dohrn, Napoli, Italy

^2^ Marbio, University of Tromsø, Tromsø, Norway

The EU project PharmaSea is a large consortium of 24 partners whose aim is to discover new bioactive compounds from marine microorganisms for the treatment of various human diseases. Marine microalgae have been shown to be valuable sources of novel biologically active molecules for applications in the food industry (additives, pigments, vitamins and colorants) as well as in the pharmaceutical and cosmetic sectors. For the PharmaSea project we tested crude extracts of different microalgal classes for possible antioxidant, anti-inflammatory, anticancer, anti-diabetes, antibacterial and anti-biofilm activities. Previous studies have shown that many microalgae produce defense metabolites with potentially interesting biotechnological applications when they are grown in stressful conditions. Hence, selected microalgae were grown under different culture conditions in order to identify those that produced higher amounts of the compounds of interest. Our results indicate that various species belonging to the Bacillariophyceae and Coscinodiscophyceae displayed anti-inflammatory, anticancer (blocking human melanoma cell proliferation) and anti-biofilm (against the bacteria *Staphylococcus epidermidis*) activities. We also observed that different clones and species grown in nutrient starvation conditions had different effects. An additional dereplication step has identified 10-Hydroxyphaeophorbide A in various Bacillariophyceae as possibly responsible for the observed anti-inflammatory activity, while Phaeophorbide A and Phaeophytin A have been identified in Coscinodiscophyceae as the possible agents for anticancer and anti-biofilm activities, respectively. The tested extracts will be further studied for possible isolation and identification of new compounds with these bioactivities. In addition, RNA-seq of the bioactive species is currently underway in order to identify the enzymatic pathways involved in the production of the metabolites of interest.

#### **Inhibitory Activity against the Chikungunya Virus of Extracts from the Soft Coral *Lobophytum microlobulatum*** 

**Yik Sin Chan, Nam Weng Sit and Kong Soo Khoo**

Faculty of Science, Universiti Tunku Abdul Rahman, Kampar, Perak, Malaysia

Chikungunya fever is an arboviral disease transmitted by *Aedes* mosquitoes. It has resulted in epidemics of the disease in tropical countries in the Indian Ocean and South East Asian regions. This study aims to investigate the anti-chikungunya virus activity of extracts from a soft coral, *Lobophytum microlobulatum*, collected from Malaysian waters. Following lyophilization and grinding to powder form, the sample was subjected to sequential solvent extraction using hexane, chloroform, ethyl acetate, ethanol, methanol and distilled water in order to extract bioactive compounds. The antiviral activity was evaluated in three different modes (pre-, concurrent and post-treatment) using monkey kidney epithelial (Vero) cells infected with the virus (multiplicity of infection = 1). The cell viability was determined by Neutral Red uptake assay. Strong antiviral activity (cell viability 70%) was observed only in the concurrent mode whereby the hexane and ethanol extracts resulted in cell viabilities of 73.3% ± 2.6% and 79.2% ± 0.9% respectively (mean ± SD; *n* = 3). The corresponding mean 50% effective concentrations (EC_50_) for the extracts were 14.2 ± 0.2 and 115.3 ± 1.2 µg/mL respectively. The chloroform extract showed moderate antiviral activity (cell viability 31%–69%) in the concurrent mode (cell viability = 57.9% ± 0.8%) as well as in the pre-treatment mode (cell viability = 51.2% ± 2.4%). Neither strong nor moderate antiviral activity was shown in the post-treatment mode. The two extracts with strong antiviral activity were selected for analysis of viral load in the assay using quantitative Reverse Transcriptase-Polymerase Chain Reaction (qRT-PCR). The viral loads in the assay treated with the hexane (20 mg/mL) and ethanol (320 mg/mL) extracts were 5.62 × 10^6^ ± 0.11 × 10^6^ and 4.97 × 10^7^ ± 0.67 × 10^7^ copies/mL respectively, indicating a reduction of 99.6% and 96.3% respectively compared to the virus control. The ethanol extract appears to hold the most promise for further characterization of active compounds as it possessed greater selectivity index (SI > 5.6) compared to the hexane extract (SI = 2.1).

#### **Neuroprotective Effect of Seaweeds Extracts against Dopamine-Induce Cell Death in Human Neuroblastoma Sh-Sy5y Cells** 

**Joana Silva, Celso Alves, Susete Pinteus, André Horta, Susana Mendes and Rui Pedrosa**

MARE—Marine and Environmental Sciences Centre, Polytechnic Institute of Leiria, Peniche, Leiria, Portugal

The Parkinson Disease (PD) is a neurodegenerative disease of the central nervous system that results in the depletion of dopamine production cells in a specified region of the brain, affecting about 1% of the world population. Although the causes of PD pathogenesis remains incomplete, some evidences from human and animal studies has suggested that oxidative stress is an important mediator in its pathogenesis. The aim of this study was to evaluate the protective effects of seaweeds extracts on dopamine (DA)-induced neurotoxicity in the human neuroblastoma cell line SH-SY5Y, as well the associated intracellular signaling pathways. Cell viability studies were assessed by (3-(4,5-dimethylthiazol-2yl)-2,5-diphenyltetrazolium bromide) MTT assay and the intracellular signaling pathways associated to oxidative stress induced by DA: H_2_O_2_ generation, mitochondrial membrane potential and caspase-3 activity. Exposure of SH-SY5Y cells to 30–3000 µM of DA caused significant effects on the reduction of cell viability, in a concentration (24 h) and time-dependent manner (6 h, 12 h, 24 h, 48 h). The data suggest that the cell death induced by DA was mediated by the increase of H_2_O_2_ production, depolarization of mitochondrial membrane potential and the increase of caspase-3 activity. The treatment with seaweeds extracts (1 mg/mL; 24 h) evidenced a neuroprotection effect on toxicity induced by DA (1000 µM; 24 h), namely by recovering cell viability between 10% and 25%. The protective effect obtained by seaweeds extracts against DA-induced toxicity could be mediated by an anti-apoptotic effect (mitochondrial protection and decrease of caspase-3 activity). These results suggest that seaweeds can be a promising source of new compounds with neuroprotective potential.

#### **Gambierone, a Ladder-Shaped Polyether from the Dinoflagellate *Gambierdiscus belizeanus*** 

**Kevin Calabro ^1^, Inés Rodrìguez ^2^, Grégory Genta-jouve ^3^, Carmen Alfonso ^4^, Eva Alonso ^2^, Jon Sànchez ^2^, Amparo Alfonso ^2^, Luis Botana ^2^ and Olivier Thomas ^1^**

^1^ Institut de Chimie de Nice, Université Nice Sophia Antipolis, UMR 7272 CNRS, Faculté des Sciences, Nice 06108, France

^2^ Departamento de Farmacología, Universidad de Santiago de Compostela, Facultad de Veterinaria, Lugo 27002, Spain

^3^ Laboratoire de Pharmacognosie et de Chimie des Substances Naturelles, Université Paris Descartes, COMETE UMR 8638 CNRS, Paris 75006, France

^4^ Laboratario CIFGA S.A., Plaza de Santo Domingo, n° 20, 5ª Planta, Lugo 27001, Spain

Ciguatoxin (CTX) intoxications are a serious and global public health issue and the identification of compounds associated to this toxin group is critical but usually highly challenging due to structural complexity and low amount available. We report herein the isolation, structure elucidation and biological activity of gambierone (**1**), a new natural product from the dinoflagellate *Gambierdiscus belizeanus*. This compound exhibits unprecedented polyether skeleton and right-hand side-chain. Its relative configuration was fully determined by interpretation of NOESY experiment and comparison between experimental and theoretical NMR data. Although the succession of cycles has no chemical similarity with CTXs, **1** has molecular formula and biological activity similar to CTX-3C, although lower in intensity.

#### **Antibacterial Compounds from Indonesian Rhodophyta against *Aeromonas hydrophilla* and *Vibrio* sp.** 

**Adhika P. A. Wijnana, Mamluatul Hikmah, Amirul Mu'minin, Noer Kasanah, Triyanto and Alim Isnansetya**

Fakulty of Agriculture, Gadjah Mada University, Yogyakarta, Indonesia

Rhodophyta have potential as high source of bioactivity secondary metabolites. The study was conducted to screen the activity of secondary metabolites from three *Gracilaria edulis*, *Gracilaria arcuata*, and *Gelidium spinosum* against two fish pathogenic bacteria (*Aeromonas hydrophila *and *Vibrio* sp.). The Rhodophytas were collected from Drini beach, Yogyakarta, Indonesia. For each species, 100 g dried samples were extracted using ethyl acetate. Bioassay guided fractionation was conducted on column chromatography using silica gel and step gradient eluent. Bioactivity compounds were identified using GC-MS and LC-MS in conjunction with database. The results showed that three fractions from *G. arcuata*, five fractions from *G. edulis*, and two fractions from *G. spinosum* have bioactive compounds against *Aeromonas hydrophila*, while only two fractions from *G. arcuata* and *G. spinosum *have bioactive compounds against *Vibrio *sp. The MIC concentrations were around 0.625 μg/μL to 2.5 μg/μL. Several chemical componens that had been identified were terpenes, phenolic, steroid, and fatty acid compounds.

#### **Analysis of Iodine in *Fucus serratus* Seaweed Bathwater and Its Potential for Uptake by the Body** 

**Tarha Westby, Aodhmar Cadogan and Geraldine Duignan**

Institute of Technology Sligo, Sligo, Ireland

Iodine, found in seaweed, is a vital micronutrient for normal growth, development and function. The levels of iodine in seaweed vary depending on species, locality and season and can account for up to 1.2% of the dry weight of some seaweed. The tradition of seaweed baths has been revived recently in Ireland and it is suggested that there is a considerable uptake of minerals by the body during such treatments. Traditional seaweed baths are prepared using fresh water and seawater along with the seaweed *Fucus serratus*. Simulated seaweed bathwater samples were prepared and stored weekly over 12 months. These were analysed using the classical spectrophotometric Sandell-Kolthoff (SK) assay in order to establish the seasonal variation in iodine levels. Results show that the concentration of total iodine ranges from 0.8 to 14 ppm. Results of pH analysis of the bathwater indicate an acidic environment (pH 5.8). The acidity of the bathwater affects the species of iodine present where iodine gas (I_2_) and iodate (IO_3_^−^) predominate over iodide (I^−^). As approximately 90% of iodine present in the body is excreted in the urine, analysis of the urinary iodine concentration (UIC) of bathers’ samples collected immediately prior to bathing and following bathing over 32 h was carried out to establish if there is an increase in UIC as a result of taking a seaweed bath. Almost all samples, from bathers and controls, analysed by the SK assay demonstrate an increase in UIC following a seaweed bath. The control subjects showed a spike in UIC despite not immersing in the bathwater but sitting beside it inhaling iodine gas only. This suggests that iodine gas contributes greatly to the increase in UIC for all subjects as there was no possibility of dermal absorption for the control.

#### **Oxidative Stress Condition Induced by H2O2 Is Blunted by *Fucus spiralis* Fractions** 

**Joana Silva ^1^, Susete Pinteus ^1^, Celso Alves ^1^, André Horta ^1^, Olivier Thomas ^2^ and Rui Pedrosa ^1^**

^1^ MARE—Marine and Environmental Sciences Centre, Polytechnic Institute of Leiria, Peniche, Leiria, Portugal

^2^ Institut de Chimie de Nice, University of NiceSophia Antipolis, Nice, France

Oxidative stress is one of the main causes for the development of diseases with huge impact in world societies such as cardiovascular diseases, neurodegenerative diseases, metabolic syndrome, cancer, etc. In line with these, becomes of extremely importance to find new molecules with capacity to inhibit, delay or prevent oxidative stress situations. During the last decades marine organisms have demonstrated the ability to produce molecules with high antioxidant properties, of which brown algae have shown high potential. *Fucus spiralis* (spiral wrack) is a brown algae (Phaeophyceae), living on the Atlantic coasts of Europe and North America. Previous studies of our research group found high antioxidant potential in methanolic extracts of *Fucus spiralis*, collected on the Peniche coast. In order to go further to the purification of the antioxidant compounds it was performed a Vacuum Liquid Chromatography (VLC) with the following solvent system: 100% H_2_O (F1); 1:1 H_2_O:CH_3_OH (F2); 100% CH_3_OH (F3); 3:1 CH_3_OH: CH_2_Cl_2_ (F4) and 100% CH_2_Cl_2_ (F5) in a C18 column. All fractions (1 mg·mL^−1^; 24 h) were tested for anti-oxidative stress potential in Human breast adenocarcinoma model (MCF-7 cells). The cytotoxicity was evaluated by the MTT method in a presence or in absence of an induced oxidative stress situation promoted by H_2_O_2_ (0.2 mM; 24 h). Finally, in order to understand possible mechanisms of action of the active compounds, the mitochondrial membrane potential (MMP) and the caspase 9 activity were evaluated. The VLC fraction did not revealed cytotoxicity. All fractions revealed capacity to revert an oxidative stress condition by the following potency order F4 > F2 > F3 > F5 > F1. Almost all the fractions reduced the cell’s membrane depolarization and suggested anti-apoptotic mechanisms attesting the protective effect against hydrogen peroxide toxicity. It is concluded that Fucus spiralis fraction has high antioxidant activity able to inhibit the MCF-7 oxidative stress condition induced by H_2_O_2._

#### **Bioprospecting Marine Microalgae, New Sources of Bioactive Compounds** 

**Caterina Rodríguez de Vera ^1,2^, Guillermo Díaz Crespín ^1,2^, Antonio Hernández Daranas ^1,3^, José Javier Fernández ^1,2^, María Luisa Souto ^1,2^, Päivi Tammela ^4^, Heiko Rischer ^5^, Christian D. Muller ^6,7^ and Manuel Norte ^1,2^**

^1^ University Institute of Bio-Organic Chemistry “Antonio González”, Center for Biomedical Research of the Canary Islands (CIBICAN), University of La Laguna, La Laguna, 38206 Tenerife, Spain

^2^ Departament of Organic Chemistry, Univesity of La Laguna, La Laguna, 38206 Tenerife, Spain

^3^ Departament of Chemical Engineering and Pharmaceutical Technology, University of La Laguna, La Laguna, 38206 Tenerife, Spain

^4^ Centre for Drug Research, Division of Pharmaceutical Biosciences, Faculty of Pharmacy, University of Helsinki, FI-00014 Helsinki, Finland

^5^ VTT Technical Research Centre of Finland, Fi-02044 Espoo, Finland

^6^ Laboratoire d’Innovation Thérapeutique, Faculté de Pharmacie, Université de Strasbourg, 67401 Illkrich, France

^7^ Plateforme eBioCyt, Faculté de Pharmacie & Féderation Translationelle de Médecine, Université de Strasbourg, Illkrich, France

Bioprospecting consists on the exploration of biodiversity for new resources of social and comercial value [1]. In this context, the oceans host an unparallel biological and chemical diversity [2]. In particular, microalgae and other phytoplanktonic species like dinoflagellates have contributed a large number of unprecedent structures, some of which could become therapeutically useful agents. Okadaic acid is an example of how compounds from marine organisms may provide opportunities to obtain valuable metabolites. This lipophilic polyether produced by *Dynophysis* and *Prorocentrum genera*, is an inhibitor of protein phospatase 2A (PP2A) and its tumor promoting and cytotoxic activities have been of a great interest in pharmacological research at the molecular level [3]. Furthermore, it is a novel tool for Alzheimer’s disease [4]. Herein, the results of a bioprospecting process comprising 31 strains of marine microalgae (including dinoflagellates, haptophytas, heterokontophytas and one chlorophyta) are showed. The assays were targeted to provide information on antimicrobial, antiproliferative, apoptotic and anti-inflammatory potential of the studied extracts.

##### ***Acknowledgments*** 

To MINECO SAF2011-28883-C03-01, FP7-KBBE-2009-3-245137 (MAREX EU) and FP7-REGPOT-2012-CT2012-316137-IMBRAIM projects. C.R. de Vera to MINECO FPU program. To Santiago Fraga, IEO Vigo, for donating the strain. The research group acknowledge the financing granted to ULL by Agencia Canaria de Investigación, Innovación y Sociedad de la Información, being 85% cofinancied by the European Social Fund.

##### ***References*** 

1. Abida, H.; Ruchaud, S.; Rios, L.; Humeau, A.; Probert, I.; de Vargas, C.; Bach, S.; Bowler, C. Bioprospecting Marine Plankton. *Mar. Drugs*
**2013**, *11*, 4594–4611.
2. Costa, M.; García, M.; Costa-Rodrigues, J.; Costa, M.S.; Ribeiro, M.J.; Fernandes, M.H.; Barros, P.; Barreiro, A.; Vasconcelos, V.; Martins, R. Exploring Bioactive Properties of Marine Cyanobacteria Isolated from the Portuguese Coast: High Potential as a Source of Anticancer Compounds. *Mar. Drugs*
**2014**, *12*, 98–114.
3. Fernández, J.J.; Candenas, M.L.; Souto, M.L.; Trujillo, M.M.; Norte, M. Okadaic acid, a useful tool for studying cellular processes. *Curr. Med. Chem.*
**2002**, *9*, 229–262.
4. Kamat, P.K.; Rai, S.; Swarnkar, S.; Shukla, R.; Nath, C. Molecular and cellular mechanism of okadaic acid (OKA)-induced neurotoxicity: A novel tool for Alzheimer's disease therapeutic application. *Mol. Neurobiol.*
**2014**, *50*, 852–865.

#### **Anti-Diabetic Properties of Seaweed Extract Using the Adipocyte Cell Model 3T3-L1** 

**Margrét Eva Ásgeirsdottir ^1,2^, Eva Kuttner ^1^, Hörður G. Kristinsson ^1,2^ and Guðmundur Óli Hreggviðsson ^1^**

^2^     Matis ohf, Vinlandsleid 12, 113, Reykjavik, Iceland

^2^     Faculty of Food Science and Nutrition, University of Iceland, Eiríksgata 29, 101, Reykjavik, Iceland

Obesity, where the body accumulates an excess amount of fatty tissue is one of the major risk factors for type 2 diabetes. Adipocytes constitute a major part of the fatty tissue and perform key endocrine functions, one of them insulin sensitivity. Regulating adipogenesis through dietary supplementation could be one way of alleviating complications of type 2 diabetes. Polyphenols have been shown to have anti-diabetic properties. Extracts from seaweed, having a high polyphenol content, have revealed antidiabetic and antioxidant properties. The aim of this study was to further investigate the effects of different seaweed extracts on differentiation (adipogenesis), influence on protein expression levels, proliferation and apoptosis of adipocytes. Undifferentiated murine adipocytes (3T3-L1 cell line) were exposed in different concentrations to the seaweed extract and the several key protein isolated and used for expression studies.

#### **Paralytic Shellfish Poisoning Toxins from Mediterranean *Alexandrium minutum* and *A. catenella*: Toxin Profile and *SXT* Gene Content** 

**Luciana Tartaglione ^1^, Carmela Dell’Aversano ^1^, Patrizia Ciminiello ^1^, Federico Perini ^2^, Samuela Cappellacci ^2^, Valentina Sparvoli ^2^, Silvia Casabianca ^2^, Michele Scardi ^3^, Mariagrazia Giacobbe ^4^ and Antonella Penna ^2^**

^1^ Dipartimento di Farmacia, Università degli Studi di Napoli Federico II, Napoli, Italy

^2^ Dipartimento di Scienze Biomolecolari, Università di Urbino, Urbino, Italy

^3^ Dipartimento di Biologia, Università di Tor Vergata, Roma, Italy

^4^ Istituto per l’Ambiente Marino Costiero, CNR, Messina, Italy

Paralytic shellfish poisoning (PSP) toxins are potent water-soluble neurotoxins including the parent compound saxitoxin (STX) and a number of its congeners. They are tetrahydropurine derivatives that can be subdivided into three main groups according to substitution of the side chain: carbamoyl-, *N*-sulfocarbamoyl-, and decarbamoyl-toxins. The carbamoyl derivatives (STX, NEO and GTX1-4) are reported to be the most potent. Due to their accumulation in filter feeding shellfish, PSP toxins can move through the food chain inducing a toxic syndrome in seafood consumers. Symptoms are neurological with rapid onset (30–60 min from ingestion) and include paraesthesia, vertigo, numbness, tingling of the face, tongue, and lip, ataxia, blocking of respiration and even death. Due to the high risk posed to human health by PSP toxins, a multidisciplinary integrated approach based on liquid chromatography high resolution mass spectrometry (LC-HRMS and MS^2^) and qPCR-based assay has been used to depict the PSP toxin scenario in the Mediterranean Sea. As the *sxt*A and the *sxt*G genes are known as the starting genes of PSP toxin synthesis in dinoflagellates, different populations of the Mediterranean *A. minutum* from NW Adriatic, Ionian, Tyrrhenian and Catalan Seas were grown in culture and analyzed by qPCR in order to obtain the quantification of these genes. In parallel, LC-HRMS^2^ analyses were performed on the *A. minutum* cultured strains and revealed for all of them a toxin profile consisting of only GTX1 and GTX4. Toxin production was in the fg/cell range. Concomitantly with a massive natural bloom of *A. minutum* and *A. catenella* that occurred in Spring 2014 along the Syracuse coasts (Sicily, Italy), four seawater samples were collected and analyzed by LC-HRMS and MS^2^. The analyzed extracts were found to contain a variety of PSP toxins, namely STX, NEO, the gonyautoxins GTX1-4, the *N*-sulfocarbamoyl derivatives C1/C2, B1 and B2 and the decarbamoyl STX.

#### **Towards the Search of Novel Bioactive Molecules from North Atlantic Ocean** 

**Nipun Mahajan ^1^, Daria Firsova ^1^, Sylvia Soldatu ^1^, Candice Bromley ^1^, Ryan Young ^1^ and Bill Baker ^1,2^**

^1^     National University of Ireland, Galway, Ireland

^2^     Center for Drug Discovery and Innovation, University of South Florida, FL, USA

The marine microbial community has proven records of providing bioactive molecules useful for the treatment of human diseases. Macro-organisms and sediments from the coastal and deep sea region of North Atlantic Ocean were collected to grow their associated microbes. Using a variety of specialised media enabled the growth of hundreds of bacteria, actinomycetes and fungi. The isolated pure colonies of bacteria were cultured using broth media whilst fungal strains were cultured on sterilised rice with and without stress factors such as genetic modifiers. These cultured bacteria and fungi were subsequently extracted with lipophilic solvent. The resultant extracts were submitted for high throughput screening against ESKAPE pathogen related microbial diseases, cancer and osteoarthritis, to identify potentially biologically useful microbes. Upon identification of the hit microorganism(s), large scale culturing of the microbes of interest will be undertaken to get sufficient biomass for activity guided isolation of hit molecule(s).

#### **Dereplication Metabolomics, and Rational Approaches to Bioprospecting Oceancharcot: A Program Dedicated to the Screening of Marine Chemodiversity** 

**Benoît Serive ^1,2^, Ronald J. Quinn ^1^ and Stéphane Bach ^2^**

^1^ Eskitis Institute for Drug Discovery, Griffith University, Brisbane, Australia

^2^ Kinase Inhibitor Specialized Screening Facility, USR3151 CNRS/UPMC, Station Biologique de Roscoff, Roscoff, France

Oceans dominate the surface of our planet and they are the chest of a huge biodiversity, resulting from an adaptive evolution to many ecological niches. Amongst the 33 known phyla on earth, 21 are exclusively marine. The aim of the OCEANCHArCoT project is to explore the chemodiversity from marine organisms to find new chemical scaffolds able to inhibit cellular targets. Amongst the latter, protein kinases are involved in various human pathologies such as cancer and neurodegenerative diseases, or can be targeted to alter the life cycle of various parasites. Some protein kinases are also implicated in cellular events leading to necroptosis. Necroptosis is of central pathological relevance in neurodegenerative diseases, inflammatory diseases, viral and bacteria infections, allograft rejection, ischemia-reperfusion injury, brain injuries, atherosclerosis, Gaucher’s disease or in age-related macular degeneration. We decided to focus on some key protein kinases of the necrosome to guarantee a large potential of pharmacological applications. The workflow was designed to make efficient the way leading to the identification of new inhibitors by combining several advantages:

(i) Exploitation of a huge potential of chemodiversity: Nature Bank library (Eskitis Institute for Drug Discovery, Brisbane, Australia) and planktonic extracts from cultivatable strains (OCEANOMICs project, Station Biologique de Roscoff—CNRS, Roscoff, France).
(ii) Using advanced technologies such as a high-throughput screening robot (BioCel 900, Agilent Technologies) and bioaffinity mass spectrometry (ESI-FTICR-MS, Bruker).

This program of Excellence is supported by a Marie Curie International Outgoing Fellowship grant from European Commission.

#### **Accurate Dereplication of Bioactive Secondary Metabolites from Marine-Derived Fungi by UHPLC-DAD-QTOFMS and a MS/HRMS Library** 

**Sara Kildgaard, Maria Mansson, Ina Dosen, Andreas Klitgaard, Jens C. Frisvad, Thomas O. Larsen and Kristian F. Nielsen**

Department of Systems Biology, Technical University of Denmark, Kgs. Lyngby, Denmark

Microorganisms from the marine environment are a promising source of new bioactive compounds based on new chemical scaffolds. Although the majority of known compounds originate from bacterial species, marine-derived fungal strains have yielded a plethora of biologically active compounds [1,2]. The most common sources are isolates of *Penicillium* and *Aspergillus*. Due to the cosmopolitan occurrence of many bioactive compounds, most natural product extracts contain compounds that have previously been characterized. In drug discovery, reliable and fast dereplication of known compounds is therefore essential for identification of novel bioactive compounds. This study presents an integrated approach using ultra-high performance liquid chromatography-diode array detection-quadrupole time of flight mass spectrometry (UHPLC-DAD-QTOFMS) with tandem high resolution MS (MS/HRMS) analysis to screen extracts from bioactive marine-derived *Aspergillus*, *Penicillium*, and *Emericellopsis* strains [1]. The chosen strains were selected from a screening conducted as part of the PharmaSea project [3]. Dereplication of known compounds, including small polyketides, non-ribosomal peptides, terpenes and meroterpenoids in the bioactive extracts were done by searching the MS/HRMS data against a newly conducted in-house MS/HRMS library of ~1300 compounds (10, 20, and 40 eV spectra) using the Agilent software [3].

##### ***Acknowledgments*** 

We acknowledge support from EU for the FP7 PharmaSea project and from Agilent for Thought Leader Award of new MS-instrumentation.

##### ***References*** 

1. Kildgaard, S.; Mansson, M.; Dosen, I.; Klitgaard, A.; Frisvad, J.C.; Larsen, T.O.; Nielsen, K.F. Accurate Dereplication of Bioactive Secondary Metabolites from Marine-Derived Fungi by UHPLC-DAD-QTOFMS and a MS/HRMS Library. *Mar. Drugs*
**2014**, *12*, 3681–3705.
2. Debbab, A.; Aly, A.H.; Lin, W.H.; Proksch, P. Bioactive Compounds from Marine Bacteria and Fungi. *Microb. Biotechnol.*
**2010**, *3*, 544–563.

#### **The Role of Marinlit as a Dereplication Tool: Biogeography** 

**Serin Dabb ^1^, Helen Potter ^1^, Jeff White ^1^, John Blunt ^2^ and Murray Munro ^2^**

^1^ Royal Society of Chemistry, Cambridge, UK

^2^ University of Canterbury, Christchurch, New Zealand

To actively pursue bioactive compounds from natural sources data covering five disparate sources are required—biogeography, taxonomy, access to biodata as well as dereplication and characterisation tools. Fortunately for those working in marine natural products one database, MarinLit covers all these areas. There are three aspects of MarinLit that are not found in any other database:

- biogeography;
- functional group recognition; and
- access to biodata.

These data are vital in the selection of collecting sites, dereplication and appropriate assays for compounds of interest. *Biogeography is described in this poster and the functional group recognition aspects are described in an adjacent poster. Biogeography*, the description and understanding of the spatial patterns of biodiversity, is an ecological tool seldom associated with natural products research. But, information on biodiversity hotspots, species distribution and latitudinal/longitudinal gradients can be vital to understand and predict the distribution of marine natural products, lead to a better understanding of spatial and temporal patterns in chemotaxonomic studies of taxa of interest, and ultimately increase the success of natural product discovery. The paucity of biogeographical information for marine species has been addressed by MarinLit by the incorporation of geographical/depth data in a searchable web-based format and is the only natural product database able to provide and search biogeographical data, or combine those data with other typical database features such as substructure searching. Examples will be provided to demonstrate the potential of biogeography as a tool to provide new insights into future bioprospecting efforts.

#### **The Role of Marinlit as a Dereplication Tool: Functional Group Recognition** 

**Serin Dabb ^1^, Helen Potter ^1^, Jeff White ^1^, John Blunt ^2^ and Murray Munro ^2^**

^1^ Royal Society of Chemistry, Cambridge, UK

^2^ University of Canterbury, Christchuch, New Zealand

To actively pursue bioactive compounds from natural sources data covering five disparate sources are required—biogeography, taxonomy, access to biodata as well as dereplication and characterisation tools. Fortunately for those working in marine natural products one database, MarinLit covers all these areas. There are three aspects of MarinLit that are not found in any other database:

- biogeography;
- functional group recognition; and
- access to biodata.

These data are vital in the selection of collecting sites, dereplication and appropriate assays for compounds of interest. *Functional group recognition is described in this poster and the biogeography aspects are described in an adjacent poster. Functional group recognition*. This approach is very simple, but extraordinarily discriminating. ^1^H-NMR spectra are information-rich and allow the ready recognition of a wide variety of functional groups—methyl groups, acetal protons, a-protons in peptides, carbinol, and olefinic protons and aromatic substitution patterns. MarinLit uses a specifically developed algorithm that recognizes a wide range of functional groups within a structure leading to each entry in MarinLit being seeded with the numbers of these functional groups observable in a ^1^H-NMR spectrum in searchable fields. By simply counting the number and types of features (methyl groups, aromatic substitution patterns, *etc.*) in a ^1^H-NMR spectrum and searching MarinLit all compounds that match are recognised. If there is no match the compound is new to marine natural products. Recent advances in the detection algorithm allow for the recognition of a wider range of aromatic and heteroaromatic systems. MarinLit is the only database with functional group recognition and it is possible to combine those data with other typical database features such as substructure searching. Examples will be provided that demonstrate the potential of functional group recognition as a dereplication tool.

#### **An Easy, Reproducible and Semi-Automated Protocol for Bioassay-Guided Fractionation of Marine Extracts** 

**Genoveffa Nuzzo ^1^, Adele Cutignano ^1^, Elvira Luongo ^1^, Carmela Gallo ^1^, Giovanna Romano ^2^, Adrianna Ianora ^2^ and Angelo Fontana ^1^**

^1^ CNR-ICB, Pozzuoli, Napoli, Italy

^2^ SZN, Napoli, Italy

Marine biodiversity is a rich source of a great variety of novel chemical entities some of which are endowed with pharmaceutical properties [1,2]. The discovery of these products relies on different approaches but the most common entails bioassay-guided fractionation of marine extracts. Several academic and industrial groups are familiar with high-throughput screening (HTS) to assay a large number of potential chemical agents against a chosen set of defined biological targets. The success of this method requires the overcoming of several crucial points, as removing of salts that led to overlooking bioactive components and emphasizing the presence of minor compounds often masked in the raw material. Here we present an easy, versatile and efficient fractionation procedure to screen marine organism extracts based on Solid Phase Extraction (SPE). The protocol relies on a polystyrene based adsorbent as solid support and a stepwise organic solvent elution. The method has been validated on three selected marine organisms, *i.e.*, *Dendrilla membranosa*, *Reniera sarai* and *Amphidinium carterae*, whose secondary metabolism has been previously investigated. The results indicated that the main classes of primary and secondary metabolites exhibited a predictable and reproducible distribution within five fractions. By using an automated chromatographic platform, the method has been miniaturized thus allowing to quickly handle several samples at laboratory scale with significant saving of time and solvent. Combined with routinely NMR and MS analyses, the platform produces a rich data set over a relatively short space of time and supports the development of a home-made library of natural products. In support of this, we briefly report the application of our protocol to two discovery programs for immuno-modulatory and anti-infective agents from sponges and cultivable marine organisms.

##### ***References*** 

1. Blunt, J.W.; Copp. B.R.; Keyzers, R.A.; Munro, M.H.; Prinsep, M.R. Marine natural products. *Nat. Prod. Rep.*
**2015**, *32*, 116–211.
2. Newman D.J.; Giddings L.A. Natural products as leads to antitumor drugs. *Phytochem. Rev.*
**2014**, *13*, 123–137.

#### **A Time-Scale Metabolomics Study Reveals Biosynthetic Pathways in Marine-Sourced *Penicillium* Cultures** 

**Catherine Roullier, Mathilde Peigné, Yves François Pouchus and Olivier Grovel**

Faculté de Pharmacie, Université de Nantes, MMS, Nantes, France

The field of research combining natural products chemistry and metabolomics studies is currently growing. When trying to unravel cryptic biosynthetic pathways from microorganisms, different methods are usually performed such as targeted mutagenesis, changes in culture media, use of different types of elicitors, as well as co-cultivation of organisms. Most research in this field statistically compares the metabolic profiles (either LC-MS or NMR or both) generated after a selected time of cultivation under the different treatments applied to the microorganisms in order to identify interesting compounds. Even though it is well assumed that metabolism is a function of the development stage of an organism and consequently changes over time, so far, few studies have compared metabolic profiles through time. Then, it can be assumed that such studies can reveal new metabolic intermediates in biosynthetic pathways. In the work presented here, a time-scale metabolomic study has been performed on different marine-derived* Penicillium* strains, for which crude culture extracts were analyzed by HPLC-DAD-HRMS each day for an 18-day period. Differences in terms of final metabolites, intermediates and kinetics of production have been highlighted for strains belonging to different *Penicillium *species. The results of this study will be given with a particular focus on a common biosynthetic pathway in all strains leading to griseofulvin, a drug which is still on the market and regains interest in the field of cancer.

#### **^13^C NMR as a Metabolomic Tool for Identification of Individual Components of Algal Extracts: An Application to the Chemiodiversity of *Laurencia microcladia* from Corsica (Ajaccio Bay)** 

**Sylvain Sutour, Ange Bighelli, Vincent Castola and Mathieu Paoli**

University of Corsica, Equipe Chimie et Biomasse, Ajaccio, France

The genus *Laurencia* is one of the most prolific algal sources of highly diverse secondary metabolites exhibiting tremendous biological activity. In order to realize a metabolomic study of extracts from *Laurencia microcladia *growing wild on the Corsican Coast, we applied the analytical method based on ^13^C-NMR developed in our laboratory. The recorded ^13^C NMR spectra of pentane and ethyl acetate extracts followed by a computer-aided analysis, allowed the identification 9 compounds belonging to terpene, acetogenin and sterol families. 3(*E*)-laurenyne is the main compound, followed by 3(*Z*)-laurenyne, cholesterol, obtusallene IA and a dibromochamigrene derivative. Among the non polar compounds (pentane extract) we can identify four known compounds bearing a laurane (laurene and dihydrolaurene) and a cuparane (A-bromocuparene and A-isobromocuparene) skeleton. All the previously identified compounds are described for the first time in extracts from *Laurencia microcladia*. Moreover, no compounds previously described from *Laurencia microcladia *and *Laurencia obtusa* from Greece and Italy were identified from the Corsican extracts. As suggested by F. Pietra, there is a close relationship between a species and a chemical family more than individual compound. Thus, *Laurencia microcladia* from Corsica is different from *Laurencia microcladia* from Greece which produces mainly sesquiterpenes bearing cuparane and laurane skeleton. Its metabolomic profile is closer to those described in Italy by the occurrence of C_15_ acetogenin represented by 3*E*-laurenyne as major component. However, a difference is still remaining between C_15_ acetogenins from extracts from Italy and from Corsica. Indeed, oxepanes characterize extracts from Italy whereas seven members ring acetylenic ethers characterize extracts from Corsica. ^13^C NMR as proved to be an efficient metabolomic tool for identification of individual components of algal extracts. The unambiguously identification of stereoisomers exhibiting close or net enough differentiated mass spectra like: 3*E vs.* 3*Z*-laurenyne and A-bromocuparene *vs.* A-isobromocuparene appear to be a strong advantage of this method.

#### **Biodiversity, Anti-Trypanosomal Activity Screening, and Metabolomics Profiling of Actinomycetes Isolated from Mediterranean Sponges** 

**Cheng Cheng ^1^, Lynsey MacIntyre ^2^, Usama Ramadan Abdelmohsen ^1,4^, Hannes Horn ^1^, Paraskevi N. Polymenakou ^3^, RuAngelie Edrada-Ebel ^2^ and Ute Hentschel ^1^**

^1^ Department of Botany II, Julius-von-Sachs Institute for Biological Sciences, University of Würzburg, Würzburg, Germany

^2^ Strathclyde Institute of Pharmacy and Biomedical Sciences, University of Strathclyde, Glasgow, UK

^3^ Hellenic Centre for Marine Research, Institute of Marine Biology, Biotechnology and Aquaculture, Crete, Greece

^4^ Department of Pharmacognosy, Faculty of Pharmacy, Minia, Egypt

Marine sponge–associated actinomycetes are considered as promising sources for the discovery of novel biologically active compounds. In the present study, a total of 64 actinomycetes were isolated from 12 different marine sponge species that had been collected from offshore the islands of Milos and Crete, Greece, eastern Mediterranean. The isolates were affiliated to 23 genera representing 8 different suborders based on nearly full length 16S rRNA gene sequencing. Four putatively novel species belonging to genera *Geodermatophilus*, *Microlunatus*, *Rhodococcus* and *Actinomycetospora* were identified based on a sequence similarity <98.5% to validly described 16S rRNA gene sequences. Eight actinomycete isolates showed bioactivities against *Trypanosma brucei brucei* TC221 with half maximal inhibitory concentration (IC_50_) values <20 µg/mL. Thirty four isolates from the Milos collection and 12 isolates from the Crete collection were subjected to metabolomics analysis using high resolution LC-MS and NMR for dereplication purposes. Two isolates belong to the genera *Streptomyces *(SBT348) and *Micromonospora* (SBT687) were prioritized based on their distinct chemistry profiles as well as their anti-trypanosomal activities. These findings demonstrated the feasibility and efficacy of utilizing metabolomics tools to prioritize chemically unique strains from microorganism collections and further highlight sponges as rich source for novel and bioactive actinomycetes.

#### **Affinity of Spongionella Isolated Compounds by Cyclophilins** 

**Amparo Alfonso ^1^, Jon A Sánchez ^1^, Eva Alonso ^1^, Mostafa E. Rateb ^2,3^, Wael E. Houssen ^2,4^, Rainer Ebel ^2^, Marcel Jaspars ^2^ and Luis M Botana ^1^**

^1^ Universidad de Santiago de Compostela, Lugo, Spain

^2^ Marine Biodiscovery Centre, Department of Chemistry, University of Aberdeen, Aberdeen, Scotland, UK

^3^ Beni-Suef University, Beni-Suef, Egypt

^4^ Institute of Medical Sciences, University of Aberdeen, Aberdeen, Scotland, UK

Gracilins H, A, L and Tetrahydroaplysulphurin-1 are bioactive diterpene compounds isolated from the marine sponge *Spongionella* able to protect mitochondria from oxidative stress. Cyclophilins (Cyp) are a group of proteins belong to the immunophilins family with different localization, Cyp D in the mitochondrial matrix and Cyp A in the cytosol. The activation of these molecules has important implications in diseases such us alzheimer, cancer and inflammatory alterations. Cyclosporine A (CsA) is a cyclic peptide useful as immunosuppressant agent with high affinity by different immunophilins. Since the effects of Spongionella diterpenes over mitochondria and calcium fluxes were similar to the effects of CsA, the aim of this work was to study the association between all these compounds and cyclophilins by using a biosensor. This instrument measures biomolecular interactions in real time and allows the calculation of kinetic constants. Cyp A and D were immobilized onto the sensor surface at pH 4.5 or 6. Over these immobilized ligands the association with CsA and Spongionella compounds was studied. When CsA was used typical association curves were obtained at any condition studied, showing higher affinity when the immobilization was done at pH 6. In the presence of Spongionella compounds different results were obtained. When cyclophilins were immobilized at pH 6 no interactions with Spongionella derivatives were observed. With Cyp D immobilized at pH 4.5, typical association curves were obtained when Gracilin L or Tetrahydroaplysulphurin-1 were added, while no interactions were observed with Gracilin A and H. When Cyp A was immobilized at pH 4.5, good association profiles were obtained in the presence of Gracilin L and H, although lower than CsA. However Gracilin A and Tetrahydroaplysulphurin-1 showed higher affinity by Cyp A than CsA. These results suggest that Spongionella diterpenes are novel cyclophilins inhibitors with affinity comparable to that of immunosuppressive CsA.

#### **From Chemical Profile to Biological Profile, Brominated Metabolites from the Bryozoan *Amathia tortuosa* Produce Phenotypic Effects on Parkinson’s Disease Cells** 

**Yousef Dashti, Marie-Laure Vial, Stephen A. Wood, George D. Mellick, Catherine Roullier and Ronald J Quinn**

Eskitis Institute for Drug Discovery, Griffith University, Brisbane, QLD, Australia

NMR metabolite fingerprints of fractions from an extract of the bryozoan *Amathia tortuosa* allowed identification of three known and evidence of a new tribrominated metabolite. The new compound, kororamide B, was purified through NMR-guided isolation and the structure assigned by mass and detailed NMR analysis. The absolute configuration of the new compound was established by comparison of the experimental and calculated electronic circular dichroism spectra. The compounds displayed effects on early endosomes when profiled on human olfactory neurosphere-derived cells (hONS) derived from a Parkinson’s disease patient using a multidimensional phenotypic assay.

#### **Scottish Seaweed Species as a Source of Bioactive Natural Products** 

**Kenneth Boyd and Robbie Mutton**

University of the HIghlands and Islands, Thurso, UK

In the 1970’s marine natural products research was largely focused on cataloguing novel metabolites from red and brown algae. However interest in macroalgae was quickly overtaken by studies on other phyla, especially sponges, tunicates and latterly microorganisms. The movement away from studying macroalgae coincided with the development of screening programmes aimed at identifying compounds with promising pharmacological properties. As a result the pharmacological potential of macroalgal metabolites has been largely unexplored, leading to them being described as one of the forgotten phyla in marine natural products chemistry. Macroalgae are ubiquitous in the majority of near shore habitats and therefore represent an easily accessible and potentially valuable resource to the natural products chemist. Here we examine marine algae from Caithness (Scotland) as a source of bioactive natural metabolites. A survey of 18 sites revealed a wide diversity of seaweed species in the area, with 22 green, 43 brown and 69 red algal species identified. From these, metabolites have previously been reported from only 20 (15%) with no reports of natural products from the remaining 114 (85%). Polar and non-polar extracts of 13 species of algae were prepared and subjected to a battery of assays including antioxidant, antimicrobial, anti-inflammatory and cyctotoxicity. From the results 57.6% of the polar extracts showed activity in at least one of the bioassays with 84.6% of the non-polar extracts showing activity. A series of meroterpenes from H. siliquosa exhibited potent antioxidant activity. This demonstrates the potential of regional collection of macroalgae as a source of bioactive metabolites.

### **Biosynthesis of Marine Natural Products in Microbes** 

#### **Rational Design of Patg_mac_, the Macrocyclase from Patellamide Pathway, to Accelerate the Production of Cyclic Peptides** 

**Fabio Tamaki ^1,2^, Wael Houssen ^1,2^ and Marcel Jaspars ^1^**

^1^ Marine Biodiscovery Centre, Department of Chemistry, University of Aberdeen, Meston Walk, Aberdeen AB24 3UE, UK

^2^ Institute of Medical Sciences, University of Aberdeen, Aberdeen AB25 2ZD, UK

The biosynthesis of the ribosomally-synthesised and post-translationally modified patellamides involves enzymatic processing of a precursor linear peptide that contains a leader sequence for recognition by the heterocyclase PatD, a core sequence to be processed and cleavage signals for the protease PatA and the macrocyclase PatG_mac_. PatG_mac_ is a very slow enzyme with a turnover rate of one molecule/day. It can process core sequences containing 6–11 amino acids of which the last residue is either a proline or a heterocyclised amino acid. PatG_mac_ helix-loop-helix insertion plays a crucial role for macrocyclisation since its deletion resulted in enzymes presenting only proteolytic acticity but no macrocyclisation activity [1]. We used a combination of bioinformatics and structural data to identify positions that can be varied to improve PatG_mac_ kinetics and to eliminate the requirement for proline or heterocycle in the core sequence. Co-variation analyses using PatG_mac_ Protein Family Statistic [2] yielded, amongst others, five co-variant positions near the active site residues as targets for introducing variability aiming speed up macrocyclase enzymes. Moreover, three additional positions near the subsite that houses the substrate heterocyclised residue (PDB:4AKT) [1], will be further targeted for variability introduction aiming new enzymes with no need for heterocyclised amino acids.

##### ***References*** 

1. Koehnke, J.; Bent, A.; Houssen, W.E.; Zollman, D.; Morawitz, F.; Shirran, S.; Vendome, J.; Nneoyiegbe, A.F.; Trembleau, L.; Botting, C.H.; *et al*. The mechanism of patellamide macrocyclization revealed by the characterization of the PatG macrocyclase domain. *Nat. Struct. Mol. Biol.*
**2012**, *19*, 767–772, doi:10.1038/nsmb.2340.
2. Bleicher, L.; Lemke, N.; Garratt, R.C. Using Amino Acid Correlation and Community Detection Algorithms to Identify Functional Determinants in Protein Families. *PLoS ONE*
**2011**, *6*, e27786, doi:10.1371/journal.pone.0027786.

#### **The *in Vitro* Use of Cyanobactin Prenylases in the Preparation of Bioactive Modified Cyclic Peptides** 

**Luca Dalponte ^1,2^, Wael Houssen ^1,2^, David Fewer ^3^, Christian Umeobika ^1^, Laurent Trembleau ^1^ and Marcel Jaspars ^1^**

^1^ Marine Biodiscovery Centre, Department of Chemistry, University of Aberdeen, Aberdeen, UK

^2^ Institute of Medical Sciences, University of Aberdeen, Aberdeen, UK

^3^ Microbiology and Biotechnology Division, Department of Food and Environmental Sciences, University of Helsinki, Helsinki, Finland

In the last decade, the interest in macrocycles and constrained cyclic peptides has grown exponentially due to their high resistance to enzymatic digestion and high receptor binding activity when compared to their linear counterparts. However, a main challenge to develop these compounds as therapeutics is the limited ability for many compounds to cross membrane barriers. Prenylation, a post-translational modification that involves addition of a prenyl group to amino acid residues, could increase the lipophilicity of the cyclic product and thus its ability to cross cellular membranes. Cyanobactins include many examples of prenylated cyclic peptides such as trunkamides from *Prochloron* spp. and aestuaramides from *Lyngbya aestuarii*. Our group has identified the structure of PatF prenylase from Prochloron. However PatF is a non-functional prenylase which is in accordance with the fact that none of the patellamides is prenylated. More recently, Schmidt group studied LynF prenylase that is capable of processing oxygen containing amino acids such as Ser, Thr and Tyr. In tyrosine, the process is followed by spontaneous nonenzymatic Claisen rearrangement generating the C-prenylated derivative. The aim of this project is to study homologues of LynF, in order to identify enzymes that can process other amino acids, e.g., tryptophan and identifying the key structural features responsible for substrate specificity.

#### **Tamd: An α-Oxoamine Synthase Enzyme Involved in the Biosynthesis of the Marine Natural Product Tambjamine YP1** 

**Piera Marchetti ^1^, Daynea Wallock-Richards ^1^, Van Kelly ^1^, Stephen McMahon ^2^, James H. Naismith ^2^ and Dominic Campopiano ^1^**

^1^ University of Edinburgh, Edinburgh, UK

^2^ University of St. Andrews, St. Andrews, Fife, UK

Tambjamine YP1 is a natural product produced by the marine microorganism *Pseudoalteromonas tunicata*, which colonises the surface of the sea lettuce *Ulva australis* and prevents biofouling. The tambjamines are a family of bipyrrolic natural products with cytotoxic and antimicrobial activity that also act as anion transporters. The gene cluster encoded in the *P. tunicata* genome for tambjamine YP1 production was identified by Kjelleberg *et al*. and contains 19 genes in one operon. The TamD gene is predicted to encode an unusual two domain enzyme that is composed of an *N*-terminal acyl-carrier protein (ACP) fused to a *C*-terminal SerT domain which is a member of the α-oxoamine synthase (AOS) family. We hypothesise that TamD condenses l-serine with an ACP-bound substrate using a pyridoxal 5′-phosphate (PLP) dependent Claisen-like decarboxylation reaction. The TamD product would subsequently cyclise to form the bipyrrole moiety of tambjamine YP1. Not only are these enzymes interesting for the production of tambjamines for clinical studies but pyrrole structures are also important motifs in many other drugs [3]. The full length TamD enzyme has been heterologously expressed in *Escherichia coli* as well as the separate ACP and SerT domains. Loading of the ACP and binding of the substrates to the AOS domain are being investigated by mass spectrometry and UV/Visible spectroscopy. Efforts to obtain a crystal structure of the enzyme are underway and will hopefully shed light on the substrate binding and mechanism of action of the enzyme. In the future the enzyme kinetics will be probed using synthetic substrates and other recombinant enzymes from the tambjamine YP1 biosynthetic pathway.

#### **Phenalenones: Insight into the Biosynthesis of Polyketides from the Marine Alga-Derived Fungus *Coniothyrium cereale*** 

**Mamona Nazir ^1^, Fayrouz El Maddah ^1^, Stefan Kehraus ^1^, Ekaterina Egereva ^1^, Jörn Piel ^2^, Alexander O. Brachmann ^2^ and Gabriele M. König ^1^**

^1^ University of Bonn, Institute for Pharmaceutical Biology Nussallee 6, 53115 Bonn, Germany

^2^ Institute for Microbiology, ETH Zürich, Wolfgang Pauli Strasse 10, 8093 Zürich, Switzerland

The marine-derived fungus Coniothyrium cereale was isolated from the Baltic sea alga Enteromorpha sp., is a profile producer of phenalenones. Upon cultivation on solid marine medium, it produced an impressive range of polyketides with unique structures and remarkable biological activities [1–3]. To establish the biosynthetic precursors of the polyketides (−)-cereolactam and (−)-cereoaldomine, which inhibited Human Leukocyte Elastase (HLE) with an IC50 value of 9.28 and 3.01 µM respectively; *C. cereale* was cultivated with 13C-labeled precursors. We successfully elucidated the biosynthetic origins of the carbon skeleton of (−)-cereolactam and trypethelone by intensive 13C NMR techniques. Fractions containing the (−)-cereoaldomine were analysed by UPLC-MS. MS spectra showed signals indicating the presence of seven acetate units, five for the polyketide part and two for the terpene unit (M + 1 to M + 7). According to our putative biosynthetic pathway, respective metabolites originate from heptaketide precursors of which some carbons are lost by oxidative cleavage. In the same way we established the biosynthetic building blocks of the antimicrobial polyketides coniosclerodin and cereolactone.

##### ***References*** 

1. Elsebai, M.F.; Nazir, M.; Kehraus, S.; Egereva, E.; Ioset, K.N.; Marcourt, L.; Jeannerat, D.; Gütschow, M.; Wolfender, J.L.; König, G.M. Polyketide Skeletons from the Marine Alga-Derived Fungus *Coniothyrium cereale*. *Eur. J. Org. Chem.*
**2012**, *2012*, 6197–6203.
2. Elsebai, M.F.; Kehraus, S.; Lindequist, U.; Sasse, F.; Shaaban, S.; Gütschow, M.; Josten, M.; Sahl, H.G.; König, G.M. Antimicrobial phenalenone derivatives from the marine-derived fungus *Coniothyrium cereale*. *Org. Biomol. Chem.*
**2011**, *9*, 802–808.
3. Elsebai, M.F.; Kehraus, S.; Mohamed, I.E.; Schnakenburg, G.; Sasse, F.; Shaaban, S.; Gütschow, M.; König, G.M. HLE-Inhibitory Alkaloids with a Polyketide Skeleton from the Marine-Derived Fungus *Coniothyrium cereal*. *J. Nat. Prod.*
**2011**, *74*, 2282–2285.

#### **New Amphidinols from the Dinoflagellate *Amphidinium carterae* Collected at Fusaro Lake (Naples): Structural Characterization and Biosynthesis** 

**Adele Cutignano, Genoveffa Nuzzo, Angela Sardo and Angelo Fontana**

CNR-ICB, Pozzuoli, Napoli, Italy

Amphidinols are a family of antifungal and hemolytic polyhydroxy-polyene polyketides typical of dinoflagellates of the genus *Amphidinium *[1]. The first member of the series was isolated from *Amphidinium klebsii *in 1991 [2] and a few congeners have been reported so far [3,4]. In the frame of our ongoing research on bioactive natural products from marine protists, we started assembling a collection of marine dinoflagellates from the gulf of Naples. An epiphytic strain was isolated from the brown macroalga *Dictyota dichotoma* collected in the brackish waters of Fusaro Lake (Naples) and taxonomically identified as *Amphidinium carterae*. From the methanolic extract of the microalgal pellet we characterized two new amphidinol analogs, named amphidinol-20 (AM20) and its 7-sulfate derivative (amphidinol-21, AM21). Along with the identification of these molecules and the evaluation of the antifungal activity of AM20, here we report the elucidation of their biosynthesis by NMR studies on the 13C-labelled derivatives after experiments with 1-13C acetate, 2-13C acetate, 1,2-13C acetate and 1-13C glycolate.

##### ***References*** 

1. Kobayashi, J.; Kubota, T. *Comprehensive Natural Products II Chemistry and Biology*; Mander, L., Lui, H.-W., Eds.; Elsevier: Oxford, UK, 2010; Volume 2, pp. 263–325.
2. Satake M.; Murata, M.; Yasumoto, T.; Fujita, T.; Naoki, H.J. Amphidinol, a polyhydroxypolyene antifungal agent with an unprecedented structure, from a marine dinoflagellate, *Amphidinium klebsii*. *J*. *Am. Chem. Soc.*
**1991**, *113*, 9859–9861.
3. Espiritu, R.A.; Matsumori, N.; Tsuda, M.; Murata, M. Direct and Stereospecific Interaction of Amphidinol 3 with Sterol in Lipid Bilayers. *Biochemistry*
**2014**, *53*, 3287–3293.
4. Nuzzo, G.; Cutignano, A.; Sardo, A.; Fontana, A. Antifungal amphidinol 18 and its 7-sulfate derivative from the marine dinoflagellate *Amphidinium carterae*. *J. Nat. Prod.*
**2014**, *77*, 1524–1527.

#### **Citrinin and Other Polyketides from *Penicillium citrinum*, an Endophyte from the Marine Red *Alga Dichotomaria* marginata** 

**Teresinha Andrade ^1,2^, Luciano Puzer ^3^, Angela Araujo ^2^, Marcel Jaspars ^4^ and Dulce Silva ^2^**

^1^ IFPI, Teresina, Brazil

^2^ Inst Chem. UNESP, Araraquara, Brazil

^3^ UFABC, Sao Paulo, Brazil

^4^ University of Aberdeen, Aberdeen, UK

Marine organisms may host a variety of endophytic fungi, which induce or enhance production of secondary metabolites associated to important features in adaptation, defense against predators, and represent an important source for bioprospection. *Dichotomaria marginata* is a marine red alga collected in the Brazilian southeast coast which afforded a fungal strain identified as *Penicillium citrinum*. Its crude extract afforded citrinin and three derivatives: dicitrinin A, decarboxydihydrocitrinone, and esclerotinin A, along with one xanthone, one anthraquinone and other polyketides, by chromatographic fractionation and purification by HPLC. The secondary metabolites identification was carried out mainly by ESIMS, NMR analyses and search in Antimarin2011^®^, MarinLit^®^ and ChemSpider^®^ databanks. In addition, their inhibitory activities towards kallikreins were evaluated. Kallikreins are serine proteases comprising a group of 15 peptidases, which may be expressed in several organs, are involved in cell signaling and constitute important tumor biomarkers. KLK3 and KLK2 are prostate cancer markers, whereas KLK1 is expressed in kidneys, intestine and salivary glands, KLK7 is expressed in esophagus, kidneys and liver; and KLK5 is expressed in breast, CNS, prostate and trachea. Best results were observed for citrinin, which inhibited KLK1, KLK3, KLK5 and KLK7 with IC50 values of 1.27 mM, 9.57 mM, 6.60 mM and 2.51 mM, respectively. Citrinin inhibitory activity was also evaluated towards trypsin and papain with IC50 values of 0.16 mM and 0.38 mM, respectively. Such results corroborate the huge chemodiversity of endophytic marine fungi secondary metabolites and may represent an important contribution for the sustainable exploration of marine biodiversity in the search for bioactive compounds.

#### **Antifouling Thielavins from the Marine-Derived Fungus *Thielavia* sp.** 

**Zhuang Han ^1,3^, Ying Xu ^2,3^ and Pei-Yuan Qian ^3^**

^1^ Sanya Institute of Deep-sea Science and Engneering, Chinese Academy of Sciences, Sanya, China

^2^ Division of Life Science, College of Life Science, Shenzhen University, Shenzhen, China

^3 ^The Hong Kong University of Science and Technology, Hong Kong, China

Marine natural products are regarded as a potential source of non-toxic antifoulants that can effectively prevent larval settlement of fouling organisms on man-made marine surfaces. In this study, we used bioassay-guided isolation procedures to purify and characterize 14 new depsides, thielavin U–Z (**1**–**6**) and thielavin Z1–Z8 (**8**–**14**), together with four known compounds, thielavin A, H, J, and K (**15**–**18**), from the EtOAc extract of the marine-derived fungal strain *Thielavia* sp. UST030930-004. All of the compounds were evaluated with respect to their antifouling potential against cyprids of the barnacle *Balanus amphitrite*, and the results showed that compounds **4**–**6** and **9**–**16** were active (EC_50_ values ranging from 2.95 ± 0.59 to 69.19 ± 9.51 μM, respectively). The inhibitive effects of some active compounds were reversible. This is the first report to describe antifouling activities of thielavins against barnacle cyprids.

#### **Bioactive Compounds from Five Marine-Derived Fungal Strains of the South China Sea** 

**Shu-Hua Qi, Xiao-Yong Zhang, Xin-Ya Xu, Xu-Hua Nong and Jie Wang**

South China Sea Institute of Oceanology, Chinese Academy of Sciences, Guangzhou, China

It is now well known that diverse fungal community is abundant in marine environments, and marine fungi have received considerable attentions as important sources for new drug discovery. In order to obtain new bioactive compounds from marine microorganisms, we isolated culturable fungi from different deep-sea sediment cores and coral samples from the South China Sea. Combined with chemical analysis by HPLC and LC-MS, and various bioassay tests, such as antibacterial, antifungal, antifouling and cytotoxic activity, some bioactive fungal strains were chosen for further study of their bioactive secondary metabolites. Recently, we have obtained more than 30 new compounds with obvious antifouling, cytotoxic, antioxidant or enzyme inhibiting activity from five marine-derived fungal strains including *Penicillium* sp., *Aspergillus* sp., *Xylariaceae* sp., *Engyodontium *sp., *Trichobotrys* sp. For several antifouling compounds, their antifouling activities were further evaluated in field test. Two new chromones showed significant cytotoxicity against several carcinoma cell lines, their structure-activity relationships were discussed, and their mechanisms of antitumor were probed. Three compounds with strong inhibiting activity towards acetylcholinesterase with IC50 < 0.1 μM. And several compounds showed stronger antioxidant activity than Vitamin C.

#### **Searching of Non Ribosomal Peptides Anti *Vibrio* from Collection of Indonesan Marine Actinobateria** 

**Noer Kasanah, Triyanto Triyanto, Drajad Seto, Bagash Kurniadi, Muhammad Arrahman and Wisnu Adisusila**

Universitas Gadjah Mada, Yogyakarta, Indonesia

*Vibrio *spp. are a Gram-negative bacteria that dominate the aquatic habitat. Infection by *Vibrio *spp. can occur in humans through contaminated seafood, open wound and can cause cholera and vibriosis. *V. parahaemolyticus *causes a diarrhea and* V. vulnificus *infection can be fatal in immunocompromised patients. Infection by *Vibrio* spp. also one of major problem in fish and shrimp aquaculture. The choice of antibiotic for therapy in humans are limited as well as antibiotic use in aquaculture is restricted. Therefore we need a new anti-infective compounds to control of *Vibrio* spp. infections in human and aquaculture. The objective of this research was to discover new bioactive compounds as Antivibrio based on selection of potential marine Actinobacteria. Collection of marine Actinobacteria collected from Tulamben, Indonesia were subjected for screening based on the presence of nrps gene and bioactivity against *Vibrio* sp. Detection of nrps gene was done by PCR and bioactivity was examined by 96 well format and bioautography. We were able to select potential marine Actinobacteria with desired activity and target gene for further structure determination.

**Keywords:** Indonesia; actinobacteria; non ribosomal peptide; *Vibrio*

#### **Anti-Cancer and Apoptosis-Inducing Activities of *Streptomyces* A16-1 an Isolate from Coastal Soil in the East Gulf of Thailand** 

**Rattanaporn Srivibool ^1^ and Chantarawan Saengkhae ^2^**

^1^ Institute of Marine Science, Burapha University, Chonburi, Thailand

^2^ Faculty of Allied Health Sciences, Burapha University, Chonburi, Thailand

Marine microorganisms have been investigated for decades for novel natural anti-cancer products that are hard to formulate by traditional synthesis. *Streptomyces *are a prolific source of secondary metabolites used in clinical cancer therapy such as daunomycin, doxorubicin, bleomycin. This study *Streptomyces *A16-1 was isolated from coastal soil in Chonburi, Thailand. Cytotoxic activity and apoptotic mechanisms were investigated with partial purification of crude red pigment of the strain. The fractionates were applied to human nasopharynx cell lines (KB cells) and peripheral blood mononuclear cells (PBMCs). Percent cell viability was assessed by MTT assay. The apoptotic effects were evaluated by DAPI nuclear staining, agarose gel electrophoresis, mitochondria staining and Caspase-3 activity. Of 21 fractions, fr7-9, fr10-12, and fr13-16 displayed strong inhibitory effects against KB cells in a dose-dependent manner but less effective against PBMCs. Fraction fr7-9 showed the greatest effect with IC_50_ values of 0.04 ± 0.005, whereas fr10-12, fr13-16 and doxorubicin were, 0.20 ± 0.02, 0.55 ± 0.05 and 1.35 ± 0.23 μg/mL, respectively. Morphological observations showed cell shrinkage, irregular in shape with cytoplasmic granules. Molecular mechanisms of cell death were associated with mitochondrial transmembrane depolarization, chromatin condensation and DNA fragmentation as well as sub G1 fraction of cell cycles. Furthermore, the induction of apoptosis in KB cells was mediated by activated caspase-3 which was significantly diminished in a caspase-3 inhibitor. By 16S rRNA gene sequence analysis and BLAST matching from GenBank database revealed *Streptomyces *A16-1 was 99.101% similarity to *Streptomyces indiaensis*. On chromatograms there were at least eight components in fr7-9, the strong cytotoxic activity and cell apoptosis might cause by a synergistic effect of some or all components while little cytotoxic effect on PBMCs was found which was a good sign. *Streptomyces *A16-1 might have many promising active compounds for cancer chemotherapy. Further efforts to identify the structure and to explore the therapeutic strategy are necessary.

#### **Biological and Chemical Analysis of Bacteria Associated with Common Marine Sponges from Singapore** 

**Deepak Kumar Gupta, Rownak Nazia and Lik Tong Tan**

National Institute of Education, Singapore, Singapore

Marine invertebrates, such as sponges and corals, are hosts to a myriad of microbes and they represent potential sources of bioactive natural products. In this study, we present biological and chemical data of 24 marine microbial strains isolated from three common marine sponges, *Gelliodes fibulata*, *Clathria reinwardti*, and *Xestospongia testudinaria*. Using culture-dependent method, these marine microbes were isolated and cultured using different marine media, namely M1, M2, and M3. The organic extracts of the isolated marine microbes were screened for cytotoxicity property (MOLT-4) and antibacterial activity based on *Escherichia coli* and *Bacillus cereus*. The organic extracts from 12 marine microbial strains were found to display significant anticancer (MOLT cell line) property as well as antibacterial activity against Methicillin-Resistant *Staphylococcus aureus *(MRSA) BAA40, *Escherichia coli* EC598, *Escherichia faecalis* V583,* Pseudomonas aeruginosa*, *Burkholderia cenocepacia* WT-Hill, and *Acinetobacter baumannii*. The phylogenetic data based on 16S rRNA genes of selective marine bacterial strains that showed significant biological activities is also presented. In addition, preliminary chemical data based on LCMS and 1D-NMR spectroscopy of bioactive bacterial-derived extracts/fractions indicated the presence of unique secondary metabolites. This study revealed the biotechnological applications of local marine invertebrate-associated microbes in drug discovery and development efforts.

#### ***Sphaerococcus coronopifolius* Associated Bacteria: A New Source of Antimicrobial and Antitumor Compounds** 

**André Horta, Nádia Fino, Celso Alves, Susete Pinteus, Joana Silva, João Francisco, Américo Rodrigues and Rui Pedrosa**

MARE—Marine and Environmental Sciences Centre, Polytechnic Institute of Leiria, Peniche, Leiria, Portugal

The increase of resistant tumor cells and emergence of antibiotic-resistant bacteria and the need for novel antimicrobial and antitumor compounds stress the search for new bioactive substances. The aim of this study was the isolation and identification of associated bacteria from *Sphaerococcus coronopifolius *and the evaluation of the antitumor and antimicrobial activities of the extracts obtained from the isolated strains. The identification of associated bacteria was determined by 16S rRNA gene sequencing. Bacteria extracts were obtained with methanol and dichloromethane (1:1) extraction. The antimicrobial activity of bacteria extracts was evaluated against seven microorganisms: *Escherichia coli* (ATCC 25922), *Pseudomonas aeruginosa* (ATCC 27853), *Bacillus subtilis* (ATCC 6633), *Salmonella enteritidis *(ATCC 13076), *Staphylococcus aureus *(ATCC 25923), *Saccharomyces cerevisiae* (ATCC 9763) and *Candida albicans* (ATCC 10231). The cell viability and the cell proliferation studies were performed on human breast adenocarcinoma cell line (MCF-7 cells) and a human hepatocellular cancer (HepG-2) according to MTT method. A total of 28 *Sphaerococcus coronopifolius *associated bacteria were isolated and 9 were identified as* Vibrio* sp. (32.14%), *Pseudoalteromonas* sp. (17.86%),* Halomonas* sp. (10.71%), *Shewanella *sp. (17.86%) and *Bacillus* sp. (7.14%). Four (14.29%) of the 28 *Sphaerococcus coronopifolius *associated bacteria presented less than a 90% Basic Local Alignment Search Tool (BLAST) match, and could be new. In antimicrobial assays, four different strains (SP2, SP4, SP7 and SP36) inhibited the *Bacillus subtilis* and Staphylococcus aureus growth. The highest antimicrobial activity against *Bacillus subtilis* was exhibited by SP7 with an IC_50_ 12.99 µg·mL^−1^. The SP36 bacteria extract had a highest antibacterial activity against* Staphylococcus aureus *with an IC_50_ 126.00 µg·mL^−1^. In antitumor tests, SP16 bacteria extract inhibited more than 50% on MCF-7 cell proliferation. In conclusion, the *Sphaerococcus coronopifolius *associated bacteria can be used as source of new marine natural compounds with high antibacterial and antitumoral activity.

#### **Characterization and Photoprotector Activity of Fungal Pigment Isolated from Indonesian Coastal Plant Sarang Semut (*Hydnophytum formicarum*)** 

**Kustiariyah Tarman ^1,2^, Mada Triandala Sibero1 ^1^ and Novriyandi Hanif ^3^**

^1^ Department of Aquatic Products Technology, Faculty of Fisheries and Marine Sciences, Bogor Agricultural University, Jl. Agathis 1, Kampus IPB Darmaga, Bogor, Indonesia

^2^ Center for Coastal and Marine Resources Studies, Bogor Agricultural University, Jl. Raya Pajajaran 1, Kampus IPB Baranangsiang, Bogor, Indonesia

^3^ Department of Chemistry, Faculty of Mathematic and Natural Sciences, Bogor Agricultural University, Jl. Lingkar Akademik, Kampus IPB Darmaga, Bogor, Indonesia

Endophytic fungus RS3 isolated from epiphytic plant sarang semut (*Hydnophytum formicarum*) produced extracellular black pigment as a secondary metabolite. The research aimed to extract melanin pigment from RS3, characterize and analyze SPF activity of the pigment. This research consisted of several steps including determination of precipitation solvent, extraction, qualitative analysis and melanin characterization, SPF analysis, and toxicity analysis using Brine Shrimp Lethality Test (BSLT) method. The result showed that the pigment could be extracted in acid solvent with pH 2.5 and showed positive in melanin qualitative analysis. According to FTIR and UV-Vis analyses, pigment from RS3 was proposed to be eumelanin possessing UV-Vis spectrum at UV-A (367.8 nm and 350.0 nm), UV-B (317.2 nm) and UV-C (271.6 nm; 266.8 nm; 264.0 nm; 260.8 nm; 223.6 nm). It has also several functional groups such as hydroxy, aromatic, phenol, and amine. The level of toxicity was 557.95 µg/mL. The fotoprotector value using SPF method was 11.33.

**Keywords:** ant plant; endophytic fungi; eumelanin; SPF

#### **New Hexacyclic Polyketides from a Marine *Streptomyces*** 

**José Ma Sánchez López, Francisco Romero Millán, Marta Martínez Insua and Antonio Fernández Medarde**

Instituto Biomar, S. A, Leon, Spain

The search for new compounds with potential biological activity remains the main objective of natural products development. During the last 40 years, marine organisms have proven to be a rich source of metabolites and have been able to provide novel lead compounds for the development of new pharmaceutical agents [1]. The protein kinases include a large number of family members, which play a central role in regulating a wide variety of cellular function. In the course of a screening program for new kinase inhibitors, five hexacyclic poliketides were identified, from the mycelium of a *Streptomyces* sp., isolated from an unidentified alga collected at the Spanish’s Bay of Biscay Coast (Cantabrian Sea). Three of the compounds are new natural products, the other two are previously reported compounds [2,3]. Details of the producer microorganism, isolation and spectroscopic data leading to the structure elucidation of the compounds, as well as its biological activities will be presented.

##### ***References*** 

1. Blunt, J.W.; Copp, B.R.; Keyzers, R.A.; Munro, M.H.G.; Prinsep, M.R. Marine natural products. *Nat. Prod. Rep.*
**2013**, *30*, 237–323.
2. Herath, K.B.; Jayasuriya, H.; Guan, Z.; Schulman, M.; Ruby, C.; Sharma, N.; MacNaul, K.; Menke, J.G.; Kodali, S.; Galgoci, A.; *et al.* Anthrabenzoxocinones from *Streptomyces* sp. as Liver X receptor ligands and antibacterial agents. *J. Nat. Prod.*
**2005**, *68*, 1437–1440.
3. Kojiri, K.; Nakajima, S.; Fuse, A.; Suzuki, H.; Suda, H. BE-24566B, a new antibiotic produced by *Streptomyces violaceusniger*. *J. Antibiot.*
**1995**, *48*, 1506–1508.

### **Deep Sea and Polar Research** 

#### **Identification of Bioactivities and Bioactive Compound Structure Determination from Deep-Sea Sponge Associated Microbes** 

**Stephen A Jackson ^1^, Jonathan Kennedy ^1^, Jioji Tabudravu ^2^, Marcel Jaspers ^2^, Fergal O’Gara ^2,3^ and Alan D. W Dobson ^1^**

^1^ School of Microbiology, University College Cork, Cork, Ireland

^2^ Marine Biodiscovery Centre, Department of Chemistry, University of Aberdeen, Aberdeen, UK

^3^ Biomerit Research Centre, University College Cork, Cork, Ireland

Marine sponges (Porifera) host extensive and diverse populations of symbiotic microbes with sponges and their symbionts being the source of the largest fraction of all new marine natural products described annually. However, sponges from the deep-sea remain relatively unexplored. We sampled sponges from Irish waters in the Atlantic Ocean from depths ranging from ~750 m to 2900 m. Bacteria were isolated from sponge tissues on a range of isolation media. Actinobacterial isolates were subsequently fermented in four growth media designed to elicit production of bioactive compounds. Subsequently, culture extracts of aqueous and organic soluble compounds were prepared through chromatographic techniques and the extracts were tested for bioactivities. Significant bioactivities (anti-bacterial and anti-fungal) have been identified from 42 individual extracts. Very strong activity was observed from a further 15 individual extracts including broad-range antimicrobial activities against clinically relevant strains of bacteria and/or fungi. Scale-up fermentations of 2 bacterial strains allowed for the production of compounds of interest in sufficient quantities to allow for compound purification and structure elucidation. The known bioactive compounds, Amicoumacins B and C [1,2]; hybrid PKS-NRPS gene products were identified. Genomic analyses has now clearly established that “silent” or “cryptic” secondary metabolism biosynthesis pathways are common in Actinobacteria. We are currently using methods including genome “mining” and co-cultivation of our 15 marine Actinomycete species to uncover novel metabolites. Analysis of genome sequence data from the deep sea Actinobacteria forms part of the PharmaSea project, where we will seek to both identify gene clusters for isolated metabolites and target novel gene clusters for activation.

##### ***References*** 

1. Itoh, J.; Omotoh, S.; Nishizawa, N.; Kodama, Y.; Inouye, S. Chemical structures of Amicoumacins produced by *Bacillus pumilus*. *Agric. Biol. Chem.*
**1982**, *46*, 2659–2665.
2. Pinchuk, I.V.; Bressollier, P.; Sorokulova, I.B.; Verneuil, B.; Urdaci, M.C. Amicoumacin antibiotic production and genetic diversity of *Bacillus subtilis* strains isolated from different habitats. *Res. Microbiol.*
**2002**, *153*, 269–276.

#### **Chemical Ecology of the Antarctic Nudibranch *Charcotia granulosa*** 

**Juan Moles ^1,2^, Adele Cutignano ^2^, Angelo Fontana ^2^ and Conxita Avila ^1^**

^1^ University of Barcelona, Barcelona, Spain

^2^ Istituto di Chimica Biomolecolare, Consiglio Nazionale delle Ricerche, Pozzuoli (Napoli), Italy

Sea slugs are characterized by the presence of a wide array of natural products, often used as defense against sympatric predators. In Antarctica, even if only few species have been chemically studied, evidence has been progressively increasing that many natural products act as protective agents against predation by sea stars and other putative predators. The Antarctic nudibranch *Charcotia granulosa* Vayssière, 1906 (Mollusca: Gastropoda) was collected in Deception Island during our last cruise and analyzed for the presence of new natural products. Several adults together with some egg masses and its bryozoan prey, *Beania erecta*, were chemically analyzed by chromatographic and spectroscopic techniques. A new homosesterterpene, named granuloside (**1**), was characterized from the notum of the sea slug by spectroscopic studies on natural compound and its methyl derivatives (**1a** and **1b**). Despite the apparently simplicity, granuloside exhibits an unprecedented linear C26 skeleton that arises a few questions about its biogenesis and origin in the Antarctic nudibranch. Here we report the description of the novel natural product and discuss the key characteristics of its putative biosynthesis and function. Our results suggest that *C. granulosa* synthesizes *de novo* the compound in early stages of its ontogeny, this being the first evidence of biosynthesis within the family Charcotiidae. In fact, granuloside is the first example of terpenes reported in the family Charcotiidae and, in analogy with similar compounds from other marine and terrestrial organisms, its occurrence may be beneficial for the nudibranh and be one of the biochemical factors that have supported its radiation upon a wide geographic area around the South Pole.

## **Poster Session 2: Isolation and Structure Elucidation** 

### ***Salarin C, A Potent Inducer of Apoptosis*** 

**Yoel Kashman ^1^, Lee Zur ^1^, Ashgan Bishara ^1^, Maurice Aknin ^2^, Drorit Neumann ^3^ and Nathalie Ben-Califa ^3^**

^1^ School of chemistry, Tel Aviv University, Ramat Aviv 69978, Israel

^2^ Laboratorie de Chimîe des Substances Naturelles et des Aliments, Faculté des Sciences et Techniques, Université de Réunion, 15 Avenue Rene Cassin, B.P. 7151, 97715 Saint Denis, Cedex 9, France

^3^ Department of Cell and Developmental Biology, Tel Aviv University, Ramat Aviv 69978, Israel

Four groups of novel nitrogen atom containing compounds have been isolated from the Madagascan sponge *Fascaplysinopsis* sp. The chemical content of the various samples collected in Salary bay (100 km north to Tulear) changed significantly from one site to the other suggesting the metabolites to origin from guest micro organisms. A notion supported by resemblance of several of the compounds to earlier reported micro organism metabolites. The sponge extract was tested for its effect on proliferation of the K562 leukemia cell line. Salarin C was the most effective, among the salarins tested, in inhibiting cell proliferation, as measured by the MTT assay, exhibiting inhibitory activity down to 0.005 µg/mL. Once purified, salarin C becomes very unstable and changes under light in the air to salarin A. Possessing eight functional groups complicates the chemistry of the salarins. Next to transformation of the oxazole ring to a triacetylamine, the second sensitive moiety is the vinylepoxide. The latter becomes a good site for chemical transformations and *inter alia* may explain the biogenesis of tausalarin C. Namely, opening of the vinylepoxide of salarin A by nucleophilic attack of pre-taumycin A.

#### **New Polycyclic Xanthones Isolated from Marine *Actinomadura* sp.** 

**Librada M Cañedo, Carmen Schleissner, Ana M Peñalver and Fernando de la Calle**

Carmen Cuevas Pharmamar, Madrid 28770, Spain

Historically, terrestrial microorganisms have been a plentiful source of structurally diverse bioactive substances, and have provided important contributions to the discovery of pharmaceutically useful compounds, many of them isolated from actinomycetes [1]. Marine derived bacteria constitute a new and promising source of unique metabolites with considerable pharmaceutical and therapeutic potential [2,3], in particular, marine actinobacteria are an attractive resource for new bioactive compounds screening [4]. In the context of our interest in discovering new cytotoxic compounds from marine sources, three new polycyclic xanthones PM140035, PM140036 and PM140108, with general structure **1**, have been isolated from marine bacteria belonging to the genus *Actinomadura*. Polycyclic xanthones are a family of polyketides which are characterized by their highly oxygenated angular hexacyclic frameworks [5]. A literature search for related compounds led to the closely related core structure of IB-00208 which was also isolated from a marine derived species of *Actinomadura* [6]. Citreamycins, cervinomycins and simaomicins also contain a 1,4-dioxygenated xanthone subunit, and are structurally related to **1** but belong to the family of xanthone derivatives with a cyclic amide, which is rare amongst aromatic polyketides [7]. More details about the producer microorganism, isolation and spectroscopic data leading to the structure determination of these new polycyclic xanthones and their biological activities will be reported.

##### ***References*** 

1. Bauer, A.; Brönstrup, M. Industrial natural product chemistry for drug discovery and development. *Nat. Prod. Rep*. **2014**, *31*, 35–60.
2. Blunt, J.W.; Copp, B.R.; Keyzers, R.A.; Munro, M.H.; Prinsep, M.R. Marine natural products. *Nat. Prod. Rep*. **2015**, *32*, 116–211.
3. Molinski, T.F.; Dalisay, D.S.; Lievens, S.L.; Saludes, J.P. Drug development from marine natural products. *Nat. Rev. Drug Discov*. **2009**, *8*, 69–85.
4. Subramani, R.; Aalbersberg, W. Marine actinomycetes: An ongoing source of novel bioactive metabolites. *Microbiol. Res.*
**2012**, *167*, 571–580.
5. Winter, D.K.; Sloman, D.L.; Porco, J.A., Jr. Polycyclic xanthone natural products: Structure, biological activity and chemical synthesis. *Nat. Prod. Rep*. **2013**, *30*, 382–391.
6. Rodríguez, J.C.; Puentes, J.L.F.; Baz, J.P.; Cañedo, L.M. IB-00208, a New Cytotoxic Polycyclic Xanthone Produced by a Marine-derived *Actinomadura* II. Isolation, Physico-chemical Properties and Structure Determination. *J. Antibiot*. **2003**, *56*, 318–321.
7. Hopp, D.C.; Milanowski, D.J.; Rhea, J.; Jacobsen, D.; Rabenstein, J.; Smith, C.; Romari, K.; Clarke, M.; Francis, L.; Irigoyen, M.; *et al*. Citreamicins with Potent Gram-Positive Activity. *J. Nat. Prod.*
**2008**, *71*, 2032–2035.

#### **Pretrichodermamide *C* and *N*-methylpretrichodermamide B, Two New Cytotoxic Epidithiodiketopiperazines from Hyper Saline Lake Derived *Penicillium* sp.** 

**Raha Orfali ^1,2^, Amal Ali ^1^, Weaam Ebrahim ^1,3^, Mohamed Abdel-Aziz ^4^, Werner Muller ^5^, WenHan Lin ^6^, Georgios Daletos ^1^ and Peter Proksch ^1^**

^1^ Heinrich-Heine-University, Dusseldorf, Germany

^2^ King Saud University, Riyadh, Saudi Arabia

^3^ Mansoura University, Mansoura, Egypt

^4^ National Research Center, Cairo, Egypt

^5^ Medical Center of Johannes Gutenberg University, Mainz, Germany

^6^ Peking University, Beijing, China

The aim of this study is to isolate and structural elucidate bioactive fungal secondary metabolites from a hypersaline ecosystem. Epidithiodiketopiperazines (ETPs) are a diverse group of natural products characterized by a disulfide linkage across the dioxopiperazine ring. These metabolites possess a broad spectrum of biological activities, including antibacterial, antiviral and antifungal activities. Ascomycetes are known to accumulate numerous new and bioactive secondary metabolites including ETPs. The genus *Penicillium* comprises more than 300 known species and contains a highly diversified array of active compounds. Thousands of *Penicillium* isolates have probably already been screened in bioprospecting programmes since the discovery of penicillin, and still new biologically active secondary metabolites continue to be discovered from these fungi indicating their importance as a source for novel bioactive molecules with interest to the pharmaceutical industry. In this study, a fungal strain WN-11-1-3-1-2, identified as *Penicillium* sp., was isolated from the sediment of Wadi El-Natrun Lake (a hyper saline lake), Egypt, 80 km northwest of Cairo. The crude ethyl acetate extract of the fungus was subjected to different chromatographic techniques to yield two new epidithiodiketopiperazines (ETPs) derivatives, pretrichodermamide C (**1**) and *N*-methylpretrichodermamide B (**2**). The structures of (**1**) and (**2**) were unambiguously determined on the basis of one- and two-dimensional NMR spectroscopy and by high-resolution mass spectrometry, as well as by comparison with the literature. Compound (**2**) showed pronounced cytotoxicity against the murine lymphoma L5178Y cell line with an IC_50_ value of 2 mM. The results presented here suggest that halotolerant fungi from hypersaline environments are a rich source of bioactive secondary metabolites which could have implications for drug discovery in the future.

#### **Antimalarial Endoperoxide Polyketides from the Chinese Marine sponge *Plakortis* cfr. *simplex*** 

**Giuseppina Chianese ^1^, Marco Persico ^1^, Fan Yang ^2^, Hou-Wen Lin ^2^, Nicoletta Basilico ^3^, Silvia Parapini ^4^, Donatella Taramelli ^4^, Orazio Taglialatela-Scafati ^1^ and Caterina Fattorusso ^1^**

^1^ Department of Pharmacy, University of Naples Federico II, Naples, Italy

^2^ Key Laboratory for Marine Drugs, Department of Pharmacy, Renji Hospital, School of Medicine, Shanghai Jiao Tong University, Shanghai, China

^3^ Dipartimento di Scienze Biomediche, Chirurgiche e Odontoiatriche, Università di Milano, Milan, Italy

^4^ Dipartimento di Scienze Farmacologiche e Biomolecolari Università di Milano, Milan, Italy

Marine sponges of the genus *Plakortis* (Demospongiae, Plakinidae) have been intensively investigated for their secondary metabolites over the last decades. The most prominent and peculiar class of *Plakortis* metabolites is given by propionate- and butyrate-based polyketides, exemplified by the simple 1,2-dioxane plakortin [1,2], often endowed with a promising antimalarial activity [3]. An intense research activity on *Plakortis* metabolites led to postulate a likely mechanism of action [4], basing on the information coming from natural and synthetically prepared analogues. Chemical investigation of the organic extract obtained from the sponge *Plakortis simplex *collected in the South China Sea afforded five new polyketide endoperoxides four of which containing a 1,2-dioxene ring, never evaluated before for antimalarial activity. The stereostructures of these metabolites have been deduced on the basis of spectroscopic analysis and chemical conversion. The isolated endoperoxide derivatives have been tested for their *in vitro* antimalarial activity against *Plasmodium falciparum *strains, showing IC_50_ values in the low micromolar range. Other apolar fractions of the same organic extract afforded novel polyketide-based metabolites, testifying the incredible chemical diversity produced by this organism. Some examples will be provided in this presentation.

##### ***References*** 

1. Higgs, M.D.; Faulkner, D. John. Plakortin, an antibiotic from Plakortis halichondrioides. *J. Org. Chem.*
**1978**, *43*, 3454–3457.
2. Fattorusso, E.; Taglialatela-Scafati, O; Di Rosa, M; Ianaro, An. Metabolites from the Sponge *Plakortis simplex*. Part 3: Isolation and Stereostructure of Novel Bioactive Cycloperoxides and Diol Analogues. *Tetrahedron*
**2000**, *56*, 7959–7967.
3. Fattorusso, E.; Parapini S.; Campagnuolo, C.; Basilico, N.; Taglialatela-Scafati, O; Taramelli, D. Activity against *Plasmodium falciparum* of cycloperoxide compounds obtained from the sponge *Plakortis simplex*. *J. Antimicrob. Chemother.*
**2002**, *50*, 883–888.
4. Taglialatela-Scafati, O.; Fattorusso, E.; Romano, A,; Scala, F.; Barone, V.; Cimino, P.; Stendardo, E.; Catalanotti,B.; Persicoa, M.; Fattorusso, C. Insight into the mechanism of action of plakortins, simple 1,2-dioxane antimalarials. *Org. Biomol. Chem.*
**2010**, *8*, 846–856.

#### **New 36-Membered Antifungal Macrolides from *Streptomyces* caniferus** 

**Rodney Lacret, Daniel Oves-Costales, Caridad Díaz, Ignacio Pérez-Victoria, Jesús Martín, Mercedes de la Cruz, Nuria de Pedro, Francisca Vicente and Olga Genilloud**

Fundación Medina, Armilla, Granada, Spain

Macrolides constitute one of the most interesting groups of natural products, mainly produced by actinomycetes and fungi. They exhibit various biological activities, including antitumor, antifungal, antiparasitic, citotoxic and immunosuppressant activities. Bioactive macrolides containing a 32 or 36-membered macrocyclic lactones have been described in the past and include, among others, brasilinolide, liposidolide, and novonestmycins A and B. However, macrolides linked to sugar derivatives and a 1,4-naphtoquinone are fairly uncommon. At present, they are restricted to the axenomycins and langkolide, isolated from culture broths of *Streptomyces lisandri* and *Streptomyces* sp. Acta 3062. As part of the PharmaSea project, over 400 actinomycetes were grown on carefully selected fermentation media, and their fermentation extracts were assayed against clinically relevant pathogenic microbial strains. In this work, we detected that acetone extracts from culture broths of *Streptomyces caniferus* (CA-271066) possesed antifungal activity against *Aspergillus fumigatus* and *Candida albicans*. Herein we report the bioassay guided isolation, structural elucidation, antifungal and citotoxic properties of a family of new macrolides (structurally related to axenomycins) isolated from culture broths of this actinomycete. The producing strain was isolated from an *Ascidia* collected off-shore in Sao Tome and Principe and it was grown in APM9-modified medium. The extraction of culture broths with acetone afforded an aqueous crude extract (ACE), which displayed antifungal activity. Liquid-liquid extraction of ACE with ethyl acetate followed by reversed-phase semipreparative HPLC yielded several new 36-membered antifungal macrolides as the compounds responsible for the observed bioactivity. Structural elucidation of the family of compounds was based on 1D and 2D NMR and High Resolution Mass Spectrometry (ESI-TOF). Their relative stereochemistry was determined by* J*-based configuration analysis combined with the presence of key NOESY correlations and data reported for similar compounds.

#### **Novel Adociaquinone Derivatives from the Indonesian Marine Sponge *Xestospongia* sp.** 

**Fei He ^1^, Linh H. Mai ^1^, Arlette Longeon ^1^, Brent R. Copp ^2^, Nadège Loaëc ^3^, Amandine Bescond ^3^, Laurent Meijer ^3^ and Marie-Lise Bourguet-Kondracki ^1^**

^1^ Laboratoire Molécules de Communication et Adaptation des Micro-organismes, UMR 7245 CNRS/MNHN, Muséum National d’Histoire Naturelle, 57 rue Cuvier (C.P. 54), 75005 Paris, France

^2^ School of Chemical Sciences, University of Auckland, Private Bag 92019, Auckland 1142, New Zealand

^3^ ManRos Therapeutics, Perharidy Research Center, 29680 Roscoff, France

Marine sponges of the genus* Xestospongia* have proved to be an extremely rich source of secondary metabolites with unprecedented molecular structures and various bioactivities. Adocia-, halena- and xesto-quinone are the three main quinone-type skeletons identified from sponges of the genus *Xestospongia*. Among the most significant compounds, adociaquinones A and B, first isolated from the sponge *Adocia* sp. and then from the Philippine sponge *Xestospongia* sp. revealed inhibition of topoisomerase II in catalytic DNA unwinding and decatenation assays as well as inhibition of enzyme in the potassium sodium dodecyl sulfate assay. Previous investigations on the South Pacific *Xestospongia* sp. by our group led to the isolation of a series of halenaquinone-type compounds, including xestosaprol C methylacetal, 3-ketoadociaquinones A and B, tetrahydrohalenaquinones A and B, halenaquinol sulfate, halenaquinone and orhalquinone. Orhalquinone demonstrated significant inhibitory activities against both human and yeast farnesyltransferase enzymes, with IC_50_ values of 0.4 μM [1]. In the frame of the European Bluegenics program, we have chemically investigated the Indonesian sponge of *Xestospongia* sp. collected off North Sulawesi because its methanol crude extract had showed kinase inhibition as well as antimicrobial and antioxidant activities. Bio-guided fractionation of the extract led to the isolation of seven new adociaquinone derivatives **1a**–**4c**, together with seven known compounds, adociaquinone A and B, secoadociaquinones A and B, 15-chloro-14-hydroxyxestoquinone, 14-chloro-15-hydroxyxestoquinone and xestoquinol sulfate. The known compounds were identified by comparison of their spectroscopic data with those of the literature. The isolation and structural elucidation of the new compounds as well as their biological activities will be presented and discussed [2].

##### ***References*** 

1. Longeon, A.; Copp, B.R.; Roué, M.; Dubois, J.; Valentin, A.; Petek, S.; Debitus, C.; Bourguet-Kondracki, M.-L. New bioactive halenaquinone derivatives from South Pacific marine sponges of the genus *Xestospongia*. *Bioorg. Med. Chem*. **2010**, *18*, 6006–6011.
2. He, F.; Mai, L.H.; Longeon, A.; Copp, B.R.; Loaëc, N.; Bescond, A.; Meijer, L.; Bourguet-Kondracki, M.-L. Novel Adociaquinone Derivatives from the Indonesian Sponge *Xestospongia* sp.* Mar. Drugs*
**2015**, *13*, 2617–2628.

#### **Erythrins, New Toxic Metabolites from the Marine Ciliate *Pseudokeronopsis erythrina* Used as Chemical Defense against Predators** 

**Andrea Anesi ^1^, Federico Buonanno ^2^, Graziano DiGiuseppe ^3^, Claudio Ortenzi ^2^ and Graziano Guella ^1^**

^1^ University of Trento, Trento, Italy

^2^ University of Macerata, Macerata, Italy

^3^ University of Pisa, Pisa, Italy

Marine protozoa are known for their ability to produce a vast and chemically diverse array of secondary metabolites that are involved in different ecological functions. Morphospecies belonging to genus Euplotes have been extensively studied for their ability to produce chemically diverse secondary metabolites and, interestingly, it was found that strains belonging to same genetic clades were characterized by a different profile of bioactive compounds [1]. From the genus *Pseudokeronopsis* only two classes of pigments have been so far isolated, keronopsins as defensive molecules of *Pseudokeronopsis rubra* [2] and, more recently, keronopsamides from cell culture of the marine ciliate *Pseudokeronopsis riccii* [3]. We report here on the characterization of new secondary metabolites, erythrins, produced by cell cultures of *Pseudokeronopsis erythrina* (Ciliophora, Hypotricha). Their structure have been elucidated by extensive NMR and high resolution MS measurements and are characterized by a central 4-hydroxy-unsaturated delta-lactone ring bearing an alkyl saturated chain at C(2) and a butyl -benzenoid group at C(5). The simultaneous presence of the corresponding 4-sulphate analogues has also been ascertained and a reasonable proposal of their biosynthesis will be reported. Cold-shock treatment has been performed to induce the discharge of these metabolites from cell pigment granules. The analysis of cytotoxic activity on a panel of free-living ciliates and micro-invertebrates, together with some observation on the defensive behavior by *P. erythrina*, indicated that erythrins are very effective for its chemical defence.

##### ***References*** 

1. Guella, G.; Skropeta, D.; di Giuseppe, G.; Dini, F. Structures, biological activities and phylogenetic relationships of terpenoids from marine ciliates of the genus *Euplotes*. *Mar. Drugs*
**2010**, *8*, 2080–2116.
2. Höfle, G.; Pohlan, S.; Uhlig, G.; Kabbe, K.; Schumacher, D. Keronopsins A and B, Chemical Defence Substances of the Marine Ciliate *Pseudokeronopsis rubra* (Protozoa): Identification by *Ex Vivo *HPLC. *Angew. Chem. Int. Ed. Engl.*
**1994**, *33*, 1495–1497.
3. Guella, G.; Frassanito, R.; Mancini, I.; Sandron, T.; Modeo, L.; Verni, F.; Dini, F.; Petroni, G. Keronopsamides, a new class of pigments from marine ciliates. *Eur. J. Org. Chem.*
**2010**, *2010*, 427–434.

#### **Cyclic Peptides from the Indonesian Marine Sponge *Callyspongia aerizusa* with Potent and Selective Antitubercular Activity** 

**Georgios Daletos ^1^, Rainer Kalscheuer ^2^, Rudolf Hartmann ^3^ and Peter Proksch ^1^**

^1^ Institute for Pharmaceutical Biology and Biotechnology, Heinrich-Heine-University, Duesseldorf, Germany

^2^ Institute for Medical Microbiology and Hospital Hygiene, Heinrich-Heine-University, Duesseldorf, Germany

^3^ Institute of Complex Systems: Strukturbiochemie, Forschungszentrum Juelich, Juelich, Germany

Chemical investigation of the Indonesian sponge *Callyspongia aerizusa* afforded 13 cyclic peptide derivatives, namely callyaerins. The planar structures of the isolated compounds were unambiguously elucidated on the basis of 1D and 2D NMR spectroscopic data and MS interpretation. The absolute configurations of their constituent amino acid residues were determined using Marfey’s method. The basic structural unit of the callyaerins comprises a cyclic peptide with a linear peptide side chain, both of variable size, linked through a non-proteinogenic (*Z*)-2,3-diaminoacrylic acid (DAA) functional group. The peptides are unusual in containing a considerable number of proline residues, of which one proline is always positioned at the beginning of the side chain, while all others are found in the ring system. All compounds were investigated *in vitro* against *Mycobacterium tuberculosis*, as well as against THP-1 (human acute monocytic leukemia), and MRC-5 (human fetal lung fibroblast) cell lines in order to assess their general cytotoxicity. Callyaerins were found to inhibit *M. tuberculosis* at low micromolar concentrations making these compounds interesting candidates for further studies.

#### **Epipyrones from the Marine-Derived Fungus Epicoccum Nigrum Link Inhibit the Proteases Cathepsin K and S** 

**Peter Hufendiek ^1^, Stefan Kehraus ^1^, Michael Gütschow ^2^ and Gabriele M. König ^1^**

^1^ Institute of Pharmaceutical Biology, University of Bonn, Bonn, Germany

^2^ Institute of Pharmaceutical Institute, Pharmaceutical Chemistry I, University of Bonn, Bonn, Germany

Fungi belonging to the Ascomycota are an excellent source of bioactive compounds, among others, antibiotics, immunosuppressants and cholesterol lowering agents. This project aims to identify new fungal secondary metabolites useful as lead structures in pharmacology, especially compounds which show enzyme inhibitory activity. Since the marine environment and fungi occurring there are not yet well researched concerning the presence of pharmacologically active compounds, our project targets fungi isolated from marine algae. Here we focus on a marine-derived strain of the fungus Epicoccum nigrum, isolated from the surface of a green alga. Cultivation and fractionation of the crude extract led to the isolation of the epipyrones, which are isomeric polyketides with an unusual *C*-glycosyl-moiety. Biological testing revealed selective activity against two different cysteine proteases,* i.e.*, cathepsin K and S. The IC_50_-values are 11.4 and 6.6 μM, respectively. Cathepsin K inhibitors may be used to combat osteoporosis, whereas cathepsin S plays a role in tumor proliferation. Interestingly, no activity was seen against cathepsin B and L. For the serine proteases human leukocyte elastase and trypsin, lower or no activity was measured, underlining the selectivity of the compounds. Since the epipyrones tend to isomerize quickly, we aimed to block isomerization by acetylation of the glycosyl-unit. Indeed, acetylation led to one single isomer, which will also be tested against the aforementioned targets, to compare the inhibitory activity. In conclusion, the epipyrones were shown to exhibit selective activity against cathepsin K and S. The role of these enzymes in osteoporosis and tumor growth renders the epipyrones interesting lead compounds for further studies.

##### ***Acknowledgments*** 

This research is funded by the NRW International Graduate Research School BIOTECH-PHARMA.

#### **Isocoumarins and Cyclic Hexapeptide from the Sponge-Associated Fungus *Aspergillus similanensis* sp. nov. Kufa 0013** 

**Chadaporn Prompanya ^1^, Tida Dethoup ^2^, Madalena Pinto ^3^ and Anake Kijjoa ^1^**

^1^ Instituto de Ciências Biomédicas Abel Salazar and CIIMAR, Universidade do Porto, Porto, Portugal

^2^ Department of Plant Pathology, Faculty of Agriculture, Kasetsart University, Bangkok, Thailand

^3^ Laboratório de Química Orgânica e Farmacêutica, Departamento de Ciências Químicas, Faculdade de Farmácia, Universidade do Porto, Porto, Portugal

In our ongoing search for new natural products with antibacterial and anticancer activities produced by the marine-derived fungi of the genera *Neosartorya *and *Aspergillus*, we have investigated the secondary metabolites of a Thai collection of a new species of *Aspergillus*, which we have named *Aspergillus similanensis *(KUFA0013), isolated from the marine sponge *Rhabdermia *sp., collected from the Similan Islands in Southern Thailand. The ethyl acetate extract of the culture of this fungus furnished several isocoumarine derivatives including new isocoumarins similanpyrones A (**1**), B (**2**) and C (**3**), a new chevalone analog, chevalone E (**4**), a new pyripyropene analog, pyripyropene T (**5**), and a new cyclic peptide, named similanamide (**6**) [1,2]. Some of the isolated compounds were evaluated for their antimicrobial activity against Gram-positive and Gram-negative bacteria and multidrug-resistant isolates from the environment.

##### ***Acknowledgments*** 

This work was partially supported by the Project MARBIOTECH (reference NORTE-07-0124-FEDER-000047).

##### ***References*** 

1. Prompanya, C.; Dethoup, T.; Bessa, L.J.; Pinto, M.M.M.; Gales, L.; Costa, P.M.; Silva, A.M.S.; Kijjoa, A. New Isocoumarin Derivatives and Meroterpenoids from the Marine Sponge-Associated Fungus *Aspergillus similanensis* sp. nov. KUFA 0013 *Mar. Drugs*
**2014**, *12*, 5160–5173.
2. Prompanya, C.; Fernandes, C.; Cravo, S.; Pinto, M.M.M.; Dethoup, T.; Silva, A.M.S.; Kijjoa, A. A New Cyclic Hexapeptide and a New Isocoumarin Derivative from the Marine Sponge-Associated Fungus *Aspergillus similanensis* KUFA 0013. *Mar. Drugs*
**2015**, *13*, 1432–1450.

#### **Indole Alkaloids and Dihydroisocoumarin from the Alga-Associated Fungus *Neosartorya takakii* KUFC 7898** 

**War War May Zin ^1^, Suradet Buttachon ^1^, Jamrearn Buaruang ^2^ and Anake Kijjoa ^1^**

^1^ Instituto de Ciências Biomédicas Abel Salazar and CIIMAR, Universidade do Porto, Porto, Portugal

^2^ Division of Environmental Science, Faculty of Science, Ramkhamhaeng University, Bangkok, Thailand

In our ongoing search for new natural products with antibacterial activity produced by the marine-derived fungi of the genus *Neosartorya *[1,2], we have investigated the secondary metabolites of a Thai collection of *Neosartorya takakii* KUFC 7898, isolated from the marine alga *Amphiroa *sp., collected from Samaesarn Island in the Gulf of Thailand. The ethyl acetate extract of its culture furnished so far, besides the indole alkaloids aszonalenin (**1a**), acetylaszonalenin (**1b**), tryptoquivalines F (**2a**), H (**2b**), L (**2c**) and a new tryptoquivaline derivative which we have named tryptoquivaline U (**3d**), aszonapyrone A (**3**) and a dihydroisocoumarin derivative, 6-hydroxymellein (**4**). The structures of the compounds were established by 1D and 2D NMR spectral analysis and HRMS. All the compounds were evaluated for their antibacterial activity against Gram positive (*Staphylococcus aureus *ATCC 25923 and *Bacillus subtilis *ATCC 6633) and Gram negative (*Escherichia coli *ATCC 25922 and *Pseudomonas aeruginosa *ATCC 27853) bacteria as well as multidrug-resistant isolates from the environment.

##### ***Acknowledgments*** 

This work was partially supported by the Project MARBIOTECH (reference NORTE-07-0124-FEDER-000047).

##### ***References*** 

1. Eamvijarn, A.; Nelson M. Gomes, N.M.; Dethoup, T.; Buaruang, J.; Manoch, L.; Silva, A.; Pedro, M.; Marini, I.; Roussis, V.;* et al.* Bioactive meroditerpenes and indole alkaloids from the soil fungus *Neosartorya fischeri* (KUFC 6344), and the marine-derived fungi *Neosartorya laciniosa* (KUFC 7896) and *Neosartorya tsunodae* (KUFC 9213). *Tetrahedron*
**2013**, *69*, 8583–8591.
2. Gomes, N.M.; Bessa, L.J.; Buttachon, S.; Costa, P.M.; Buaruang, J.; Dethoup, T.; Silva, A.M.S.; Kijjoa, A. Antibacterial and Antibiofilm Activities of Tryptoquivalines and Meroditerpenes Isolated from the Marine-Derived Fungi *Neosartorya paulistensis*, *N. laciniosa*, *N. tsunodae*, and the Soil Fungi *N. fischeri* and *N. siamensis*. *Mar. Drugs*
**2014**, *12*, 822–839.

#### **Anti-Inflammatory Activity of Tanzawaic Acid Derivatives from a Marine-derived Fungus *Penicillium steckii* 108YDC142** 

**Chien Fang, Soo-Jin Heo, Hyi-Seung Lee, Yeon-Ju Lee, Jong Seok Lee and Hee Jae Shin**

Korea Institute of Ocean Science and Technology, Ansan, Korea

Chemical investigation of a marine-derived fungus *Penicillium steckii* 108YDC142, isolated from a marine sponge sample collected at Wangdolcho, East Sea, Korea, resulted in the discovery of a new tanzawaic acid derivative (**1**), together with four known analogues, tanzawaic acids A (**2**), C (**3**), D (**4**), and K (**5**). Their structures were determined by the detailed analysis of NMR and MS data, along with chemical methods. These compounds significantly inhibited the nitric oxide (NO) production and the new tanzawaic acid derivative (**1**) inhibited the lipopolysaccharide (LPS)-induced inducible nitric oxide synthase (iNOS) and cyclooxygenase-2 (COX-2) proteins and mRNA expressions in RAW 264.7 macrophages. Additionally, compound **1** reduced the mRNA levels of inflammatory cytokines, including tumor necrosis factor-α, interleukin (IL)-1β, and IL-6. Taken together, the results of this study demonstrate that the new tanzawaic acid derivative inhibits LPS-induced inflammation.

#### **Nocardiomycins A–C, New Cytotoxic Cyclic Depsipeptides Isolated from Marine *Actinomycetes*** 

**Marta Pérez, Cristina Lillo, Rogelio Fernández, Librada Cañedo, Paz Zuñiga, Pilar Rodríguez** **, Simon Munt and Carmen Cuevas**

Pharmamar, Colmenar Viejo/Madrid, Spain

Cyclic depsipeptides have emerged as a very important class of bioactive compounds from marine derived bacteria. Several of these compounds have been disclosed to have cytotoxic, antiviral and/or antifungal properties. Specifically, rakicidins A, B and D [1–4] showed cytotoxic activity against the murine carcinoma colon 26-L5 tumour cells, and vinylamycin [5,6], exhibited antimicrobial activities against Gram-positive bacteria including MRSA. In an effort to discover new cytotoxic compounds from marine sources, three new lipopeptides named nocardiomycin A (**1**), nocardiomycin B (**2**) and nocardiomycin C (**3**) have been isolated from different marine bacteria belonging to the order Actinomycetales. These compounds, structurally related to the reported rakicidins, are 15-membered depsipeptides consisting of three amino acids and a 3-hydroxyfatty acid. One of these amino acids corresponds to the rare unusual 4-amino-2,4-pentadienoate, only found in a few examples of secondary metabolites of Actinomycetes. Details of the producer microorganisms, isolation and spectroscopic data leading to the structure determination of these new cytotoxic compounds, as well as their biological properties will be presented.

##### ***References*** 

1. McBrien, K.D.; Berry, R.L.; Lowe, S.E.; Neddermann, K.M.; Bursuker, I.; Huang, S.; Klohr, S.E.; Leet, J.E. Rakicidins, New Cytotoxic Lipopeptides from *Micromonospora* sp. Fermentation, Isolation and Characterization. *J. Antibiot.*
**1995**, *48*, 1446–1452.
2. Igarashi, Y.; Shimasaki, R.; Miyanaga, S.; Oku, N.; Onaka, H.; Sakurai, H.; Saiki, I.; Kitani, S.; Nihira, T.; Wimonsiravude, W. *et al*. Rakicidin D, an inhibitor of tumor cell invasion from marine-derived *Streptomyces* sp. *J. Antibiot.*
**2010**, *63*, 563–565.
3. Oku, N.; Matoba, S.; Yamazaki, Y.M.; Shimasaki, R.; Miyanaga, S.; Igarashi, Y. Complete Stereochemistry and Preliminary Structure-Activity Relationship of Rakicidin A, a Hypoxia-Selective Cytotoxin from *Micromonospora* sp. *J. Nat. Prod.*
**2014**, *77*, 2561–2565.
4. Sang, F.; Li, D.; Sun, X.; Cao, X.; Wang, L.; Sun, J.; Sun, B.; Wu, L.; Yang, G.; Chu, X.; *et al*. Total Synthesis and Determination of the Absolute Configuration of Rakicidin A. *J. Am. Chem. Soc.*
**2014**, *136*, 15787–15791.
5. Igarashi, M.; Shida, T.; Sasaki, Y.; Kinoshita, N.; Naganawa, H.; Hamada, M.; Takeuchi, T. Vinylamycin, a new depsipeptide antibiotic, from *Streptomyces* sp. *J. Antibiot.*
**1999**, *52*, 873–879.
6. Carr, G.; Poulsen, M.; Klassen, J.L.; Hou, Y.; Wyche, T.P.; Bugni, T.S.; Currie, C.R.; Clardy, J. Microtermolides A and B from Termite-Associated *Streptomyces* sp. and Structural Revision of Vinylamycin. *J*.* Org. Lett.*
**2012**, *14*, 2822–2825.

#### **New Cyclopeptides Isolated from *Lissoclinum patella*** 

**Rogelio Fernández, Elena Gómez, Marta Pérez and Carmen Cuevas**

Pharmamar, Colmenar Viejo/Madrid, Spain

Ascidians from the genus *Lissoclinum* are a rich source of a variety of cytotoxic cyclic peptides characterized by the presence of oxazole and thiazole moieties. Some of these peptides contain threonine and serine residues whose side chains have been modified as dimethylallyl ethers. They include the cyclopeptides Nairaiamide, Patellin, Trunkamide A, Mollamide, Hexamollamide and Comoramide [1]. In the course of our screening program to isolate novel compounds with antitumor properties from marine sources, we have isolated two new cyclopeptides, named Wetamide A and B from the ascidian *Lissoclinum patella* collected off the coast of Wetar in the Southwest islands of Indonesia. These compounds were obtained by bioassay-guided fractionation of an organic extract of the organism, using VLC RP-18 chromatography and reverse phase semi-preparative HPLC. Structure elucidation of these new metabolites was carried out by spectroscopic methods including MS, ^1^H, ^13^C and 2D-NMR. The stereochemistry of the amino acids was determined by hydrolysis followed by derivatization with Marfey’s reagent and comparison with commercial standards by HPLC-MS [2].

##### ***Acknowledgments*** 

Udayana University of Bali. Indonesia. Sebastiano Gulinello (Expedition Department Logistic Coordinator). Ministry of Marine Affairs and Fisheries. Republic of Indonesia.

##### ***References*** 

1. Blunt, J.W.; Copp, B.R.; Keyzers, R.A.; Munro, M.H.G.; Prinsep, M.R. Marine natural products. *Nat. Prod. Rep.*
**2015**, *32*, 116–211, and previous papers in this series.
2. Marfey, P. Determination of d-amino acids. II. Use of a bifunctional reagent, 1,5-difluoro-2,4-dinitrobenzene. *Carlsberg. Res. Commun.*
**1984**, *49*, 591–596.

#### **Chemical Studies of a Sample of *Hexadella* sp. from the Arafura Sea (Indonesia)** 

**Rogelio Fernández, Patricia Gema Cruz, Marta Perez and Carmen Cuevas**

Pharmamar, Colmenar Viejo/Madrid, Spain

Marine sponges of the order Verongida are a rich source of brominated tyrosine metabolites, many of which have exhibited diverse biological activities [1–4]. Chemical modification occurs both on the side chain and aromatic ring of the brominated tyrosine precursors giving rise to a broad range of biosynthetically related compounds. In the course of our screening program to search for new antitumour compounds from marine organisms, we have isolated two new bromotyrosine derivatives PM140657 (**1**) and PM140674 (**2**), as well as the known pseudoceratin A (**3**) [5], from a sample of *Hexadella* sp. collected off the coast of Arafura (Indonesia). The structures of all the compounds were elucidated by analysis of their 1D and 2D-NMR spectra and comparison with data reported for other bromotyrosine derivatives. PM140674 exhibits micromolar cytotoxicity against several cell lines, including lung (A549), colon (HT29), breast (MDA-MB-231) and pancreas (PSN1).

##### ***Acknowledgments*** 

Udayana University of Bali. Indonesia. Sebastiano Gulinello (Expedition Department Logistic Coordinator). Ministry of Marine Affairs and Fisheries. Indonesia.

##### ***References*** 

1. Blunt, J.W.; Copp, B.R.; Keyzers, R.A.; Munro, M.H.G.; Prinsep, M.R. Marine natural products. *Nat. Prod. Rep.*
**2015**, *32*, 116–211, and previous papers in this series.
2. Xu, M.; Andrews, K.T.; Birrell, G.W.; Tran, T.L.; Camp, D.; Davis, R.A.; Quinn, R.J. Psammaplysin H, a new antimalarial bromotyrosine alkaloid from a marine sponge of the genus *Pseudoceratina*. *Bioorg. Med. Chem. Lett.*
**2011**, *21*, 846–848.
3. Shaker, K.H.; Zinecker, H.; Ghani, M.A.; Imhoff, J.F.; Schneider, B. Bioactive metabolites from the sponge *Suberea* sp. *Chem. Biodivers.*
**2010**, *7*, 2880–2887.
4. Buchanan, M.S.; Carroll, A.R.; Wessling, D.; Jobling, M.; Avery, V.M.; Davis, R.A.; Feng, Y.; Hooper, J.N.A.; Quinn, R.J. Clavatadines C–E, Guanidine Alkaloids from the Australian Sponge *Suberea clavata*. *J. Nat. Prod.*
**2009**, *72*, 973–975.
5. Jang, J.-H.; van Soest, R.W.; Fusetani, N.; Matsunaga, S. Pseudoceratins A and B, antifungal bicyclic bromotyrosine-derived metabolites from the marine sponge *Pseudoceratina purpurea*. *J. Org. Chem.*
**2007**, *72*, 1211–1217.

#### **Bioactive Metabolites from Marine-Derived Actinobacteria from the East Mediterranean** 

**Panagiota Georgantea ^1^, Leto-Aikaterini Tziveleka ^1^, Eleni Mavrogonatou ^2^, Eniko Rab ^1,3^, Dimitris Kekos ^3^, Harris Pratsinis ^2^, Dimitris Kletsas ^2^, Vassilios Roussis ^1^ and Efstathia Ioannou ^1^**

^1^ Department of Pharmacognosy and Chemistry of Natural Products, Faculty of Pharmacy, University of Athens, Athens, Greece

^2^ Laboratory of Cell Proliferation and Aging, Institute of Biosciences and Applications, National Centre for Scientific Research “Demokritos”, Athens, Greece

^3^ Biotechnology Laboratory, School of Chemical Engineering, National Technical University of Athens, Athens, Greece

The screening of microbial natural products represents an important route to the discovery of novel anticancer and antibiotic agents. Diverse actinobacteria isolated from unique ecosystems have been shown to produce bioactive compounds which exert their influence by processes that are not compromised by existing multidrug-resistance pathways. In order to obtain new strains likely to produce novel metabolites, examination of samples from different habitats and extreme environments is necessary. The East Mediterranean basin is a geomorphologically and biologically unique marine ecosystem that has not been investigated so far for its microbiota as producers of secondary metabolites. In search of new bioactive secondary metabolites from marine microorganisms found in the Greek seas, we have selectively isolated more than 900 actinobacterial strains from sediments and macroorganisms from the Aegean and the Ionian Seas. On the basis of preliminary screening of the chemical profiles of small scale liquid cultures of numerous actinobacterial strains with LC-DAD-MS and NMR, in conjunction with evaluation of their antibacterial and cytotoxic activities, strains BI0048 and BI0383 were selected for further chemical investigation. The large scale liquid cultures of strains BI0048 and BI0383 afforded two crude extracts which were subjected to multi-step fractionations that have led so far to the isolation of 24 and 16 compounds, respectively. Among these, two polyketides are new natural products. The structure elucidation and the assignment of the relative configurations of the compounds were based on analyses of their spectroscopic data and comparison with literature data. The isolated metabolites are currently being evaluated for their antibacterial and cytotoxic activities.

##### ***Acknowledgments*** 

This work was supported by the project aristeia-2587 “biomaract”, which is implemented under the “aristeia” action of the operational programme “education and lifelong learning” and is co-funded by the European Social Fund (ESF) and national resources.

#### **Prevezanes and Related Diterpenes from *Laurencia glandulifera* and Evaluation of Their Anti-Inflammatory Activity** 

**Maria Harizani ^1^, Gerasimos Konidaris ^1^, Maria Daskalaki ^2^, Sotirios Kampranis ^3^, Christos Tsatsanis ^2^, Vassilios Roussis ^1^ and Efstathia Ioannou ^1^**

^1^ Department of Pharmacognosy and Chemistry of Natural Products, Faculty of Pharmacy, University of Athens, Athens, Greece

^2^ Laboratory of Clinical Chemistry, University of Crete Medical School, Heraklion, Greece

^3^ Mediterranean Agronomic Institute of Chania, Chania, Greece

Despite the extensive investigations on the chemical profile of numerous *Laurencia* species, this cosmopolitan genus still represents a prolific source of metabolites, often exhibiting antibacterial, antifungal, insecticidal and/or cytotoxic activities. *Laurencia* biosynthesizes a wide spectrum of secondary metabolites, including sesquiterpenes, diterpenes, triterpenes and C_15_ acetogenins which are frequently characterized by the presence of halogen atoms. Previous investigations on *Laurencia glandulifera* have led to the isolation of the brominated diterpene neorogioltriol which has exhibited very significant levels of analgesic and anti-inflammatory activity [1]. In search of new congeners, specimens of *L. glandulifera *were collected in Kefalonia Island in the Ionian Sea, Greece, at a depth of 1–2 m in April of 2014. Extraction of the fresh algal tissues with mixtures of CH_2_Cl_2_/MeOH afforded a residue that was subjected to a series of chromatographic separations. Eight diterpenes of the prevezane skeleton have been isolated until now. Among these, one metabolite features a new carbon skeleton. The structures and relative configurations of the isolated natural products were determined on the basis of extensive analysis of their 1D and 2D NMR and MS data. The isolated metabolites are currently being evaluated for their anti-inflammatory activity by a cell-based assay which measures the extent of macrophage activation by bacterial lipopolysaccharide (LPS).

##### ***Acknowledgments*** 

This work was supported by the project GSRT-EPANII-11ΣYN-3-770 “NRG”, which is implemented under the “Cooperation 2011” Action of the Operational Programme “Competitiveness and Entrepreneurship” and is co-funded by the European Social Fund (ESF) and National Resources

##### ***Reference*** 

1. Chatter, R.; Kladi, M.; Tarhouni, S.; Maatoug, R.; Kharrat, R.; Vagias, C.; Roussis, V. Neorogioltriol: A brominated diterpene with analgesic activity from *Laurencia glandulifera*. *Phytochem. Lett.* **2009**, *2*, 25–28.

#### **New Cytotoxic Sesquiterpenes from the Brazilian Red Alga *Laurencia catarinensis*** 

**Miriam Falkenberg ^1,2^, Efstathia Ioannou ^1^, Cintia Lhullier ^1,2^, Tauana Wanke ^1,2^, Ana Claudia Philippus ^2^, Lucas F.O. Vieira ^2^, Panagiota Papazafiri ^3^ and Vassilios Roussis ^1^**

^1^ Department of Pharmacognosy and Chemistry of Natural Products, Faculty of Pharmacy, Athens, Greece

^2^ Laboratory of Medicinal Chemistry and Natural Products, Federal University of Santa, Florianópolis, Brazil

^3^ Department of Animal and Human Physiology, Faculty of Biology, School of Science, Athens, Greece

The complex *Laurencia *(Rhodomelaceae) includes species with a wide distribution throughout the world and represents a prolific source of new secondary metabolites. Besides fulfilling ecological needs of the seaweeds, the halogenated terpenes and acetogenins that are frequently encountered in *Laurencia *species, exhibit a wide range of pharmacological activities, e.g., antiinflammatory, cytotoxic, and antibacterial. Previous studies on *Laurencia *species have indicated that the chemodiversity observed in the species level may be affected by environmental factors. In the course of our ongoing investigations towards the isolation of bioactive marine metabolites, we have previously investigated the chemical composition of *Laurencia catarinensis* Cordeiro-Marino & Fujii, collected off Ilha do Arvoredo, Santa Catarina, Southern Brazil [1] and reported the isolation of seven new and seven known metabolites, most of them structurally related to caespitol. Recent collections of this species off Ilha do Xavier from the state of Santa Catarina afforded a crude extract with a different chemical profile. In order to study the magnitude of the difference in the chemical profiles, extracts obtained from collections performed from Ilha do Arvoredo and Ilha do Xavier were investigated in depth, leading to the isolation of a number of minor metabolites. Herein, we report the isolation and structure elucidation of nine new and 14 known metabolites reported for the first time from *L. catarinensis*. Among the two different populations, only four metabolites were found as common constituents. The isolated compounds were evaluated for their *in vitro* cytotoxicity against three human tumor cell lines, namely HT29, MCF7, and A431, exhibiting variable levels of activity.

##### ***Reference*** 

1. Lhullier, C.; Falkenberg, M.; Ioannou, E.; Quesada, A.; Papazafiri, P.; Horta, P.A.; Schenkel, E.P.;Vagias, C.; Roussis, V. Cytotoxic halogenated metabolites from the Brazilian red alga *Laurencia catarinensis*. *J. Nat. Prod.* **2010**, *73*, 27–32.

#### **Studies on the Red Sea Sponge *Haliclona* sp. for Its Chemical and Cytotoxic Properties** 

**Ali El Gamal ^1,2^, Shaza Al-Massarani ^1^, Mansour Al-Said ^1^, Maged Abdel-Kader ^3^, Hazem Ghabbour ^1^, Hoong-Kun Fun ^1^, Wael Abdel-Mageed ^1^, Abdelkader Ashour ^1^, Ashok Kumar ^1^ and Adnan Al-Rehaily ^1^**

^1^ King Saud University, Riyadh, Saudi Arabia

^2^ Mansoura University, El Mansoura, Egypt

^3^ Salman Abdulaziz University, Al-kharj, Saudi Arabia

The Red Sea is characterized by a great diversity of living organisms [1]. Previous chemical studies of marine sponges belonging to the genus *Haliclona* led to the isolation of a variety of bioactive secondary metabolites, including alkaloids [2], macrolides [3], polyacetylenes [4], polyketides [5] and peptides [6]. The total cytotoxic alcoholic extract of a sponge belonging to genus *Haliclona*, collected from the eastern coast of the Red Sea, Jeddah, Saudi Arabia, was subjected to intensive chromatographic fractionation and purification guided by cytotoxic bioassay toward various cancer cell lines. This investigation resulted in the isolation of a new indole alkaloid, 1-(1*H*-indol-3-yloxy) propan-2-ol (**1**), and the previously synthesized pyrrolidine alkaloid, (2*R*,3*S*,4*R*,5*R*) pyrrolidine-(1-hydroxyethyl)-3,4-diol hydrochloride (**4**), isolated here naturally for the first time. In addition, six known compounds—tetillapyrone (**2**), nortetillapyrone (**3**), 2-methyl maleimide-5-oxime (**5**), Maleimide-5-oxime (**6**), 5-(hydroxymethyl) dihydrofuran-2(3*H*)-one (**7**), and Ergosta-5,24(28)-dien-3-ol (**8**) were also identified. The structures of these isolated compounds were elucidated based on extensive examination of their spectroscopic data including 1D and 2D NMR. Compound **5** is reported here for the first time from the genus Haliclona, while compound **7** is not yet reported from a marine source. X-ray single-crystal structure determination was performed to determine the absolute configuration of compound **4**. Unfortunately most of the isolated compounds exhibited weak cytotoxic activity against HepG-2, Daoy, and HeLa cancer cell lines.

##### ***References*** 

1. Lira, N.S.; Montes, R.C.; Barbosa-Filho, J.M. Brominated compounds from marine sponges of the genus *Aplysina* and a compilation of their ^13^C NMR spectral data. *Mar. Drugs*
**2011**, *9*, 2316–2368.
2. Rashid, M.A.; Gustafson, K.R.; Boyd, M.R. A new isoquinoline alkaloid from the marine sponge *Haliclona* species. *J. Nat. Prod.*
**2001**, *64*, 1249–1250.
3. Liu, Y.-H.; Wang, B.; Liu, D.-Y.; Li, L.-D.; Fei, L.N.C. Chemistry and Biological activities of marine sponge *Halichlona*.* JTO*
**2008**, *27*, 70–82.
4. Alarif, W.M.; Abdel-Lateff, A.; Al-Lihaibi, S.S.; Ayyad, S.E.N.; Badria, F.A. A New Cytotoxic Brominated Acetylenic Hydrocarbon from the Marine Sponge *Haliclona* sp. with a Selective Effect against Human Breast Cancer. *Z. Naturforsch. C*
**2013**, *68*, 70–75.
5. Kennedy, J.; Codling, C.E.; Jones, B.V.; Dobson, A.D.W.; Marchesi, J.R. Diversity of microbes associated with the marine sponge, *Haliclona simulans*, isolated from Irish waters and identification of polyketide synthase genes from the sponge metagenome. *Environ. Microbiol.*
**2008**, *10*, 1888–1902.
6. Rashid, M.A.; Gustafson, K.R.; Boswell, J.L.; Boyd, M.R. Haligramides A and B, Two New Cytotoxic Hexapeptides from the Marine Sponge *Haliclona nigra*. *J. Nat. Prod.*
**2000**, *63*, 956–959.

#### **Two New Xenicanes from the Soft Coral *Clavularia* sp.** 

**Carlos Jiménez ^2^, Carlos Urda ^1^, Marta Pérez ^1^, Rogelio Fernández ^1^, Jaime Rodriguez ^2^ and Carmen Cuevas ^1^**

^1^ PharmaMar S.A., Colmenar Viejo, Madrid, Spain

^2^ Universidade da Coruña, A Coruña, Spain

Soft corals belonging to the order Alcyonacea (subclass Octocorallia), are rich sources of xenicane-type compounds which are characterized by a dihydropyran ring fused to nine-membered ring. Four families with different functionalities have been identified within this structural class and they are represented by the xenicins, xenialactols, and xeniolides A and B [1–3]. In this communication, we describe the isolation of two new xenicanes, named PM090004 and PM090082, from a soft coral belonging to the genus *Clavularia*, collected off the coast of Okuza (Tanzania). Only one xenicane has been reported from this genus so far [4]. These new compounds were obtained by column chromatography and semipreparative HPLC purification from an organic extract of this organism. Their planar structures were determined by 1D and 2D NMR and HRESIMS techniques while their relative stereochemistries were elucidated by comparison of their chemical shifts and coupling constants with the literature values of their congeners, as well as by NOESY experiments. Both new xenicanes were moderately cytotoxic against a panel of different tumour cell lines.

##### ***Acknowkedgments*** 

P. J. Ruysenaars (The Pemba channel fishing club Shimoni) and Ministry of Livestock and Fisheries Development. Fisheries Department (Republic of Tanzania).

##### ***References*** 

1. Ishigami, S.; Goto, Y.; Inoue, N.; Kawazu, S.; Matsumoto, Y.; Imahara, Y.; Tarumi, M.; Nakai, H.; Fusetani, N.; Nakao, Y. Cristaxenicin A, an Antiprotozoal Xenicane Diterpenoid from the Deep Sea Gorgonian *Acanthoprimnoa cristata*. *J. Org. Chem.*
**2012**, *77*, 10962–10966.
2. Lin, Y.-S.; Fazary, A.E.; Chen, C.-H.; Kuo, Y.-H.; Shen, Y.-C. Asterolaurins G – J, New Xenicane Diterpenoids from the Taiwanese Soft Coral Asterospicularia laurae. *Helv. Chim. Acta*
**2011**, *94*, 273–281.
3. Andrianasolo, E.H.; Haramaty, L.; Degenhardt, K.; Mathew, R.; White, E.; Lutz, R.; Falkowski, P. Induction of apoptosis by diterpenes from the soft coral *Xenia elongate*. *J. Nat. Prod.*
**2007**, *70*, 1551–1557.
4. Wang, S.; Huang, M.; Duh, C. Cytotoxic constituents from the formosan soft coral *Clavularia inflata* var. *luzoniana*. *J. Nat. Prod.*
**2006**, *69*, 1411–1416.

#### **Multiplicity Editing in Long-Range Heteronuclear Correlation NMR Experiments: Application to Natural Products** 

**Josep Saurí ^1^, Eduard Sistaré ^2^, Michel Frederich ^3^, Alembert T Tchinda ^4^, Teodor Parella ^2^, R. Thomas Williamson ^1^ and Gary E. Martin ^1^**

^1^ NMR Structure Elucidation, Process and Analytical Chemistry, Merck & Co. Inc., Rahway, NJ 07065, USA

^2^ Servei de Ressonància Magnètica Nuclear, Universitat Autònoma de Barcelona, E-08193 Bellaterra, Barcelona, Spain

^3^ Laboratory of Pharmacognosy, Department of Pharmacy, CIRM, University of Liège, B36, 4000 Liège, Belgium

^4^ Center for Studies on Medicinal Plants and Traditional Medicine, Institute of Medical Research and Medicinal Plants Studies (IMPM), P.O. Box 6163, Yaoundé, Cameroon

Even C/CH_2_ and odd CH/CH_3_ carbon-multiplicity information can be directly distinguished from the relative positive/negative phase of cross-peaks in a novel ME (Multiplicity-Edited)-selHSQMBC experiment. The method can be extended by a TOCSY propagation step, and is also fully compatible for the simultaneous and precise determination of long-range heteronuclear coupling constants. In addition, broadband homonuclear decoupling techniques can also be incorporated to enhance sensitivity and signal resolution by effective collapse of *J*(HH) multiplets. Strychnine, taxol, staurosporine, and sungucine are utilized as model compounds to demonstrate the usefulness of these techniques.

#### **Homodecoupled 1,1- and 1,*n*-Adequate NMR Experiments: Application to the Structural Elucidation of Proton-Deficient Natural Products** 

**Josep Saurí ^1^, Wolfgang Bermel ^2^, Alexei V. Buevich ^1^, Maged H.M. Sharaf ^3^, Paul L. Schiff ^4^, Teodor Parella ^5^, R. Thomas Williamson ^1^ and Gary E. Martin ^1^**

^1^ NMR Structure Elucidation, Process and Analytical Chemistry, Merck & Co. Inc., Rahway, NJ 07065, USA

^2^ Bruker Biospin GmbH, Silberstreifen, 76287 Rheinstetten, Germany

^3^ American Herbal Products Association, Silver Spring, MD 20910, USA

^4^ Department of Pharmaceutical Sciences, School of Pharmacy University of Pittsburgh, Pittsburgh, PA 15261, USA

^5^ Servei de Ressonància Magnètica Nuclear, Universitat Autònoma de Barcelona, E-08193 Bellaterra, Barcelona, Spain

Pure shift NMR methods have recently been the subject of intense research focus. By collapsing homonuclear proton-proton couplings, resolution and experimental sensitivity both increase. Cryptospirolepine is the most structurally complex alkaloid discovered thus far from any Cryptolepis. Characterization of several degradants of the original sample a decade after the initial report called the validity of the originally proposed structure in question. We now wish to report the development of improved homodecoupled variants of 1,1- and 1,*n*-ADEQUATE (HD-ADEQUATE) and the utilization of these techniques in resolving long-standing structural questions associated with crytospirolepine. In addition, we evaluate the combination of NUS and homonuclear decoupling for the acquisition of both 1*J*CC and *nJ*CC homonuclear coupling constants in related *J*-modulated ADEQUATE experiments.

#### **Extending Long-Range Heteronuclear NMR Connectivities by Modified HSQMBC Experiments** 

**Josep Saurí ^2^, Núria Marcó ^1^, R. Thomas Williamson ^2^, Gary E. Martin ^2^ and Teodor Parella ^1^**

^1^ Servei de Ressonància Magnètica Nuclear, Universitat Autònoma de Barcelona, E-08193 Bellaterra, Barcelona, Spain

^2^ NMR Structure Elucidation, Process and Analytical Chemistry, Merck & Co. Inc., 126 E. Lincoln Avenue, Rahway, NJ 07065, USA

The detection of long-range heteronuclear correlations associated with *J*(CH) coupling values smaller than 1–2 Hz is a challenge in the structural analysis of small molecules and natural products. HSQMBC-COSY and HSQMBC-TOCSY pulse schemes are evaluated as complementary NMR methods to standard HMBC/HSQMBC experiments. The re-optimization of the interpulse delay and the incorporation of an additional *J*(HH) transfer step in the HSQMBC pulse scheme can favor the sensitive observation of traditionally missing or very weak correlations and, in addition, facilitates the detection of a significant number of still longer-range connectivities to both protonated and non-protonated carbons under optimal sensitivity conditions. A comparative ^1^H–^13^C study is performed using strychnine as a model compound and several examples are also provided including ^1^H–^15^N applications.

#### **The Defensive Chemistry of the Irish Nudibranch *Archidoris pseudoargus *(Gastropoda Opisthobranchia)** 

**Ryan Young ^1,2^ and Bill Baker ^1,2^**

^1^ National University of Ireland, Galway, Co Galway, Ireland

^2^ University of South Florida, Tampa, FL, USA

Historically, marine natural products from the Republic of Ireland have been greatly underrepresented in the literature despite having a coastline of over 4500 miles. *Archidoris pseudoargus* is a soft-bodied, slow moving Dorid nudibranch which inhabits the coastal waters of Ireland and the United Kingdom. Nudibranchs are a good source of new chemical diversity, employing these secondary metabolites to deter predation. In this study we have identified new chemistry as well as used a metabolomics approach to identify the origin of said chemistry. In early Spring, mature adults come together to reproduce and shortly thereafter to oviposit on the subtidal rocky shoreline. These egg sacs can be brightly colored and exposed to predation, yet none of the many surrounding predators appear to feed on these nutrient rich egg masses. We have investigated whether the defensive chemistry of the parents is responsible for protecting the egg masses.

#### **Structural Elucidation of Secondary Metabolites in *Laminaria digitata*** 

**Anne Vissers and Jean-Paul Vincken**

Harry Gruppen Lab. Food Chemistry, Wageningen University, Wageningen, Gelderland, The Netherlands

For future protein supply, *Laminaria digitata *is a promising source due to its relatively high protein content (15% *w*/*w* DM) and robustness in North-Sea growing conditions. In order to apply the seaweed proteins in the feed industry, extraction is needed. The first step in extraction of proteins requires cell rupture, as a result of which cell walls and membranes are broken, allows proteins and secondary defence metabolites to contact each other and interact. Phlorotannins, polymers of 1,3,5-trihydroxy benzene are important secondary metabolites exclusively found in brown seaweeds. The monomers can connect to each other via carbon-carbon and via ether connections and polymers ranging between 10 and 100 kDa are formed (Amsler 2008). Due to the large size and abundance of hydroxyl groups, the molecules can bind non-covalently to proteins via hydrogen bridges and hydrophobic interactions with the tannin rings, and form phlorotannin-protein complexes with limited solubility. An additional group of secondary metabolites is represented by the terpenoids. In order to study the phlorotannin-protein complexation potential during the process of protein extraction, a detailed investigation on phlorotannins content, degree of polymerisation and structure is needed. Chromatographic analysis of phlorotannins forms a challenge due to the structural diversity and similar polarities of these molecules. For investigation, tannins were extracted from *Laminaria digitata *in 80% methanol and partitioned with ethyl acetate. The ethyl acetate phase was subjected to Reversed Phase flash chromatography to separate co-extracted phlorotannins and terpenoids. Pools of phlorotannins up to a degree of polymerisation of 15 subunits, and the terpenoid pool were analysed using RP-UHPLC-MS. The fractionation by preparative chromatography prior to RP-UHPLC analysis greatly facilitated identification and quantification of the various phlorotannin and terpenoid structures.

#### **Initial Attempts in the Purification of the Mediterranean Sponge *Crambe tailliezi*** 

**Siguara B. L. Silva ^1,2^, Erwan Poupon ^2^ and Olivier P. Thomas ^1^**

^1^ Nice Institute of Chemistry-PCRE, UMR 7272 CNRS, University Nice Sophia Antipolis, Parc Valrose, 28 Avenue Valrose, 06108 Nice, France

^2^ BioCIS, Faculté de Pharmacie, University Paris-Sud, 5 rue J-B Clément, 92296 Châtenay-Malabry, France

First described in 1982 by Vacelet and Boury-Esnault, Crambe tailliezi (Crambeidae, Poecilosclerida, Demospongiae) is an encrusting cream sponge found in the Mediterranean and the Macaronesian Sea where its abundance seems to increase along the French Riviera (between 15 and 40 m). Even if the sister species *C. crambe* has been widely studied before, there is no work concerning the isolation and identification of chemicals from *C. tailliezi*. Considering the very interesting alkaloids isolated from Crambeidae sponges, such as the crambescins and crambecidins from *C. crambe*, we decided to investigate the chemodiversity of *C. tailliezi*. The first results on the isolation and structure elucidation of the major compounds present in this species were found to be extremely complicated. However, we were able to recognize guanidine alkaloids similar to the already described batzelladines. This would be the first report of bioactive batzelladine derivatives in a Mediterranean sponge as most of them were found in Caribbean sponges like from species of the genus *Batzella* or Monanchora. The NMR and HRMS analyses also allowed the identification of the already described compounds crambescidin 816 and crambescidin 800. These compounds have been first isolated from *C. crambe* and it is worth noticing that both *C. crambe* and *C. tailliezi* share common secondary metabolites, what could raise interesting questions about the divergent evolutionary history of metabolic pathways in these two sister species.

#### **Bioactive Compounds from Marine Bacteria Recovered from Sediments Collected at the St. Peter and St. Paul Archipelago, Brazil** 

**Alison Batista Silva ^1^, Elthon G Ferreira ^2^, Karine Pires ^2^, Paula C Jimenez ^3^, Letícia Veras Costa-Lotufo ^4^, Otília Deusdência Pessoa ^1^, Edilberto R Silveira ^1^ and Maria Conceição M Torres ^1^**

^1^ Departamento de Química Orgânica e Inorgânica, Universidade Federal do Ceará, Fortaleza, Ceará, Brazil

^2^ Instituto de Ciências do Mar, Universidade Federal do Ceará, Fortaleza, Ceará, Brazil

^3^ Departamento de Ciências do Mar, Universidade Federal de São Paulo, Santos, São Paulo, Brazil

^4^ Instituto de Ciências Biomédicas, Universidade de São Paulo, São Paulo, Brazil

The extension, localization and ecological aspects of the Brazilian coastal zone makes it a remarkable reservoir of compounds with pharmacological potential. Marine actonomycete have shown to be an attractive source of structurally diversified bioactive compounds. The main purpose of this study was to prospect antitumor compounds through a cytotoxicity-guided fractionation approach of the actinomycete strains Salinispora arenicola and *Streptomyces* sp. recovered from sediments collected at the Saint Peter and Saint Paul’s Archipelago (SPSPA)—Brazil. Fractionation of the ethyl acetate crude extract obtained from S. arenicola (IC_50_ 0.55 μg/mL) resulted in two cytotoxic fractions. HR-LCMS dereplication analysis of such fractions combined with comparison in the AntiMarin database indicated the presence of staurosporine derivatives (**1**–**4**), a well known group of anticancer compounds. Chromatographic separations of the inactive fractions led to the isolation of a new rifamycin derivative, 3-(2′-oxo-propyl)-rifamycin S (**5**), in addition to five known metabolites, including 6-methoxy-1-methylisatin, 3-hydroxy-6-methoxy-1-methylindolin-2-one and three diketopiperazines. The structures of these compounds were elucidated through a series of 1D and 2D NMR experiments and HRMS. Bioassay-guided fractionation of the ethyl acetate extract from *Streptomyces* sp. (IC_50_ of 2.27 μg/mL) afforded highly cytotoxic antibiotics piericidin A (**6**) and glycopiericidin A (**7**), as well as three known diketopiperazines from the active fractions. The identification of these compounds was carried out by analyses HR-LCMS, followed by query to the AntiMarin data base. These results highlight the biotechnological potential of the actonomycete strains recovered from St. Peter and St. Paul Archipelago.

#### **Secondary Metabolites and Their Biological Acitivity of the Marine-Derived Fungus *Stemphylium globuliferum*** 

**Jan Schrör ^1^, Peter Hufendiek ^1^, Stefan Kehraus ^1^, Michael Gütschow ^2^ and Gabriele M. König ^1^**

^1^ University of Bonn, Institute of Pharmaceutical Biology, Bonn, Germany

^2^ University of Bonn, Pharmaceutical Institute, Pharmaceutical Chemistry I, Bonn, Germany

Fungi are well known to be a rich source of structurally complex and biologically active compounds. The aim of the current study was to investigate metabolites of the marine-derived fungus Stemphylium globuliferum. The strain was isolated from the brown alga Petalonia zosterifolia collected in the Baltic Sea. Investigation of the extract revealed several metabolites belonging to two structural classes, *i.e.*, dialkylresorcinols and macrolides. Thus, after separation via vacuum liquid chromatography and further purification using HPLC we gained the resorcinol-type metabolite 4-butyl-3,5-dihydroxy-benzoic acid, a new natural product. Another compound isolated was stemphol, a 2,5-dialkylresorcinol. This known metabolite showed activity against multi-resistant *Staphylococcus aureus*, *Enterococcus faecium *as well as against *Candida albicans*. In addition, a weak inhibitory activity toward the protease humane leucocyte elastase (HLE) was found. A third metabolite was the 14-membered lactone coriolide, which was formerly reported from butterflies. This macrolide was active against *Bacillus megaterium*. Furthermore, coriolide displayed a specific inhibitory activity towards HLE with an IC50 of 5 μg/mL, while its activity towards cathepsin B and L was about 10-fold weaker. The isolated metabolites from the fungus *Stemphylium globuliferum* showed a promising antibiotic activity against selected microorganisms as well as towards the protease HLE.

#### **Isolation and Structural Elucidation of Two Novel Pinnatifidenyne-Derived Acetogenins from *Laurencia viridis*** 

Adrián Morales Amador ^1,2^, Caterina Rodríguez de Vera ^1,2^, Olivia Márquez Fernández ^3^, Antonio Hernández Daranas ^1,4^, José Javier Fernández ^1,2^, Manuel Norte ^1,2^ and María Luis Souto ^1,2^

^1^ University Institute of Bio-Organic Chemistry “Antonio González”, Center for Biomedical Research of the Canary Islands (CIBICAN), University of La Laguna, La Laguna 39206, Canary Islands, Spain

^2^ Department of Organic Chemistry, University of La Laguna, La Laguna 39206, Canary Islands, Spain

^3^ Laboratorio de Alta Tecnología de Xalapa LATEX—Universidad Veracruzana, Xalapa, Veracruz, Mexico

^4^ Department of Chemical Engineering and Pharmaceutical Technology, University of La Laguna, La Laguna, Tenerife, Spain

The red alga of the genus *Laurencia *(Rhodomelaceae) include many species widely distributed around the world, being the main red algal genus chemically studied in the last 50 years [1]. They are producers of a vast range of interesting halogenated secondary metabolites, including sesquiterpenes, diterpenes, triterpenes, and acetogenines. Many of these compounds are unique in terms of structural or biological diversity, often exhibiting antibacterial, antifungal, antiviral, antifeedant, antifouling, cytotoxic, antiproliferative, anti-inflammatory, ichthyotoxic and insecticidal activity [2]. As part of our continuing interest on the chemistry of the genus* Laurencia*, we report the isolation of two new C15 acetogenins from *Laurencia viridis *[3,4]. The structures were elucidated on the basis of detailed analysis of 1D and 2D NMR data and revealed that these two compounds are interesting variations on the pinnatifidenyne structure [5].

##### ***Acknowledgments*** 

To MINECO SAF2011-28883-C03-01 and FP7-REGPOT-2012-CT2012-316137-IMBRAIM projects. C.R. de Vera to MINECO FPU program. The research group acknowledge the financing granted to ULL by Agencia Canaria de Investigación, Innovación y Sociedad de la Información, being 85% cofinanced by the European Social Fund.

##### ***References*** 

1. Blunt, J.W.; Copp, B.R.; Keyzers, R.A.; Munro, M.H.G.; Prinset, M.R. Marine natural products. *Nat. Prod. Rep.*
**2015**, *32*, 116–211, and earlier reviews in this series.
2. Wang, B.G.; Gloer, J.B.; Ji, N.Y.; Zhao, J.C. Halogenated organic molecules of Rhodomelaceae origin: Chemistry and biology. *Chem. Rev.*
**2013**, *113*, 3632–3685.
3. Gutiérrez-Cepeda, A.; Daranas, A.H.; Fernández, J.J.; Norte, M.; Souto, M.L. Stereochemical Determination of Five-Membered Cyclic Ether Acetogenins Using a Spin-Spin Coupling Constant Approach and DFT Calculations. *Mar. Drugs*
**2014**, *12*, 4031–4044.
4. Gutiérrez-Cepeda, A.; Fernández, J.J.; Gil, L.V.; López-Rodríguez, M.; Norte, M.; Souto, M.L. Nonterpenoid C15 acetogenins from *Laurencia marilzae*. *J. Nat. Prod.*
**2011**, *74*, 441–448.
5. Norte, M.; Fernández, J.J.; Cataldo, F.; González, A.G. *E*-Dihydrorhodophytin, AC 15 acetogenin from the red alga *Laurencia pinnatifida*. *Phytochemistry*
**1989**, *28*, 647–649.

#### **Cytotoxic Anomoian B and Aplyzanzine B, New Bromotyrosine Alkaloids from Two Indonesian Sponges** 

**Jaime Rodríguez ^1^, Guillermo Tarazona ^2^, Patricia G. Cruz ^2^, Rogelio Fernández ^2^, Marta Penas ^2^, Carmen Cuevas ^2^ and Carlos Jiménez ^1^**

^1^ Departamento de Química Fundamental, Facultade de Ciencias and Centro de Investigaciones Científicas Avanzadas (CICA) Universidade da Coruña, 15071 A Coruña, Spain

^2^ Medicinal Chemistry Department, PharmaMar S.A.U., Pol. Ind. La Mina Norte, Avda. De los Reyes, 1, 28770-Colmenar Viejo, Madrid, Spain

Marine sponges belonging to the order Verongida are devoid of spicules [1], and are difficult to characterize. As such, bromotyrosine derivatives that are characteristic of these types of sponges have been used as chemotaxonomic makers, and have proved a useful tool to facilitate taxonomy identification [2]. Examples of such compounds that have been used for taxonomic work are anomoian A [3] and aplyzanzine A [4]. In a continuation of our investigation of new marine natural products, we now report the isolation of two new bromotyrosine derivatives, anomoian B and aplyzanzine B, that may also have utility as new chemotaxonomic makers and that have been isolated from two different Verongida sponges, collected off the coast of Indonesia. The structures of anomoian B and aplyzanzine B were determined by 1D and 2D NMR experiments and confirmed by high-resolution mass spectrometry. The stereochemistry of the new molecules has been assigned by comparison with literature models [3]. Both compounds showed moderated cytotoxic activity against a panel of different cancer cell lines [5], and their mechanism of action is currently being studied.

##### ***References*** 

1. Ehrlich, H.; Ilan, M.; Maldonado, M.; Muricy, G.; Bavestrello, G.; Kljajic, Z.; Carballo, J.L. Three-dimensional chitin-based scaffolds from *Verongida sponges* (Demospongiae: Porifera). Part I. Isolation and identification of chitin. *Int. J. Biol. Macromol.*
**2010**, *47*, 132–140.
2. Gotsbacher, M.; Karuso, P. New Antimicrobial Bromotyrosine Analogues from the Sponge *Pseudoceratina purpurea* and Its Predator *Tylodina corticalis*. *Mar. Drugs*
**2015**, *13*, 1389–1409.
3. Kottakota, S.K.; Evangelopoulos, D.; Alnimr, A.; Bhakta, S.; McHugh, T.D.; Gray, M.; Groundwater, P.W.; Marrs, E.C.L.; Perry, J.D.; Spilling, C.D.;* et al.* Synthesis and biological evaluation of purpurealidin E-derived marine sponge metabolites: aplysamine-2, aplyzanzine A, and suberedamines A and B. *J. Nat. Prod.*
**2012**, *75*, 1090–1101.
4. Evan, T.; Rudi, A.; Ilan, M.; Kashman, Y. Aplyzanzine A, a new dibromotyrosine derivative from a Verongida sponge. *J. Nat. Prod.*
**2001**, *64*, 226–227.
5. Carter, D.C.; Moore, R.E.; Mynderse, J.S.; Niemczura, W.P.; Todd, J.S. Structure of majusculamide C, a cyclic depsipeptide from *Lyngbya majuscule*. *J. Org. Chem.*
**1984**, *49*, 236–241.

#### **Antimicrobial Metabolites from South African *Laurencia* spp.** 

**Jameel Fakee ^1^, Kim A. Durrel ^2^, Marilize le Roes-Hill ^2^, John J. Bolton ^3^ and Denzil R. Beukes ^4^**

^1^ Rhodes University, Grahamstown, South Africa

^2^ Cape Peninsula University of Technology, Bellville, South Africa

^3^ University of Cape Town, Rondebosch, South Africa

^4^ University of the Western Cape, Bellville, South Africa

The rapid development of resistance against common antibiotics combined with the slow pace of new antimicrobial drug discovery and development present a significant health risk. We have therefore initiated a programme to explore the antimicrobial potential of South African marine organisms. A number of organic extracts from *Laurencia* spp. collected from the South African coast were screened against five biomedically relevant microorganisms, *Acinetobacter baumannii*, *Enterococcus faecalis*, *Escherichia coli*, *Staphylococcus aureus *subsp.* aureus* and *Candida albicans*. One of the more promising extracts was from the red alga, *Laurencia corymbosa* which was selected for further studies. Some 30 metabolites were isolated and characterized by spectroscopic methods. Two of the more active metabolites include the chamigrane (**1**), which showed a minimum inhibitory activity (MIC) of 1 μg/mL against *E. faecalis* while the cuparene (**2**) showed the same level of activity against *A. baumannii*.

#### ***In Vitro* Shistossomicial Activity of the Ascidians *Botrylloides giganteum*, *Didemnum* sp. and *Trididemnum orbiculatum* from Brazil** 

**Luis Claudio Kellner Filho ^1^, Rita Cássia Nascimento Pedroso ^1^, Gustavo Muniz Dias ^2^, Lizandra Guidi Magalhães ^1^, Marcio Luis Andrade e Silva ^1^, Wilson Roberto Cunha ^1^, Patricia Mendonça Pauletti ^1^, Victoria Helen Woolner ^3^, Peter Thomas Northcote ^3^ and Ana Helena Januario ^1^**

^1^ Universidade de Franca, Franca, São Paulo, Brazil

^2^ Universidade Federal do ABC, São Bernando do Campo, São Paulo, Brazil

^3^ Victoria University of Wellington, Wellington, New Zealand

The ascidians *Botrylloides giganteum*,* Didemnum* sp. and *Trididemnum orbiculatum* marine species are widely distributed in the São Sebastião Channel, São Paulo, Brazil. Ascidias (Tunicata, Ascidiacea) have yielded a variety of structurally novel and pharmacologically interesting compounds however, studies related to potential schistosomicidal about these genera are non-existent. Schistosomiasis is one of the most significant neglected diseases in the world. The aim of this research was to evaluate the schistosomicidal potential of the crude methanolic extracts of* B. giganteum* (BG), *Didemnum* sp. (DS) and *T. orbiculatum* (TO) against adult worms of *Shistosoma mansoni.* The extracts were tested for viability and motor activity at the concentrations 50 and 100 μg/mL. Regarding the mobility, TO extract reduced on 100% of motor activity at 50 μg/mL after 72 h, while DS extract decreased the motor activity on 100% of parasites at 100 μg/mL after 24 h. In contrast, the BG extract was inactive. No extract elicited death of the parasites. Despite the inactivity, the proton NMR spectrum of a semi-purirfed fraction from the BO extract revealed interesting signals which prompted further investigation. Combinations of normal and reversed-phase column chromatography and HPLC-ELSD of BG extract conduced the isolation of the aliphatic sulfated compound (3*Z*,6*Z*,9*Z*)-3,6,9-Dodecatrien-1-yl hydrogen sulfate (1). The structural elucidation of 1 was established by NMR spectroscopic and mass spectrometric analysis TOF-MS (−): *m*/*z* 259.1009 (C_12_H_19_O_4_S). This compound was previously isolated as a kairomone secreted by the crustacean *Daphnia magna*.

#### **Search for New Natural Products in Marine Actinomycetales: First Insights into the Secondary Metabolites of *Williamsia maris*** 

**Andrea Lubich ^1^, Liselotte Krenn ^1^, Martin Zehl ^1,2^ and Hanspeter Kaehlig ^3^**

^1^ Department of Pharmacognosy, Faculty of Life Sciences, University of Vienna, Vienna, Austria

^2^ Department of Pharmaceutical Chemistry, Faculty of Life Sciences, University of Vienna, Vienna, Austria

^3^ Institute of Organic Chemistry, University of Vienna, Vienna, Austria

The marine environment is recognized as the space with the highest biodiversity on earth and is hence the richest source for new lead structures in drug development. In this connection, microbes are supposed to produce the largest number and variety of marine secondary metabolites. Among microorganisms, bacteria of the order Actinomycetales are most promising for the elucidation of new structures as 45% of all previously discovered microbial secondary metabolites were isolated from members of this order. This fact prompted us to focus our search for new natural products on marine Actinomycetales. In our project, the scarcely examined strain *Williamsia maris,* which was initially isolated from the Sea of Japan, was obtained from DMSZ (Braunschweig, Germany) and has been cultivated in a GYM-medium (0.4% glucose, 0.4% yeast extract, 1% malt extract) for 14 days. By centrifugation, the cells were removed and the resin Amberlite XAD-16 was added to the fermentation broth to adsorb released metabolites. After two days of incubation, the adsorbate was eluted from the resin with acetone to gain 1.5 g of dried crude extract. This extract was fractionated by SPE on RP-18 cartridges into 18 fractions. Subsequent size-exclusion chromatography led to the isolation of three compounds. Their structures were determined by high-resolution MS and NMR spectroscopy as lumichrome, indole-3-carbaldehyde and a 3-*O*-methyl mannose polysaccharide. LC-MS analysis of another fraction of the extract indicates the presence of two cyclic dipeptides, with the masses and fragmentation patterns correlating with those of cyclo(Ile-Pro) and cyclo(Leu-Pro). All five substances are reported in the strain *Williamsia maris* for the first time.

### **Industrial Biotechnology and Polymers and Biomolecules** 

#### **Biocare *Marine*: Biomolecules of the Sea for Environmental Remediation and Healthcare** 

**Christine Delbarre-Ladrat, Laetitia Kolypczuk, Delphine Passerini, Frédérique Chevalier, Jacqueline Ratiskol, Corinne Sinquin, Agata Zykwinska, Françoise Leroi and Sylvia Colliec-Jouault**

Ifremer, Nantes, France

BioCare marine was a two-year collaborative research project (2012–2014) funded under the EU, Interreg 2-Seas programme. It aimed to isolate, characterise and sustainably utilise marine biomolecules from seaweeds, fish, oysters, shrimps and bacteria. The biomolecules were evaluated to be integrated into functional products for human healthcare and environmental applications. The ocean represents a vast and relatively untapped resource where the organisms therein have evolved a myriad of mechanisms to survive in this changing and demanding environment. These include antimicrobial substances to help out-compete neighbouring organisms, polysaccharides to prevent dehydration and provide structure and defences against toxic metal poisoning. The scientific objectives of Biocare Marine were to: (i) discover and functionalise new antimicrobial compounds (ii) construct novel wound dressings and tissue regeneration scaffolds using marine biomolecules that hold great promise for the treatment of chronic wounds, and for the remodelling and reconstruction of skin in burn victims (iii) utilise the specialised polysaccharides produced by marine bacteria to construct heavy metal capture-systems using an advanced technology platform. Toxic heavy metals cause environmental and health damage and present a significant threat to human wellbeing. Our consortium consisted of the University of Brighton (UK), University of Gent (Gent, Belgium), Ifremer (a French governmental laboratory, Plouzanr, France) and Polymaris (a marine biotech company, Morlaix, France). The poster will be particularly dedicated on bacteria and molecules from Ifremer collection.

#### **Bioremediation of Heavy Metals Using *Cyanothece* sp. CCY 0110 Cultures or Its Released Polysaccharides (RPS)** 

**Rita Mota ^1,2^, Federico Rossi ^3^, Sara B. Pereira ^1^, Ângela Brito ^1,2^, Roberto de Philippis ^3,4^, and Paula Tamagnini ^1,2^**

^1^ i3S—Instituto de Investigação e Inovação em Saúde & IBMC—Instituto de Biologia Molecular e Celular, Universidade do Porto, Porto, Portugal

^2^ Departamento de Biologia, Faculdade de Ciências, Universidade do Porto, Porto, Portugal

^3^ Department of Agrifood Production and Environmental Sciences, University of Florence, Florence, Italy

^4^ Institute of Ecosystem Study (ISE), National Research Council (CNR), Sesto Fiorentino, Italy

Many cyanobacteria can produce extracellular polymeric substances (EPS) that can remain associated to the cell or be released into the environment (RPS-released polysaccharides). The particular features of these polymers, namely the presence of two different uronic acids, sulphate groups and high number of different monosaccharides, makes them promising for biotechnological applications such as metal removal from polluted waters. *Cyanothece *sp. CCY 0110, a marine unicellular cyanobacterium, is among the most efficient RPS-producers. The polymer produced is remarkably thermostable, composed by nine different monosaccharides, and contains sulfate groups and peptides [1]. The effects of several heavy metals on the growth/survival, EPS production, ultrastructure and protein profiles of *Cyanothece *were also evaluated. The results showed that each metal affect the cells in a particular manner, triggering distinctive responses [2]. For optimization of the metal removal process, it is necessary to understand the interactions between cells/EPS with metal ions. Therefore, the affinity of various culture fractions for different metals in mono- and multi-metal systems was assessed. Our results clearly showed that RPS are the most efficient fraction in metal-adsorption. Moreover, an acid or basic pre-treatment of RPS increased the specific metal removal. Currently, major sites for metal binding are being identified by physicochemical analyses.

##### ***References*** 

1. Mota, R.; Guimarães, R.; Büttel, Z.; Rossi, F.; Colica, G.; Silva, C.J.; Santos, C.; Gales, L.; Zille, A.; De Philippis, R.; *et al*. Production and characterization of extracellular carbohydrate polymer from *Cyanothece* sp. CCY 0110. *Carbohydr. Polym.*
**2013**, *92*, 1408–1415.
2. Mota, R.; Pereira, S.B.; Meazzini, M.; Fernandes, R.; Santos, A.; Evans, C.A.; De Philippis, R.; Wright, P.C.; Tamagnini, P. Effects of heavy metals on *Cyanothece* sp. CCY 0110 growth, extracellular polymeric substances (EPS) production, ultrastructure and protein profiles. *J. Proteom.*
**2015**, *120*, 75–94.

#### **Anti-Coagulant Potential of Polysaccharide-Rich Fractions of Macroalgae and Influence of Depolymerization and Sulfation** 

**Amandine Adrien ^1,2^, Nicolas Bridiau ^1^, Delphine Dufour ^2^, Stanislas Baudouin ^2^ and Thierry Maugard ^1^**

^1^ LIENSs Laboratory, UMR 7266 CNRS-University of La Rochelle, La Rochelle, France

^2^ SEPROSYS, Séparations Procédés Systèmes, La Rochelle, France

Macroalgae have been used in traditional medicine for centuries and perceived as a food with great health benefits. They are an important source of polysaccharides, such as sulfated polysaccharides, which are responsible for many of their potential properties that might find relevance in nutraceutical, pharmaceutical and cosmeceutical applications. Nowadays, to treat health troubles linked to thrombo-embolic complication, most of the anticoagulant treatments are heparin-based. Despite its major anticoagulant activity, heparin can cause serious adverse events. Moreover, its low bioavailability makes such a treatment really expensive. Given the risks and high costs of these treatments, there is a compelling need for further investigation of new sources of anticoagulants. To study the anticoagulant potential of sulfated polysaccharides from macroalgae, different extracts were prepared using various extraction processes leading to enriched polysaccharides fractions. Extracts from six edible seaweeds, including brown (*Laminaria digitata, Fucus vesiculosus*, *Himanthalia elongata*, *Ascophyllum nodosum*), green (*Ulva lactuca*), and red (*Chondrus crispus*) macroalgae, were prepared and the biochemical composition of each extract was determined. The potential anticoagulant activity of each extract was also investigated at different scales, from the specific antithrombin-dependent pathway (anti-Xa and anti-IIa) to the intrinsic and/or common (Activated Partial Thromboplastin Time), extrinsic (Prothrombin Time) or common (Thrombin Time) anticoagulant pathways. Furthermore, the extract anticoagulant properties were compared with those of commercial anticoagulants: heparin and Lovenox^®^. To have a better understanding of the structure-function relations for the anticoagulant activity of sulfated polysaccharides, extracts from green macroalgae (*Ulvas*) were also prepared and purified according to a “green” industrial process using neither acid nor solvent. Those polysaccharides were then submitted to chemical modifications (sulfation) or physical modifications (depolymerization assisted by ultrasounds or ion exchange resin).

#### **Identification, Expression and Characterization of Marine Polysaccharide Degrading Enzymes from Novel Bacteria Isolated from Intertidal Biotopes** 

**Varsha Kale ^1,2^, Olafur Fridjonsson ^1^, Sesselja Omarsdottir ^2^, Solveig Petursdottir ^1^, Bryndis Bjornsdottir ^1^, Brynjar Ellertsson ^1^, Solveig Olafsdottir ^1^ and Gudmundur Hreggvidsson ^1,2^**

^1^ Matis ohf, Reykjavik, Iceland

^2^ University of Iceland, Reykjavik, Iceland

Intertidal areas are often covered with algae containing a variety of complex polysaccharides as well as invertebrates harbouring “unconventional” structural polysaccharides. Organisms in these environments need to tolerate extreme conditions, e.g., wave action, radiation and fluctuating temperatures, osmolarity, and pH. Many of the adaptive strategies adopted by the organisms depend on polysaccharides of different structures and properties. Marine polysaccharides contain all the lignocellulose sugars, but also “rare sugars”, e.g., deoxy sugars, sugar acids and sugar alcohols, which are often complex, branched and highly substituted. As the polysaccharides are an abundant source of carbon, microbes from the unique intertidal biotopes are expected to be a valuable source of scientifically interesting and industrially applicable polysaccharide processing enzymes. A number of genomes from novel marine bacteria, isolated from intertidal areas, including geothermal biotopes, were sequenced. Following in-depth bioinformatic analysis, selected genes encoding various carbohydrate enzymes were expressed including enzymes capable of degrading complex marine polysaccharides, e.g., chondroitin sulfate, chitin, laminarin, fucoidan and alginate. Among those were the α-l-fucosidase Aful2 from Litorilinea aerophila MAT4131; chondroitin lyase ChoA1 and the sulfatase SulA2 from the Arthrobacter strain MAT3885. Both strains were isolated from marine environments following enrichment on chondroitin sulfate containing media. Functional analysis of the enzymes revealed that the fucosidase AfuL2 was active on natural substrates such as fucoidan from *Laminaria digitata* and *Fucus vesiculosus*. Addition of the sulfatase SulA2 improved the breakdown of the substrates. Furthermore, the release of fucose from sea cucumber derived fucosylated chondroitin sulfate, induced by AfuL2, was aided by co-incubation with SulA2 and the chondroitin lyase ChoA1. Accordingly, the marine enzymes were capable of reacting with sulfated polysaccharides in a concerted action.

#### **Fucosterol from Padina Australis Protects Sh-Sy5y Cells against Amyloid-Induced Neurotoxicity by Enhancing the Expression of Neuroglobin** 

**Li Zhe Wong ^1^, Sook Yee Gan ^2^ and Eng Lai Tan ^2^**

^1^ School of Postgraduate Studies, International Medical University, Kuala Lumpur, Malaysia

^2^ Life Sciences, School of Pharmacy, International Medical University, Kuala Lumpur, Malaysia

Alzheimer’s disease (AD) is a neurodegenerative disease that leads to progressive loss of neurons which often results in deterioration of memory and cognitive function. The development of AD is highly associated with the formation of plaques within the dying cells in the brain and tangles within the neurons. Recent evidences highlighted the importance of neuroglobin (NGB) in ameliorating AD by protecting the neuronal cells. However, the underlying protective mechanisms remain to be elucidated. This study assessed the ability of fucosterol, a compound isolated from Padina australis, in inducing the expression of NGB gene in neuroblastoma cell line. SH-SY5Y cells were exposed to different concentrations of fucosterol in the presence of beta-amyloid. The effect on apoptosis was determined using Annexin V FITC staining and the expression of NGB was determined using real-time PCR based on SYBR Green. Flow cytometry confirmed the protective effects on SH-SY5Y cells were significant when 5 μg/mL of fucosterol (7.47% double positive for Annexin V and propidium iodide as compared to 9.88% in negative control). Furthermore, results from both real-time PCR and western blot showed that fucosterol significantly induced the expression of NGB in SH-SY5Y with 4 fold increase of NGB expression as compared to the negative control. Finally, we showed that inhibition of NGB through aldrithiol significantly decreased the viability of SH-SY5Y with 41.51% of cell populations under the late apoptosis. This study has thus provided evidences that fucosterol was able to protect SH-SY5Y by enhancing the expression of NGB.

#### **Protein Isolation and Comparison from Various Green Sources** 

**Emma Teuling ^1^, Peter Wierenga ^1^, Johan Schrama ^2^ and Harry Gruppen ^1^**

^1^ Laboratory of Food Chemistry, Wageningen University, Wageningen, The Netherlands

^2^ Aquaculture and Fisheries Group, Wageningen University, Wageningen, The Netherlands

Green sources like algae and leaves are gaining interest as alternative protein sources for food and feed products. These green sources are biologically diverse, ranging from microcellular marine organisms to terrestrial multicellular organisms. The aim of this project is to get more insight into the similarities and differences between green sources and how these differences affect protein isolation and the techno-functionalities of the protein isolates obtained. Protein sources were selected from various unicellular photosynthetic phyla: *Arthrospira* sp. (cyanobacteria), *Nannochloropsis gaditana* (heterokontophyta) and *Tetraselmis* sp. (chlorophyta). The protein contents of these materials were found to be 37%, 42% and 57% for respectively *Tetraselmis*, *Nannochloropsis* and *Arthrospira*. The total carbohydrate content was similar for all sources, ranging from 8% to 11%. Total lipid contents ranged from 15% to 24%. Since all three organisms were protein-rich but contained different types of proteins and carbohydrates, they are good model organisms for comparing protein isolation from unicellular, green sources. A mild protein isolation process was developed, allowing application of the obtained protein isolates in food and feed. After cell disruption 33%, 61% and 80% of the total biomass protein was extracted as soluble protein from *Tetraselmis*, *Nannochloropsis* and *Arthrospira*, respectively. After further purification, the total yield (5%–9%) and purity (66%–74%) of the protein isolates were similar. The microalgal isolates obtained were colourless, while the cyanobacterial protein isolate had an intense blue colour. To conclude, the developed isolation process can be applied to obtain protein isolates from various sources with similar purities and yields. The effects of the differences in protein composition and gross composition of the isolates on the techno-functional properties in food will be assessed by screening their solubility, emulsifying, gelling and foaming properties. These results will provide a better understanding concerning the differences and similarities of proteins from various green sources.

#### **Screening of New Anti-Microbials from Marine Bacteria** 

**Laetitia Kolypczuk ^1^, Christine Delbarre-Ladrat ^1^, Michel Dion ^2^, Delphine Passerini ^1^, Frédérique Chevalier ^1^ and Françoise Leroi ^1^**

^1^ IFREMER—BRM/EM3B, Nantes, France

^2^ University—UFIP, Nantes, France

Marine environment is a rich and relatively little studied source of new functional compounds as antimicrobials. Marine bacteria have developed a range of molecules to survive and colonize this very competitive and challenging environment. We have an important and original collection of more than 4000 marine bacteria strains isolated from deep-sea hydrothermal vents, from seafood products, from microalgae culture and from an hypersaline lake of Kenya. The aim of our study is to screen our collection to discover innovative anti-microbial activities to fight the growing threat of broad spectrum antibiotic resistant infections in human and animals health. All of our bacteria are screened against 15 selected targets bacteria, a range of Gram-positive and Gram-negative bacteria, pathogens for human, for aquaculture organisms or involved in food spoilage. Our first results are promising.

#### **Collagen from Marine Sponges: Comparative Studies on Their Morphological and Biochemical Characterization** 

**Leto-Aikaterini Tziveleka, Evangelia Foufa, Efstathia Ioannou and Vassilios Roussis**

Department of Pharmacognosy and Chemistry of Natural Products, Faculty of Pharmacy, University of Athens, Athens, Greece

Marine organisms have been proven an inexhaustible source of potent bioactive compounds. Between the diverse and multiple pharmacological properties of marine metabolites, bioactive natural macromolecules have gained interest, since such biomacromolecules possess a wide range of biomedical applications. Collagen is a ubiquitous high molecular weight fibrous protein found in all multicellular organisms. Collagen polypeptide chains are organized in three α-helices wrapping around one another forming its characteristic triple helix tertiary structure. This natural biomaterial has a wide range of applications in the health-related fields. Three species of marine demosponges, *Axinella cannabina*,* Suberites carnosus *and *Plakortis simplex*, all collected from the Aegean Sea, were comparatively studied for the determination of their total collagen, intercellular collagen and spongin contents. The isolated collagens were morphologically, physicochemically and biochemically characterized. The yield of total collagen, intercellular collagen and spongin was respectively, 12.6%, 0.1%, and 4.2% dry weight for *A. cannabina* and 5.0%, 0.4%, and 10.4% dry weight for *S. carnosus*, while for *P. simplex *only 0.8% dry weight of spongin was isolated. Light microscopy observations showed fibrous structures, while scanning electron microscopy confirmed the presence of collagen in the isolated samples. The measured IR spectra were characteristic for proteins of the collagen class. The acid-base properties of the material were investigated by titration, which also showed that sponge collagen was insoluble in dilute acid media, while dispersion of collagen was facilitated in dilute basic media. Since collagen from cattle entails the risks of allergic reactions and possible connective tissue disorders, such as arthritis and lupus, and more seriously, of bovine spongiform encephalopathy and transmissible spongiform encephalopathy, a safer source can be marine sponges. Our results suggest that, if efficient aquaculture can be established, *A. cannabina* and *S. carnosus* should be considered as alternative sources of collagen.

#### **Comparison of Microwave- and Freeze-Drying of *Sargassum muticum* for Fucoxanthin Extraction** 

**Elena M. Balboa ^1,2^, Noelia Flórez ^1,2^, Enma Conde ^1,2^, Andrés Moure ^1,2^ and Herminia Domínguez ^1,2^**

^1^ Department of Chemical Engineering, Faculty of Sciences, University of Vigo-Campus Ourense As Lagoas s/n, 32004 Ourense, Spain

^2^ CITI-Universidade de Vigo, Parque Tecnolóxico de Galicia, Rúa Galicia n° 2, 32900 Ourense, Spain

*Sargassum muticum *(Yendo) Fensholt is an invasive brown macroalga in European waters. In order to control its expansion and because its eradication is not feasible, huge amounts of algal biomass are collected from coastal waters with any valuable destination but the direct application to agricultural lands. *S. muticum* (Sm) biomass is available and susceptible to be valorized to obtain new and active fractions and compounds for cosmetic, pharmaceutical and food industries. As a brown macroalgae, Sm is a potential source for carotenoids extraction, as fucoxathin. Several activities for this pigment have been reported: antidiabetic, cholesterol-metabolism control, anti-obesity, antioxidant, cancer cells anti-proliferation, and anti-inflamatory [1]. Fucoxanthin-containing extracts have been obtained by absolute ethanol extraction of freeze-dried Sm as previously reported [2]. Recent data showed an increased availability of the components of Sm algal material by microwave extraction, caused by cell wall structure destruction [3]. Therefore, microwave extraction was proposed as a preparative process before fucoxanthin extraction and as alternative to freeze-drying. Sm was collected (July 2012, Praia Mourisca, Pontevedra, Spain) washed and stored until use. A multimode microwave extractor (NEOS-GR, MilestoneSrl, Italy) was used for algal drying by using different two-steps combinations of time-power profiles (5–10 min/200–800 W). Microwave-dried Sm samples were subjected to ethanol extraction and resulting extracts were compared to those obtained from freeze-dried Sm [2]. Differences on solubles and fucoxanthin extraction yields were determined gravimetrically and by HPLC using a fucoxanthin standard, respectively. Total phenolic content was measured g gallic acid equivalents/g extract [4], and radical scavenging capacity of extracts was expressed as g Trolox/g [5] extract and as IC_50_, DPPH (g/L) [6]. Results are discussed considering the moisture of initial Sm and the preferred use of cheaper and efficient technologies and solvent reduction.

##### ***References*** 

1. Kanda, H.; Kamo, Y.; Machmudah, S.; Wahyudiono, E.Y.; Goto, M. Extraction of fucoxanthin from raw macroalgae excluding drying and cell wall disruption by liquefied dimethyl ether. *Mar. Drugs*
**2014**, *12*, 2383–2396.
2. Conde, E.; Moure, A.; Dominguea, H. Supercritical CO_2_ extraction of fatty acids, phenolics and fucoxanthin from freeze-dried *Sargassum muticum*. J. Appl. Phycol. **2015**, 27, 957–963.
3. Perez, L.; Conde, E.; Dominguea, H. Microwave hydrodiffusion and gravity processing of *Sargassum muticum*. *Process Biochem.*
**2014**, *49*, 981–988.
4. Singleton, V.L.; Rossi, J.A., Jr. Colorimetry of total phenolics with phosphomolybdic-phosphotungstic acid reagents. *Am. J. Enol. Viticult.*
**1965**, *16*, 144–158.
5. Re, R.; Pellegrini, N.; Proteggente, A.; Pannala, A.; Yang, M.; Rice-Evans, C. Antioxidant activity applying an improved ABTS radical cation decolorization assay. *Free Radic. Biol. Med.*
**1999**, *26*, 1231–1237.
6. Von Gadpw, A.; Joubert, E.; Hansmann, C.F. Comparison of the antioxidant activity of rooibos tea (*Aspalathus linearis*) with green, oolong and black tea. *Food Chem.*
**1997**, *60*, 73–77.

#### **Different Methods Fucoidan Extraction from *Sargassum muticum*** 

**Noelia Flórez ^1,2^, María Parada ^1,2^, Elena M. Balboa ^1,2^, M. J. González-Muñoz ^1,2^ and Herminia Domínguez ^1,2^**

^1^ University of Vigo, Ourense, Spain

^2^ Citi-university of Vigo, Ourense, Spain

*Sargassum muticum* (Sm) is a marine brown seaweed that contains alginate, laminaran and sulfated polysaccharides known as fucoidans. Fucoidans are found in the cell walls but the amount varies seasonally and depends primarily on the algae species. Studies have indicated that fucoidans are increasing pharmaceutical interest due to their versatile bioactivity (1). Anticoagulant, antitumor, antiviral, antiinflamatory activities have been studied (2). Crude fucoidans can be extracted from brown algae with different technologies; hydrothermal processing (3) and enzymatic hydrolysis (4) are suitable for the fractionation of vegetal biomass. During these processes, simultaneous extraction and depolymerisation of the fucoidan take place. In this study, two aqueous extraction methods have been proposed and compared for the solubilization and extraction of polysaccharide components and fractions with antioxidant properties from the invasive brown alga *Sargassum muticum*. Liquors obtained from autohydrolysis and enzymatic methods were analyzed and compared. *S. muticum* was collected in Praia da Mourisca (Pontevedra, Spain) in June 2012, washed with water and stored at −18 °C before use. Sm was mixed with water at a liquid:solid ratio of 30:1 (*w*/*w*, dry basis) and heated in a stainless steel reactor up to 170 °C (Parr Instr. Co., Moline, IL, USA), the reactor was quickly cooled and the liquid and solid phases were separated by filtration. The residual alginate in liquid phase was precipitated by adding 1% CaCl_2_ (3). Enzymatic hydrolysis was performed with two different enzymes under different conditions. Liquors from both processes were stored at −18 °C until analysis. The extraction yield was gravimetrically measured and the glucose, xylose, galactose, arabinose and fucose content was determined by HPLC and sulfate content by gelatin-BaCl2 method.

#### **Purification with Resins of the Phlorotannins in the Extracts Obtained by Microwave Hydrodiffusion and Gravity (Mhg) of *Sargassum muticum*** 

**María Parada ^1,2^, Noelia Flórez ^1,2^, Elena M. Balboa ^1,2^ and Herminia Domínguez ^1,2^**

^1^ University of Vigo, Ourense, Spain

^2^ Citi-University of Vigo, Ourense, Spain

*Sargassum muticum* (Sm) is an invasive brown macroalgae in the Atlantic coasts. Different aqueous based extraction processes have been tried to obtain soluble fractions from this algae, including pressurized hot water extraction, enzyme assisted aqueous extraction and microwave hydrodiffusion and gravity (MHG). MHG was selected on the basis of its simplicity and the possibility of using water as solvent or to perform a solvent free process. In this study was to recover phenolic compounds with antioxidant activity and a variety of biological actions from Sm, in a MHG equipment and the selective recovery and concentration of the phenolic compounds from the liquid phase separated has been proposed. Sm was collected in Cape Estai (Pontevedra, Spain) in June 2012, was washed with tap water, freezed and stored in closed plastic bags at −20 °C until use. Operation in microwave NEOS-GR extractor (Milestone S.r.l., Italy) was carried out. Extraction was performed in the presence of light in an open system at maximum temperature of 100 °C. Under these conditions, the phenolic compounds could be stable, and it is also expected that phloroglucinol derivatives would not be affected. Combinations of irradiation power and time during operation, phenolic compounds and radical scavengers were studied. Subsequent purification in commercial food grade non ionic polymeric resins was accomplished. Adsorption onto non-ionic food grade polymeric resins and further desorption with 68% aqueous ethanol solutions were performed operating under previously selected operational conditions. Several commercial food grade resins were screened with the aim of selecting the most suited for the practical recovery of phenolic compounds with radical scavenging activity. Under the optimized adsorption-desorption conditions a powdered yellowish product with 30% phologlucinol content, expressed as g PGE/100 g extract, was obtained and the radical scavenging capacity of one gram of product was equivalent to 1 g of Trolox.

#### **Multi Component Extraction from Scottish Macroalgae** 

**Kirsty Black ^1^^,2^**

^1^ Marine Biopolymers Limited, Ayrshire, UK

^2^ Strathclyde University, Glasgow, UK

Marine Biopolymers Limited (MBL) is an SME founded in late 2009 specialising in the extraction of components from indigenous Scottish seaweeds. MBL has developed a disruptive process for the extraction of alginate, typically the largest component in brown seaweeds, reducing manufacturing time from 24 h or more in a traditional process to less than 12 h. Although its initial main focus is alginate, MBL intends to also bring a range of other, high value, seaweed components to the market, including polysaccharides such as Laminarin, and Fucoidan. The company is also involved in the EU FP7 project, SeaBioTech, and is researching a range of microbial organisms and their metabolites as well as other algae derived compounds with the aim of also commercialising these into high value products.

#### **Preparation of Semisynthetic Derivatives of Bromosphaerol and Evalution of Their Antifouling Activity** 

**Kyriakos Proussis ^1,2^, Efstathia Ioannou ^1^, Theodora Calogeropoulou ^2^, Silvia Morgana ^3^, Veronica Piazza ^3^, Marco Faimali ^3^, Maria Protopapa ^4^, Skarlatos Dedos ^4^, George Verriopoulos ^4^ and Vassilios Roussis ^1^**

^1^ Department of Pharmacognosy and Chemistry of Natural Products, Faculty of Pharmacy, University of Athens, Athens, Greece

^2^ Institute of Biology, Medicinal Chemistry and Biotechnology, National Hellenic Research Foundation, Athens, Greece

^3^ Institute of Marine Sciences (ISMAR), CNR, Genoa, Italy

^4^ Department of Zoology-Marine Biology, Faculty of Biology, University of Athens, Athens, Greece

Biofouling represents one of the most serious problems in modern maritime industries and its control is one of the biggest challenges of marine biotechnology. In search of ecologically friendly, biodegradable antifouling agents, a number of marine natural products were evaluated for their inhibitory activity against the settlement of cyprids of *Balanus amphitrite*. Among them, the brominated diterpene bromosphaerol (**1**), isolated as the main constituent of the red alga *Sphaerococcus coronopifolius*, exhibited prominent inhibitory activity coupled with negligible toxicity against the barnacle nauplii [1]. To gain an insight on the structural features responsible for the antifouling properties of bromosphaerol and to further improve its selectivity index and effective incorporation in marine paints, a number of structural analogues (**2**–**12**) were designed and synthesized. The new derivatives are currently being evaluated for their inhibitory activity against the settlement of barnacle cyprids and for their toxicity against barnacle nauplii.

##### ***Acknowledgments*** 

This work was supported by the project GSRT-EPANII-11ΣYN-5-1274 “MARIPAINTS”, which is implemented under the “COOPERATION 2011” Action of the Operational Programme “Competitiveness and Entrepreneurship” and is co-funded by the European Social Fund (ESF) and National Resources.

##### ***References*** 

1. Piazza, V.; Roussis, V.; Garaventa, F.; Greco, G.; Smyrniotopoulos, V.; Vagias, C.; Faimali, M. Terpenes from the red alga *Sphaerococcus coronopifolius* inhibit the settlement of barnacles. *Mar. Biotechnol.*
**2011**, *13*, 764–772.

